# The cerebrovascular response to norepinephrine: A scoping systematic review of the animal and human literature

**DOI:** 10.1002/prp2.655

**Published:** 2020-09-23

**Authors:** Logan Froese, Joshua Dian, Alwyn Gomez, Bertram Unger, Frederick A. Zeiler

**Affiliations:** ^1^ Biomedical Engineering Faculty of Engineering University of Manitoba Winnipeg Canada; ^2^ Section of Neurosurgery Department of Surgery Rady Faculty of Health Sciences University of Manitoba Winnipeg Canada; ^3^ Department of Anatomy and Cell Science Rady Faculty of Health Sciences University of Manitoba Winnipeg Canada; ^4^ Section of Critical Care Department of Medicine Rady Faculty of Health Sciences University of Manitoba Winnipeg Canada; ^5^ Centre on Aging University of Manitoba Winnipeg Canada; ^6^ Division of Anaesthesia Department of Medicine Addenbrooke’s Hospital University of Cambridge Cambridge UK

**Keywords:** cerebral blood flow, cerebrovascular response, norepinephrine

## Abstract

Intravenous norepinephrine (NE) is utilized commonly in critical care for cardiovascular support. NE’s impact on cerebrovasculature is unclear and may carry important implications during states of critical neurological illness. The aim of the study was to perform a scoping review of the literature on the cerebrovascular/cerebral blood flow (CBF) effects of NE. A search of MEDLINE, BIOSIS, EMBASE, Global Health, SCOPUS, and Cochrane Library from inception to December 2019 was performed. All manuscripts pertaining to the administration of NE, in which the impact on CBF/cerebral vasculature was recorded, were included. We identified 62 animal studies and 26 human studies. Overall, there was a trend to a direct vasoconstriction effect of NE on the cerebral vasculature, with conflicting studies having demonstrated both increases and decreases in regional CBF (rCBF) or global CBF. Healthy animals and those undergoing cardiopulmonary resuscitation demonstrated a dose‐dependent increase in CBF with NE administration. However, animal models and human patients with acquired brain injury had varied responses in CBF to NE administration. The animal models indicate an increase in cerebral vasoconstriction with NE administration through the alpha receptors in vessels. Global and rCBF during the injection of NE displays a wide variation depending on treatment and model/patient.

**Abbreviations**
CBF: cerebral blood flow
NE: norepinephrine
pCO_2_: partial pressure of carbon dioxide
pO_2_: partial pressure of oxygen
rCBF: regional CBF
TBI: traumatic brain injury

## 1 INTRODUCTION

l‐1‐(3,4‐Dihydroxyphenyl)‐2‐aminoethanol or norepinephrine (NE) is an adrenergic drug that is used in a variety of medical care and treatment. It has emerged as one of the most commonly utilized vasopressor agents for general cardiovascular support in the management of critically ill patients, through modulation of adrenergic receptors.^1^, ^2^ Systemically, NE is well known to cause vasoconstriction and in high sustained doses it may lead to limb or end‐organ ischemia.^3^

Despite these concerns regarding systemic vasoconstriction related to NE, it is widely employed, including in those patients with critical neurological illness, such as traumatic brain injury (TBI).^4^, ^5^ However, it remains unclear if detrimental vasoconstrictive responses are seen in the cerebral vasculature with NE administration in human TBI patients. Given that many secondary injury mechanisms in the setting of TBI and other critical neurological illness, resulting in altered or reduced cerebral blood flow (CBF) or impaired cerebrovascular reactivity,^6^, ^7^ understanding the impact of exogenously administered NE on cerebrovascular reactivity and CBF is crucial. Such understanding may impact our choice of vasopressor agent in specific neuropathologic states. Similarly, knowledge here will allow us to anticipate potential cerebral physiologic responses related to NE, as we begin to transition to personalized physiologic targets, particularly in TBI care, based on cerebrovascular reactivity monitoring.^8^, ^9^, ^10^, ^11^, ^12^, ^13^

Human studies evaluating vasopressors and cerebrovascular response in critical neurological illness are inherently confounded by ongoing active treatments for intracranial pressure (ICP), cerebral perfusion pressure (CPP), and other physiologic metrics. As such, focusing on past experimental studies may shed light on the overall impact of NE on cerebrovascular reactivity and CBF, providing a basic understanding of potential expected responses in humans. This will aid the design of future prospective human and large animal model studies on the impact of vasopressor agents on the cerebral vasculature.

The goal of this study was to perform a systematically conducted scoping review of all available literature on the impact of NE on cerebrovascular responsiveness/CBF response, including animal and human studies.

## 2 MATERIALS AND METHODS

A systematic review of the available literature was conducted using the methodology outlined in the Cochrane Handbook for Systematic Reviewers.^14^ The data were reported in line with the Preferred Reporting Items for Systematic Reviews and Meta‐Analyses (PRISMA).^15^ Appendix A of the Supplementary Materials provides the PRISMA checklist. The review questions and search strategy were decided upon by the supervisor (FAZ) and primary author (LF).

### 2.1 Search question, population, and inclusion and exclusion criteria

The question posed for systematic review was: What is the effect of exogenous systemically administered NE on the cerebrovascular response/cerebral blood flow? All studies, prospective and retrospective, animal or human subject, of any size were included. The reason for an all‐inclusive search was the small number of studies of any type identified by the primary author during a preliminary search of MEDLINE.

The primary outcome measure was the impact on CBF or the cerebrovascular responsiveness as documented by autoradiographic diffusible tracer technique, electromagnetic flow probe, freely diffusible tracers, thermal diffusion probe, clearance method, laser‐Doppler flow probe, radioactive gas elimination, radioactive microsphere, flow transducer and flow meter, visual recording software, or any other objective means of CBF determination. Secondary outcomes included adverse effects of NE administration.

All studies, whether prospective or retrospective, of all sizes or of any age category, and with the use of NE with formal documentation of cerebrovascular response/CBF during administration were eligible for inclusion in this review. Exclusion criteria were the following: being a non‐English study or CBF mediation with substance other than NE.

### 2.2 Search strategy

MEDLINE, BIOSIS, EMBASE, Global Health, SCOPUS, and Cochrane Library from inception to December 2019 were searched using individualized search strategies for each database. The search strategy for MEDLINE can be seen in Appendix B of the Supplementary Materials, with a similar search strategy used for the other databases. Finally, the reference lists of reviewed articles on the cerebral blood vessels/CBF response to NE were examined to ensure no references were left out.

### 2.3 Study selection

Using two reviewers (LF and JD), a two‐step review of all articles returned by our search strategies was performed. First, the reviewers independently screened all titles and abstracts of the returned articles to decide whether they met the inclusion criteria. Second, full text of the chosen articles was assessed to confirm whether they met the inclusion criteria and that the primary outcome of CBF/cerebrovascular response to NE was documented. Any discrepancies between the two reviewers were resolved by a third party (FAZ).

### 2.4 Data collection

Data were extracted from the selected articles and stored in multiple electronic databases to ensure data integrity.

### 2.5 Animal studies

Data fields included the following: number of animals, type of study, animal model characteristics, the goal of the study, dose of vasopressors administered, type of vasopressors administered, technique of CBF/vasculature assessment, ventilator parameters (including pCO_2_ and pO_2_—if documented), sedation regimen administered, CBF/cerebral vasculature response to NE, other outcomes and general conclusions.

### 2.6 Human studies

Data fields included the following: number of patients, type of study, patient characteristics, the goal of the study, dose of vasopressors administered, type of vasopressors administered, technique of CBF/vasculature assessment, ventilator parameters (including pCO_2_ and pO_2_—if documented), sedation regimen administered, CBF/cerebral vasculature response to NE, other outcomes and general conclusions.

### 2.7 Bias assessment

Given the goal of this review was to provide a comprehensive scoping overview of the available preclinical literature, a formal bias assessment was not conducted.

### 2.8 Statistical analysis

A meta‐analysis was not performed in this study because of the heterogeneity of model types, study designs, and data.

## 3 RESULTS

### 3.1 Search results and study characteristics

**FIGURE 1:**
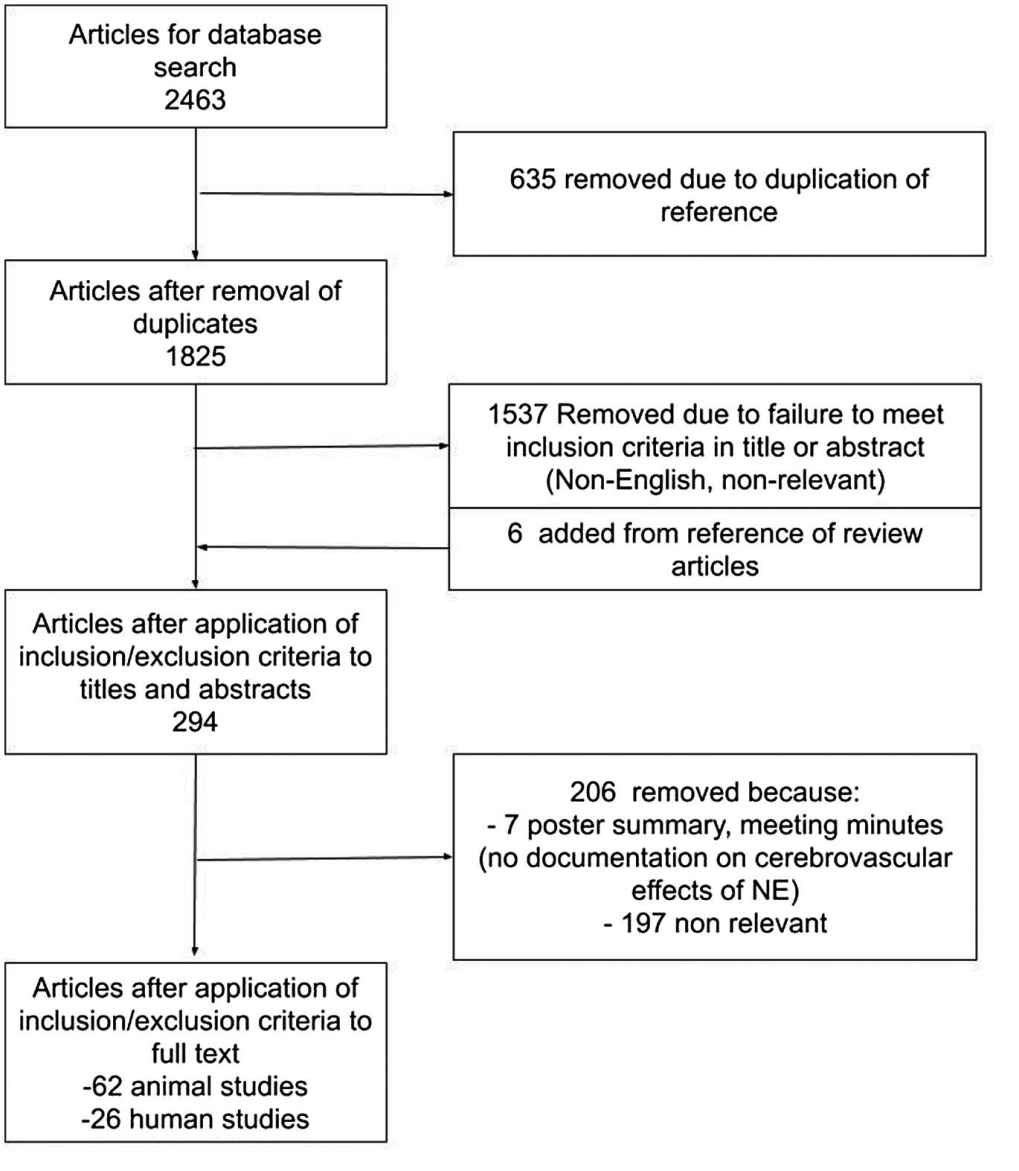
PRISMA flow diagram

The results of the search strategy across all databases and reference sections of articles are summarized in Figure 1. Overall, a total of 2463 articles were identified, all from the databases searched. A total of 635 were removed because of duplication of references, leaving 1825 to review. By applying the inclusion/exclusion criteria to the title and abstract of these articles, we identified 288 articles that fit these criteria. Six articles were added from reference sections of pertinent review articles, leaving a total of 294 papers to review. The portable document formats (PDFs) of these 294 were then gathered. Applying the inclusion/exclusion criteria to these PDFs, only 88 articles were found eligible for inclusion in the systematic review. Articles were excluded because they either: did not report details around the CBF/cerebrovascular response to NE administration or were nonrelevant. One article was a retrospective study focused on CBF and brain function during hypotension and shock.^16^

### 3.2 Animal models

Within the 62 animal studies identified, the majority of cases measured CBF response to NE and other agents, utilizing: autoradiographic diffusible tracer technique, electromagnetic flow probe, freely diffusible tracers, thermal diffusion probe, clearance method, laser‐Doppler flow velocity, radioactive gas elimination, radioactive microsphere, flow transducer and flow meter, visual recording software, and two other methods. The animal models studied included baboons (3),^17^, ^18^, ^19^ cats (8),^20^, ^21^, ^22^, ^23^, ^24^, ^25^, ^26^, ^27^ dogs (11),^28^, ^29^, ^30^, ^31^, ^32^, ^33^, ^34^, ^35^, ^36^, ^37^, ^38^ goats (3),^39^, ^40^, ^41^ pigs (12),^42^, ^43^, ^44^, ^45^, ^46^, ^47^, ^48^, ^49^, ^50^, ^51^, ^52^, ^53^ sheep (1),^54^ mice (1),^55^ rabbits (5),^56^, ^57^, ^58^, ^59^, ^60^ rats (17)^61^, ^62^, ^63^, ^64^, ^65^, ^66^, ^67^, ^68^, ^69^, ^70^, ^71^, ^72^, ^73^, ^74^, ^75^, ^76^, ^77^ and one retrospective study^16^ with dogs, cats, rats, and humans. The characteristics of the animal studies can be seen in Tables 1 and 2. The majority of the models was heavily anesthetized, with only two studies where the animals were lightly sedated^40^, ^42^ and four studies where animals had no sedation or anesthetia.^39^, ^41^, ^55^, ^60^ A further four articles^27^, ^38^, ^44^, ^70^ had NE injected with another vasoactive substance, seven articles^20^, ^23^, ^27^, ^52^, ^53^, ^54^, ^70^ where the models had craniotomy, five articles^34^, ^36^, ^37^, ^73^, ^77^ where models had explanted brains for the evaluation of vessel response, four articles^44^, ^45^, ^46^, ^47^ where models were administered cardiopulmonary resuscitation (CPR) during NE injection, four articles^48^, ^50^, ^51^, ^74^ where models had a TBI, three articles^21^, ^35^, ^66^ where some models had hypothermia, two articles^41^, ^56^ where models had a superior cervical sympathetic ganglionectomy, one article where models had bile duct ligations,^19^ and one article where models had induced endotoxin shock.^43^ Seventeen studies had NE administered at varying dose levels on healthy models.^17^, ^20^, ^26^, ^29^, ^36^, ^39^, ^40^, ^52^, ^53^, ^54^, ^59^, ^63^, ^64^, ^65^, ^69^, ^71^, ^72^ In 23 of the studies the partial pressure of oxygen and carbon dioxide were either controlled through ventilation,^21^, ^25^, ^29^, ^31^, ^35^, ^43^, ^44^, ^45^, ^46^, ^47^, ^48^, ^49^, ^51^, ^53^, ^56^, ^57^, ^60^, ^61^, ^62^, ^66^, ^70^, ^71^, ^73^ or taken to be constant throughout the study in 28.^17^, ^18^, ^19^, ^22^, ^23^, ^24^, ^28^, ^30^, ^32^, ^33^, ^34^, ^36^, ^37^, ^38^, ^39^, ^50^, ^52^, ^54^, ^55^, ^58^, ^59^, ^63^, ^64^, ^65^, ^68^, ^72^, ^74^, ^75^ Ten studies did not mention accounting for the pCO_2_ or pO_2_.^20^, ^26^, ^27^, ^40^, ^41^, ^42^, ^67^, ^69^, ^76^, ^77^

**TABLE 1:** Included studies—general characteristics and study goals

Reference	Number of animals	Study type	Model characteristics	Primary and secondary goals of study
Healthy heavily anesthetized animal models				
McCalden et al, 1979^17^	15 baboons	Three‐arm study	Healthy baboons anesthetized with ketamine hydrochloride and sodium pentobarbital	Primary: Role of catecholamine degradative enzymes and the adrenergic innervation in determining the cerebrovascular response to infused NE
MacKenzie et al, 1976^18^	18 baboons	Two‐arm study	Healthy baboons anesthetized with thiopentone sodium, phencyclidine, and suxamethonium	Primary: Test the effects of NE on cerebrovascular activity
				Secondary: Effect of hypertonic urea
Chandra et al, 1972^20^	Not specified	Four‐arm study	Healthy cats were anesthetized with pentobarbital sodium	Primary: Choroidal blood flow and the effects of autonomic agents
Muravchick et al, 1976^21^	26 cats	Eight‐arm study	Healthy mongrel cats anesthetized with pentobarbital	Primary: Adrenergic receptors and vascular resistance in cerebral circulation
				Secondary: Effect of catecholamines on CBF and CVR
Lobato et al, 1980^22^	Not specified	Nonrandomized control study	Healthy cats intraperitoneally anesthetized with sodium pentobarbital with vessel change measured in removed brains	Primary: Cerebrovascular reactivity to NE and serotonin following experimental subarachnoid hemorrhage
Tomita et al, 1979^23^	23 cats	Four‐arm study	Healthy and cranial hypertensive cats anesthetized with urethane and chloralose	Primary: Distensibility of cerebral vessels in response to acute hypertension
				Secondary: Blood pressure response to NE and papaverine
Haggendal et al, 1966^28^	11 dogs	Three‐arm study	Healthy mongrel dogs anaesthetized with pentobarbital	Primary: Effects of some vasoactive drugs on the vessels of cerebral grey matter in the dog
				Secondary: In a few dogs, similar procedures were performed under the influence of induced slight hypoxia and/or hypercapnia.
Gabrielyan et al, 1970^29^	Not mentioned	Nonrandomized control trial	Healthy dogs that were bleed anesthetized with nitrous oxide and oxygen	Primary: Effect of NE on rCBF depending on initial MAP
MacDonnell et al, 1971^30^	4 dogs	Three‐arm study	Healthy mongrel dogs anesthetized with sodium pentobarbitonal	Primary: Factors affecting response of CBF and cerebral metabolism to NE infusion
James et al, 1975^31^	37 dogs	Seven‐arm study	Healthy mongrel dogs were anaesthetized with sodium pentobarbitonal	Primary: Evaluate factors affecting the cerebrovascular response to NE in the dog
Ekstrom‐Jodal et al, 1974^32^	21 dogs	Two‐arm study	Healthy mongrel dogs anaesthetized with thiopental and nitrous oxide	Primary: Effects of NE on CBF in dogs
				Secondary: Effect of alpha‐adrenergic blockers on NE and CBF
Rogers et al, 1989^42^	21 pigs	Four‐arm study	Healthy piglets anesthetized with halothane and with right common carotid artery ligated	Primary: Influence of intra‐arterial NE on cerebral hemodynamics of newborn pigs
Reynier‐Rebuffel et al, 1986^56^	29 rabbits	Nonrandomized control study	Healthy rabbits—some anesthetized	Primary: Possible mediation of CBF response to systemic NE
Patel et al, 1990^57^	Not mentioned	Three‐arm study	Healthy rabbits anesthetized with 1.0 MAC isoflurane	Primary: CBF and cerebral blood pressure during 1.0 MAC isoflurane anesthesia
Gannushkina et al, 1974^58^	22 rabbits	Two‐arm study	Renal hypertension in healthy rabbits	Primary: Effect of high blood pressure on CBF in renal hypertension
Tomomatsu et al, 1981^59^	62 rabbits	Two‐arm study	Healthy rabbits of either sex anesthetized with urethane	Primary: Increased activity of carotid sinus baroreceptors by sympathetic stimulation and NE
Edvinsson et al, 1979^61^	49 rats	Six‐arm study	Healthy adult male Sprague‐Dawley rats anesthetized with halothane	Primary: Quantitative changes in rCBF of rats induced by alpha and beta‐adrenergic stimulants
Edvinsson et al, 1978^62^	46 rats	Four‐arm study	Healthy Sprague‐Dawley rats anesthetized with halothane	Primary: Effect of exogenous NE on local CBF after osmotic opening of the blood‐brain barrier in the rat
Lasbennes et al, 1988^63^	52 rats	Three‐arm study	Healthy male Wistar rats anesthetized with halothane	Primary: Effect of monoamine oxidase inhibition on rCBF
				Secondary: Effect of clorgyline on cerebral hemodynamics
Szabo et al, 1983^64^	59 rats	Four‐arm study	Healthy male rats anesthetized with pentobarbital sodium and immobilized with gallamine triethiodide	Primary: Effect of sustained NE infusion on CBF
				Secondary: Effect of NE after alpha‐receptor blockade
Tuor et al, 1986^65^	16 rats	Two‐arm study	Healthy male rats anesthetized with halothane	Primary: Effect of hypertensive agent on regional cerebral perfusion
Nemoto et al, 1996^66^	13 rats	Two‐arm study	Healthy male Wistar rats anesthetized with halothane, some given donor blood and induced mild hypothermia	Primary: NE activation of basal cerebral metabolic rate for O_2_ during hypothermia
Sato et al, 1987^67^	4‐6 rats per 4 studies	Four‐arm study	Healthy Sprague‐Dawley rats anesthetized with urethane	Primary: Effect of L‐DOPS vs NE on CBF
Mascia et al, 1999^68^	10 rats	Nonrandomized control study	Healthy Sprague‐Dawley rats anesthetized with halothane	Primary: To investigate the role of the endothelin system in pressure autoregulation of CBF in rats
Stromberg et al, 1992^69^	24 rats	Nonrandomized control study	Healthy male Sprague‐Dawley rats anesthetized with ketamine and acepromazine	Primary: Angiotensin in receptors regulate CBF in rats
Zhang et al, 1991^70^	16 rats	Three‐arm study	Healthy male Sprague‐Dawley rats anesthetized with inactin	Primary: Superoxide dismutase decreases mortality, blood pressure, and CBF responses
Gozzi et al, 2007^71^	35 rats	Four‐arm study	Healthy male Sprague‐Dawley rats anesthetized with halothane and nitrous oxide	Primary: Cerebral hemodynamics and autoregulation in pharmacological MRI
				Secondary: Effect of NE on rCBF and MABP
Kuschinsky et al, 1983^72^	17 rats	Three‐arm study	Healthy male Dawley rats anesthetized with halothane with final values attend from removed brain	Primary: The effects of intravenous NE on the local coupling between glucose utilization and blood flow in the rat brain
Kraut et al, 2004^73^	9 rats	Three‐arm study	Healthy male Wistar rats anesthetized with equithesin	Primary: The effect of NE on tissue areas
Healthy Lightly Anesthetized Animal Models				
Artru et al, 1981^33^	18 dogs	Four‐arm study	Unmedicated fasting mongrel dogs with succinylcholine infusion followed by endotracheal intubation (anesthetized with nitrous oxide, halothane, pentobarbital, or ketamine)	Primary: Anesthetics affect the cerebral metabolic response to circulatory catecholamines
Lluch et al, 1973^39^	15 goats	Five‐arm study	Unanesthetized healthy female goats with thrombosis	Primary: Evidence for effects of adrenergic drugs on CVR
				Secondary: The effect of amines on CBF
Perales et al, 1997^40^	14 goats	Three‐arm study	Conscious female goats sedated with ketamine	Primary: Effects of magnesium sulfate on the NE‐induced cerebral vasoconstrictor and pressor responses in the goat
Von Essen et al, 1972^43^	No Specified	Three‐arm study	Healthy dogs lightly anesthetized	Primary: Effects of dopamine, NE, and 5‐hydroxytryptamine on the CBF in the dog
				Secondary: The effect of dopamine in the presence of pimozide or haloperidol
Edvinsson et al, 1972^55^	124 mice	Two‐arm study	Unanesthetized sympathectomy male albino mice	Primary: Sympathetic neural influence on NE vasoconstriction in brain vessels
Animal models with ganglionectomy				
Alborch et al, 1977^41^	11 goats	Two‐arm study	Unanesthetized female goats with removed cervical ganglion	Primary: Effect of blood flow after removal of cervical ganglion
				Secondary: The effect of NE, tyramine, phentolamine, and propranolol on CBF
Aubineau et al, 1985^60^	7 rabbits	Three‐arm study	Ganglionectomy on rabbit anesthetized by diazepam‐pentobarbital	Primary: Long‐term effects of superior cervical ganglionectomy on cortical blood flow of nonanesthetized rabbits in resting and hypertensive conditions
				Secondary: Effect of NE and Angiotensin II on blood flow
Animal models with bile duct removed				
Bloom et al, 1975^19^	16 baboons	Nonrandomized control study	Bile duct removed in baboon anesthetized with ketamine hydrochloride and portion of them had their bile duct removed	Primary: Modification of the cerebrovascular response to NE by bile duct ligation
Healthy heavily anesthetized animal models with craniotomy				
Shalit et al, 1974^24^	32 cats	Nonrandomized control study	Craniotomy on healthy adult cats anesthetized with pentobarbital with balloon‐induced hypertension	Primary: Interrelationship between blood pressure and rCBF in experimental intracranial hypertension
Ulrich et al, 1985^25^	21 cats	Four‐arm study	Craniotomy on adult cats immobilized with pancuronium bromide and anesthetized with glucochoralose	Primary: In vivo effects of alpha‐adrenoceptor agonists and antagonists on pial veins of cats
Wei et al, 1975^26^	47 cats	Six‐arm study	Craniotomy on anesthetized cats with sodium pentobarbital or urethane	Primary: Determinants of response of pial arteries to NE and sympathetic nerve stimulation
Busija et al, 1987^44^	16 pigs	Prospective randomized animal study	Craniotomy on newborn pigs of either sex 1‐5 days of age were anesthetized with ketamine hydrochloride and acepromazine	Primary: Eicosanoid synthesis elicited by NE in piglet parietal cortex
				Secondary: NE and Isoproterenol effect on cerebral vessels
Leffler et al, 1989^45^	19 piglets	Prospective randomized animal study	Craniotomy on piglets anesthetized with ketamine hydrochloride and acepromazine	Primary: Postischemic cerebral microvascular responses to NE and hypotension in newborn pigs
Myburgh et al, 1998^54^	5 sheep	Three‐arm study	Craniotomy on female sheep, anesthetized	Primary: Comparison of the effect of NE, E, and Dopamine on CBF and COU
Muir et al, 1993^74^	17 rats	Nonrandomized control study	Craniotomy on male Sprague rats anesthetized with sodium pentobarbital	Primary: Cocaine effect on blood pressure and CoBF (cortical) response to NE in rats
Healthy heavily anesthetized animal models with explanted brains				
Oberdorster et al, 1973^34^	14 dogs	Three‐arm study	Dissected canine brains anesthesia with a mixture of allobarbital, urethane, and ethylene urea, coagulation prevented with vetren	Primary: Direct effects of alpha and beta‐sympathomimetic amines on the cerebral circulation of the dog
Lowe et al, 1971^35^	12 dogs	Four‐arm study	Brains from mongrel dogs premedicated with morphine sulfate and anesthetic with methoxyflurane	Primary: Demonstration of alpha and beta‐adrenergic receptors in canine cerebral vasculature
Zimmer et al, 1974^36^	6 dogs	Three‐arm study	Isolated perfused dogs brains which were intravenously anesthetized with a mixture of amobarbital and urethane	Primary: The effect of catecholamine on CBF and oxygen consumption in isolated perfused dog's brain
Omar et al, 2010^75^	About 23 rats for each study	Pharmacological study	Brains of Wistar rats juvenile, mature, and old	Primary: Age‐related changes in the sympathetic innervation of cerebral vessels and in carotid vascular responses to NE in the rat in vitro and in vivo studies
Takahashi et al, 2000^76^	7 rats	Two‐arm study	Brains from male Wistar rats anesthetized with pentobarbital sodium	Primary: The vasoconstrictive action of NE and serotonin in deep arterioles in rat cerebral gray matter
Various animal models				
Mori et al, 1999^27^	34 cats	Three‐arm study	Hypothermia induced in adult cats of both sexes anesthetized with halothane and continuous infusion of ketamine and pancuronium bromide	Primary: Misery perfusion caused by cerebral hypothermia
				Secondary: Effects of vasopressor administration on misery perfusion
Panther et al, 1985^37^	8 dogs	Three‐arm study	Brain cancer dogs anesthetized with sodium pentobarbital	Primary: Vasoactive drugs produce selective changes to blood flow
Nakagawa et al, 1977^38^	21 dogs	Nonrandomized control study	Stereotaxic lesions made on hypothermic dogs anesthetized with thiamylal sodium and lesion	Primary: Role of posterior hypothalamus in the development of acute brain swelling
				Secondary: Lesion effect on ICP
Miller et al, 1984^46^	17 pigs	Three‐arm study	Endotoxin shock induced in healthy pigs anesthetized with ketamine and pentobarbital	Primary: Vasopressors do not increase cerebral cortical blood flow in endotoxin shock
Anesthetized animal models given CPR				
Prengel et al, 2005^47^	21 pigs	Prospective‐randomized animal study	CPR in domestic pigs anesthetized with pentobarbital	Primary: Effects of combined administration of vasopressin, E, and NE during cardiopulmonary resuscitation in pigs
Hoekstra et al 1990^48^	14 piglets	Two‐arm study	CPR on pigs anesthetized with halothane and alpha‐chloralose	Primary: The effect of NE vs E on CBF and myocardial blood flow during CPR
Brown et al, 1989^49^	5 pigs	Three‐arm study	CPR on pigs anesthetized with halothane	Primary: The effect of NE vs E on rCBF during CPR
				Secondary: CBF effect of NE and E in the presence of adrenergic antagonist
Lindner et al, 1990^50^	21 pigs	Three‐arm study	CPR on pigs anesthetized with metomidate and buprenorphine	Primary: The effects of E and NE on cerebral oxygen delivery and consumption during open‐chest CPR
				Secondary: The effects of E and NE on CBF during open‐chest CPR
TBI anesthetized animal models				
Armstead et al, 2016^51^	40 pigs	Three‐arm study	TBI juvenile pigs anesthetized with fentanyl, midazolam, dexmedetomidine, and propofol	Primary: NE’s cerebral autoregulation effects TBI in juvenile pigs
				Secondary: How NE protects cerebral autoregulation
Friess et al, 2012^52^	16 piglets	Three‐arm studies	TBI 4‐week‐old piglets anesthetized with fentanyl and isoflurane	Primary: PE vs NE after noninvasive brain trauma
				Secondary: The effects of PE and NE in the young
Daley et al 2004^53^	6 piglets	Prospective‐randomized animal study	TBI in healthy piglets anesthetized with ketamine and acepromazine	Primary: Assessment of cerebrovascular autoregulation in uninjured and brain‐injured pigs
Ract et al, 2001^77^	14 rats	Three‐arm study	TBI in Sprague‐Dawley rats anesthetized with pentobarbital	Primary: Comparison of dopamine and NE after TBI and hypoxic‐hypotensive insult
Review article				
Kovach et al, 1976^16^	Not applicable	Systematic literature review	Dogs, cats, rats, and humans	Primary: CBF and brain function during hypotension and shock

Abbreviations: AT, Angiotensin II; CBF, cerebral blood flow; CBV, cerebral blood volume; ChBF, choroidal blood flow; CMOT, Catechol‐*O*‐methyltransferase; CMR_Gluc_, cerebral glucose uptake; CMRO_2_, cerebral oxygen consumption; CoBF, corticoid blood flow; COU, cerebral oxygen utilization; CO_2_, carbon dioxide; CP, cerebral perfusion; CPR, cardiopulmonary resuscitation; CPP, cerebral perfusion pressure; CSF, cerebral spinal fluid; CVR, cerebrovascular resistance; E, epinephrine; ERK, extracellular signal‐regulated kinase; FPI, fluid percussion injury; HMF, highest modal frequency; ICP, intracranial pressure; IL‐6, interleukin‐6; keto‐PGFaa, 6‐keto‐prostaglandin; L‐DOPS, l‐threo‐3,4‐dihydroxyphenylserine; L‐NMMA, methylarginine; MABP, mean arterial blood pressure;; MAC, minimum alveolar concentration; MAO, Monoamine oxidases; MAP, mean arterial pressure;; MAPK, mitogen‐activated protein kinase; MBF, mean blood flow; MDo, myocardial oxygen delivery; MRI, magnetic resonance imaging; MVo, myocardial oxygen consumption; NE, norepinephrine; PE, phenylephrine; PGE2, Prostaglandin E2; PO_2_, partial pressure of oxygen; rCBF, regional cerebral blood flow; SAH, subarachnoid hemorrhage; TBI, traumatic brain injury; TXB2, Thromboxane B2; 5‐HT, 5‐hydroxytryptamine;

**TABLE 2:** Norepinephrine Treatment and Cerebrovascular Response—Study Details

Reference	Dose of vasopressor administered	Mean administration	Technique to measure cerebrovascular response	Cerebrovascular response	Adverse effects to norepinephrine	Conclusions
Healthy heavily anesthetized animal models						
McCalden et al, 1979^17^	NE: 0.55 µg/kg/min 1.1 µg/kg/min COMT blockade, MAO blockade, Denervation	60 mins	CBF: Radioactive microspheres with injections of Xenon^133^ CMRO_2_: Calculated with CBF	**PCO_2_ and PO_2_ remained constant throughout all groups** **All values in mL/min/100 g and are the alteration from baseline value** **NE 0.55 µg:** **CBF during:** Control: +9.7 ± 3.6 COMT: −3.3 ± 6.1 MAO: +1.8 ± 4.0 (*P* < .05) Denver: −9.0 ± 7.2 (*P* < .05) **CBF after 10 mins** Control: +11.5 ± 2.2 COMT: −4.6 ± 2.2 (*P* < .05) MAO: +2.1 ± 2.7 (*P* < .05) Denerv: −0.6 ± 4.6 (*P* < .05) **CMRO_2_:** Control: +0.1 ± 0.1 COMT: −0.3 ± 0.6 MAO: −0.4 ± 0.2 Denerv: −0.1 ± 0.6 **NE 1.10 µg:** **CBF during:** Control: +15.5 ± 4.8 COMT: −10.6 ± 0.9 (*P* < .05) MAO: +0.1 ± 2.2 (*P* < .05) Denerv: −6.8 ± 8.2 (*P* < .05) **CBF after 10 mins:** Control: +14.1 ± 3.2 COMT: −7.3 ± 1.6 (*P* < .05) MAO: +0.6 ± 2.3 (*P* < .05) Denerv: −0.5 ± 4.5 (*P* < .05) **CMRO_2_:** Control: +0.2 ± 0.3 COMT: −0.7 ± 0.5 MAO: −0.7 ± 0.2 (*P* < .05) Denerv: +0.1 ± 0.7	None mentioned	The cerebrovascular uptake and degradation mechanisms may be efficient, this remains to be demonstrated by established in vitro technique. The extraneuronal COMT enzyme is important in limiting the access of blood‐borne NE to cerebrovascular constrictor receptors
MacKenzie et al, 1976^18^	NE: 40 µg/kg dissolved in 0.1 m CSF 50 µg/kg/min after hypertonic urea	10 × every 20 mins or 15 s	CBF: Freely diffusible method with Xenon^133^ CMRO_2_: Standard enzymatic assay Cerebral glucose uptake (CMR_glc_): Calculated by CBF *arteriovenous blood glucose difference	**PCO_2_ and PO_2_ remained constant throughout all groups** **NE 40 µg kg:** CBF: Increased by 1 ± 2 mL/100 g/min (P, NS) CMRO_2_: Increased from 2.78 ± 0.10 to 3.44 ± 0.42 mL/100 g/min (*P* < .05) CMR_glc_: Increased from 4.21 ± 0.42 to 10.65 ± 2.96 mg/100 g/min(P, NS) No significant changes in CMRO_2_, CMR_glc_, CBF, or MAP **Hypertonic Urea:** CBF: Decreased by 3 ± 3 mL/100 g/min (P, NS) CMRO_2_: Decreased by 0.04 ± 0.18 mL/100 g/min (P, NS) CMR_glc_: Decreased by 0.33 ± 0.4 mg/100 g/min (P, NS) **NE and Hypertonic Urea:** CBF: Increased by 26 ± 7 mL/100 g/min (*P* < .02) CMRO_2_: Increased by 0.79 ± 0.11 mL/100 g/min (*P* < .001) CMR_glc_: Increased by 4.84 ± 1.67 mg/100 g/min (*P* < .05).	None mentioned	In two studies there was not any decrease in cerebral blood flow associated with the administration of NE. Once NE gains access to the cerebral interstitial fluid it would appear that the dominant circulatory response is vasodilation, this being accompanied by increased oxygen and glucose utilization by the brain
Chandra et al, 1972^20^	Levarterenol: 0.1‐10 µg E: 0.5‐1 µg Acetylcholine: 1‐10 µg Isoproterenol: 0.01‐1 µg	Not specified	Choroidal blood flow (ChBF): Krypton^85^ Clearance	**PCO_2_ and PO_2_ assumed to be constant throughout all groups** **Levarterenol:** Lateral long posterior axillary artery injection (LLI) low dose: CVR: +32% ChBF: −36% higher dose: CVR: +155% ChBF: No significant changes Femoral artery injection: CVR:+7% ChBF: +119% **E:** CVR: +33% ChBF: −38% Systemic injection of low doses: CVR: +15% ChBF: −16% High doses: CVR: −11% ChBF: +43% **Acetylcholine Low dose:** CVR: −35% ChBF: +30% High dose: CVR: −40% ChBF: +27% Systemic injection CVR: −38% ChBF: +18% **Isoproterenol:** Lateral long posterior axillary artery injection: CVR: +7% ChBF: −27% decrease Femoral artery injection: CVR: Variable effect ChBF: −30%	None mentioned	Autonomic agents have significant effects on CVR and ChBF indicating the presence of alpha and gamma receptors. In this respect, the choroidal vascular bed resembles that of other tissues except for the brain and retina. In contrast, isoproterenol does not seem to have an appreciable effect on CVR indicating the absence of beta receptors
Muravchick et al, 1976^21^	NE: 0.5 µg/kg E: 1.0 µg/kg Isoproterenol: 2.0 µg/kg Histamine: 3.0 µg/kg	10‐15 sec	CBF: Electromagnetic flow transducer and flow meter CVR: Calculated by net driving perfusion pressure/observed perfusate flow rate	**PCO_2_ and PO_2_ remained constant throughout all groups** **NE no blockade:** CBF:−21.2 ± 2.0 (−25%) mL/min/100 g CVR: +1.4 ± 0.9 (+82%) mmHg/mL/min/100 g **NE alpha blockade:** CBF: −8.8 ± 2.0 (−8%) mL/min/100 g CVR: +0.1 ± 0.0(+10%) mmHg/mL/min/100 g **Isoproterenol no blockade:** CBF: +16.0 ± 1.2(+21%) mL/min/100 g CVR: −0.4 ± 0.1(−22%) mmHg/mL/min/100 g **Isoproterenol beta blockade:** CBF: 0.0 ± 3.6 (0%) mL/min/100 g CVR: 0.0 ± 0.1 (0%) mmHg/mL/min/100 g **E no blockade:** CBF: −24.8 ± 2.0”(−29%) mL/min/100 g CVR: +1.0 ± 0.2(+62%) mmHg/mL/min/100 g **E alpha blockade:** CBF: −6.8 ± 1.6”(−7%) mL/min/100 g CVR: +0.2 ± 0.1(+14%) mmHg/mL/min/100 g **Histamine no blockade:** CBF: 35.7 ± 10.6 (49%) mL/min/100 g CVR: −0.6 ± 0.2(−30%) mmHg/mL/min/100 g **Histamine beta blockade:** CBF: 27.8 ± 2.9 (36%) mL/min/100 g CVR: −0.5 ± 0.0 (−28%) mmHg/mL/min/100 g **Histamine alpha block:** CBF: 10.0 ± 2.4 (13%) mL/min/100 g CVR: −0.2 ± 0.1 (−11%) mmHg/mL/min/100 g	None mentioned	The wide variation in absolute values of initial CVR presented in the data obtained with this preparation reflects the great sensitivity of the cerebral vasculature to the quality of the immediate biochemical and physical environment. The vasoconstrictor or vasodilator substance is a function of the initial vascular resistance NE demonstrated a general increase in CVR with a subsequent decrease in CBF
Lobato et al, 1980^22^	NE: 10−8 to 10^−4^ (mol/L) 5‐HT: 10^−8^ to 10−5 (2.5 mol/L)	Readjusted every 15 mins during an equilibration period of 90 to 120 mins	Isometric vascular responses: Grass force‐displacement transducer	NE induced a dose‐dependent contractile response of the posterior communicating arties of normal cats. This response was significantly reduced (*P* < .02) in a competitive manner by phentolamine (10‐6 mol/L), an alpha‐adrenergic blocker For both NE the increase in the developed tension increases on a 0‐300 mg tension, for all except SAH 3 days and ganglionectomy which both increase at the same rate from 100 mg to 500 mg or 140 to 500 mg For both 5‐HT the increase in the developed tension increases on a 0‐200 mg to 300‐700 mg tension, for all except SAH 3 days and ganglionectomy which both increase at the same rate from 300‐1400 mg or 200‐500 mg	None mentioned	Super sensitivity to NE and serotonin induced by subarachnoid hemorrhage (SAH) may be involved in the production of chronic cerebral vasospasm
Tomita et al,1979^23^	Papaverine hydrochloride: 10 mg/kg (n = 6) NE:10 µg/kg (n = 9) NE and acute brain swelling: 10 µg/kg (n = 8)	To raise MABP to 150 mmHg	CBF: Calculated from CBV*density of brain tissue CBV: Photodiode and polygraph ICP: Strain gauge transducer	**PCO_2_ and PO_2_ were kept constant throughout all groups** **Papaverine hydrochloride:** ICP: Slight increase CBV: Increased by 1.4% **NE:** Decrease in CBV in a cat without any premeditation, indicating that NE constricted the "inexperienced" cerebral vessels (*P* < 0) **NE and acute brain swelling:** CBV: Increased by 0.8 ± 0.3% ICP: Increased by 15.3 ± 3.3 mmHg CBF: 91 to 101 mL/100 g.min	None mentioned	Intravenous administration of NE to papaverine‐pretreated cats produced almost maximal distension of the cerebral vessels, together with simultaneous vasoconstriction in the peripheral vessels, giving rise to an uneven redistribution of blood between the brain and other nonessential organs of the body NE has an indication to constrict the brain vessels though this does not translate to a direct increase in CBF or ICP
Haggendal et al, 1966^28^	Papaverine: 20‐80 mg (n = 6) 1‐10 mg/kg/body weight Papaverine and Aramine: 2 mg/kg and 30 µg/kg/min (n = 4) Aramine: 200‐500 µg/mL (n = 8) infused at 1.5‐40 µg/kg/body weight/min NE: 10‐50 µg/mL (n = 3) infused at 0.2‐3 µg/kg/bodyweight/min	MAP: kept at 200 mmHg	CBF: Krypton^85^ clearance method CVR: MAP/CBF	**PCO_2_ and PO_2_ were kept constant throughout all groups** **Aramine:** CBF: Reduced to 11 mL/100 g/min CPP: Increased **Aramine flow doubled:** CBF: Reduce by 70% CVR: 160% of control MAP: Increased by 50% **Papaverine and Aramine:** CBF: Increased to 160 mL/100 g/min CVR: Decreased to 1 mmHg*100 g*min/mL MAP: Constant at 180 mmHg **NE 4 µg/kg:** CBF: Decrease by about 40% CVR: 3 × increase **NE 1 µg/kg:** CBF: Decrease by about 40% CVR: 3 × increase **Hypotensive state:** CBF was unchanged compared with Aramine and NE thus indicating dilatation of the cerebral vessels as response to the decreased perfusion pressure. **Papaverine 2 mg/kg:** CBF: Decrease by 10% CVR: Decreased by 0.4× Aramine provoked increase of CVR also existed when papaverine was given, although to a reduced extent	None mentioned	Aramine and NE, given as intravenous infusions in previous doses, had qualitatively similar actions on the cerebral circulation in dogs although NE consistently seemed to have a more potent vasoconstrictor effect. The cerebral vasoconstrictor effect of the pressor drugs were observed during slight hypoxia and/or hypercapnia. Papaverine was found to cause a marked vasodilatation of the cerebral vessels which also was obvious although less pronounced with Aramine
Gabrielyan et al, 1970^29^	NE: 24 µg/min	Not specified	rCBF: Freely diffusible tracer Krypton^85^ infusion and correlated with PCO_2_ Blood Flow: Micro‐Astrup instrument	**PCO_2_ and PO_2_ were constant throughout all groups** **Control:** CBF: 0.85 ± 0.016 mL/g/min, Cerebral Resistance: 1.7 ± 0.09 mmHg/mL/100 g/min. **Low hypotension caused by bleeding:** rCBF: Remained unchanged Cerebral Resistance: Decreased 1.28 ± 0.009 mmHg /mL/100 g/min (*P* < .001) **NE low hypotension:** CBF: Reduced by 0.60 ± 0.023 mL/g/min (*P* < .001) Cerebral Resistance: Increased by 2.4 mg/mL/100 g/min	None mentioned	NE on the rCBF is largely dependent on the initial value of the mean arterial pressure. Whereas in normotension, in response to injection of NE the CBF remains almost unchanged, in moderate hypotension it is considerably reduced
MacDonnell et al, 1971^30^	NE 0.4 and 1 µg/kg/min Propranolol: 5 mg NE 1 µg and Propranolol 5 mg	Several hrs	CBF: Freely diffusible tracer injection of Krypton^85^ CMRO_2_: Oxygen electrode	**PCO_2_ and PO_2_ were kept constant throughout all groups** **NE 0.4 µg:** CBF: Slight drop CMRO_2_: Slight drop **NE 1.0 µg:** CBF: Slight drop CMRO_2_: Slight drop **Propranolol:** CBF: Decrease 20% CMRO_2_: Decrease 10% **Propranolol and NE:** CBF: Decrease 40% CMRO_2_: Decrease 30%	None mentioned	NE slightly decreased CBF, NE with propranolol caused a more prominent fall in CBF then just NE
James et al, 1975^31^	NE: 0.1‐1 µg/kg/min Propranolol: 0.4 µ g/kg/min Phenoxybenzamine: 1‐10 mg/kg	15 to 60 mins	Cortical blood flow (CoBF): Freely diffusible tracer injection of Krypton CMRO_2_: Product flow and the arteriovenous difference	**PCO_2_ and PO_2_ were constant throughout all groups except CO_2_ modulated** **Control:** CoBF: 108.6 ± 9.0 mL/100 g/min CMRO_2_: 10.9 ± 1.1 mL/100 g/min **NE control:** CoBF: Increased by upto 130% CMRO_2_: 15.2 ± 2.90 mL/100 g/min Dose greater than 0.1 µg/kg/min had little further effect on CoBF **NE and CO_2 _modulation:** CoBF: 93 mL/100 g/min CMRO_2_: Fell compared to control **NE and propranolol:** CoBF: −60% **NE and phenoxybenzamine:** CoBF: −125%	None mentioned	Cerebral vasodilatation observed following intravenous NE is relaxed and is triggered by chemoreceptors activity. Antagonism of the cortical dilatory effects if intravenous NE by raised PaCO_2_ is the intact animal must be at a site different from the peripheral chemoreceptors
Ekstrom‐Jodal et al, 1974^32^	NE: 0.03 to 7.5 µg/kg/min Phentolamine: 0.3‐15 mg/kg/min	NE dissolved into 50 µg/mL Dopamine dissolved in 10 mg/mL	CBF: Radioactive gauss elimination method Krypton^85^ 1.5 hrs was waited till first measure was taken	**PCO_2_ was low in most models with some having a high value 80 mmHg** **NE:** CBF: Max change any dose above 2 µg/kg/min at 20% CMRO_2_: Reduced 40% to 70% **NE and Phentolamine:** CBF: Alpha‐adrenergic receptors blocked so no flow reduction	None mentioned	NE induced a flow reduction which seemed to be already maximal at a fairly low infusion rate of below 2 µg/kg/min. The blood flow reduction was practically the same in normo‐ and hypercapnia
Rogers et al, 1989^42^	NE: 100 ng/min (n = 11) Propanol: 1 mg/kg (n = 5) Prazosin: 1 mg/kg and Yohimbine: 1 mg/kg (n = 5)	Two 5 mins infusions	CBF: Radiolabeled microsphere technique CMRO_2_: Blood gas analyzer	**PCO_2_ and PO_2_ were kept constant throughout all groups** **NE:** CBF: 72 ± 5 to 82 ± 8 mL/10 g/min Cerebral Oxygen consumption: 2.75 ± 0.17 to 3.11 ± 0.29 mL *O_2_/100 g/min **NE and propranolol:** No significant effect **NE + prazosin + yohimbine:** Limits of CBF and O_2_ consumption	None mentioned	Circulating NE may increase CBF via beta‐adrenergic‐mediated stimulation of cerebral oxygen consumption during severe stress
Reynier‐Rebuffel et al, 1986^56^	NE: 1 µg/kg/min	35 sec	CBF: Autoradiographic diffusible tracer technique with C‐14 ethanol	**PCO_2_ and PO_2_ were kept constant throughout all groups** **NE unanesthetized:** CBF: No significant change in cortical regions but the flow decrease 6 to 22% in other structures which were significant in nucleus, hypothalamus, colliculus, and reticular **NE anesthetized group 1:** Same as unanesthetized but in superior colliculus the response was inverted leading to significant increase in blood flow **NE anesthetized group 2:** General increase in CBF except caudate nucleus	None mentioned	Showed that caudate nucleus but not thalamic or cortical regions reaction to circulating NE which can be specifically differentiated from the classical autoregulatory response to BP. Under anesthetized these changes in cerebrovascular reactivity appear to be linked to moderate change in systemic reactivity
Patel et al, 1990^57^	Angiotensin II (AT): 20 µg/mL NE: IV 32 µg/mL PE:120 µg/mL	Used to increase MAP to 20%, 40%, 60% and 80%	CBF: Radiolabeled microsphere technique	**PCO_2_ and PO_2_ were kept constant throughout all groups** All values in mL/g/min **AT:** CBF: 0.78 ± 0.07 Hemispherical CBF: 0.75 ± 0.07 Posterior Fossa CBF: 0.86 ± 0.06 **NE:** CBF: 0.67 ± 0.04 Hemispherical CBF: 0.65 ± 0.04 Posterior Fossa CBF: 0.75 ± 0.05 **PE:** CBF: 0.73 ± 0.06 Hemispherical CBF: 0.70 ± 0.05 Posterior Fossa CBF: 0.82 ± 0.07	None mentioned	NE and PE may indirectly result in cerebrovascular vasodilation or AT has intrinsic cerebral vasoconstrictive effects during isoflurane anesthesia and therefore the cerebrovascular autoregulation should affect selected vasopressor
Gannushkina et al, 1974^58^	NE: 10 mL of a 0.02% solution	2‐3 mins	CBF: Hydrogen clearance method	**PCO_2_ and PO_2_ were assumed to be constant throughout all groups** **NE:** CBF: Dropped from 108 to 32 mL/100 g/min then remained stable **NE and Renal Hypertension:** CBF had a slight increase at injection (182 mL/100 g/min; *P* < .01), which then fell sharply to 40%‐50% of its initial value (32 mL/100 g/min *P* < .01) In two animals there was the same rise as control	None mentioned	Raising the pressure in control rabbits above 160‐180 mmHg led to an increase in the CBF; in the rabbits with experimental renal hypertension this increase in blood flow began at higher levels of the arterial pressure and was quickly followed by a decrease to 40%‐50% of the initial blood flow
Tomomatsu et al, 1981^59^	NE: 10^−9^ to 10^−5^ g/mL Phentolamine: 10^−6^ g/mL	10 mins	Tension: Isometer transducer Pressure: Electrode manometer	**PCO_2_ and PO_2_ remained constant throughout all groups** **Phentolamine:** Concentration of 10^−6^ g/mL completely abolished the responses to NE (10^−9^ to 10^−7^ g/mL) how at higher volume NE tension increase maximum of 40% **NE:** Linear increase in tension from 0% to 100% as dose increased	None mentioned	In the presence of 10^−9^ g/mL NE, discharge frequency of all units significantly increased at a given pressure step when compared with the control, whereas NE at a high concentration (10^−6^ g/mL) did not produce significant changes in the discharge frequency. It is concluded that NE released by sympathetic nerve endings most likely acts directly on the baroreceptor nerve endings and sensitizes them
Edvinsson et al, 1979^61^	L‐arterenol hydrochloride: 1 µg/kg/min L‐epinephrine bitartrate: 1 µg/kg/min L‐isoproterenol hydrochloride: 0.5 µg/kg/min Phentolamine:1 µg/kg/min	01 mL/min at 10 mins	rCBF: Autoradiographic diffusible tracer technique with C‐14	**PCO_2_ and PO_2_ were kept constant throughout all groups** **NE:** No significant effect in thalamus mesencephalon and pons, all other region the CBF was reduced by 10%‐27% (*P* < .05) **E:** CBF changes similar to that of NE but not significant **Phentolamine + NE:** Prevented any change to CBF **Phentolamine + E:** Vascular response markedly reversed, pons 92% and thalamus 74%, and mesencephalon 45%‐46% (*P* < .001) **Propranolol + isoprenaline:** Clear‐cut increases in regional blood flow were found in pons, mesencephalon, thalamus, and caudate nucleus. The cortical regions and cerebellum only showed a tendency to flow increase, which was not statistically significant	None mentioned	The presence and heterogenous distribution in the cerebrovascular bed of alpha‐ and beta‐adrenoceptors that can be activated by sympathomimetics given systemically. If NE was allowed to pass the blood‐brain barrier after osmotic opening with urea, an increased regional flow was obtained, probably due to a mechanism where the vasodilator effect secondary to activation of cerebral metabolism predominated over the direct vasoconstrictor effect of the amine
Edvinsson et al, 1978^62^	NE: 5 µg/kg/min Propranolol: 25 µg/kg/min	10 mins	CBF: Autoradiographic diffusible tracer technique with C‐14 ethanol	**PCO_2_ and PO_2_ were kept constant throughout all groups** **Brain region: (Base line, After urea) mL/100 g/min** Parietal cortex: 4.7 ± 0.4, 16.6 ± 3.0 (*P* < .01) Occipital cortex: 4.5 ± 0.5, 17.5 ± 3.6 (*P *< .01) Caudate nucleus: 2.8 ± 0.4, 12.5 ± 3.0 (*P* < .01) Thalamus: 2.7 ± 0.4, 10.9 ± 2.6 (*P* < .05) Mesencephalon: 39 ± 0.5, 3.9 ± 0.5 (*P*> .05) **NE and hypertonic urea:** CBF: Significant increase over 10 mL/100 g/min in every area but mesencephalon on injection side as compared to noninjection **NE and hypertonic urea and propranolol:** CBF: Negated effects of NE	None mentioned	The normally low penetration of NE into the brain was enhanced fourfold in those brain regions that showed Evans blue extravasation following the administration of hypertonic urea. In the same regions, the systemic administration of NE markedly increased local CBF, compared to the contralateral hemisphere that was unaffected by the injection of urea. This effect on rCBF was blocked by the beta‐receptor antagonist, propranolol
Lasbennes et al, 1988^63^	NE: 10 µg/mL (N = 20) Clorgyline: 1 mg/kg (n = 9) Clorgyline and NE: 1.9 mg/kg and 1.5 µg/kg/min (n = 8)	To achieve MAP of 121 and 171 mmHg	rCBF: Autoradiographic diffusible tracer technique with iodo‐antipyrine	**PCO_2_ and PO_2_ were constant throughout all groups** **Only Clorgyline with NE had statistically significant rCBF:** Frontal Cortex: 18 ± 5 (*P* < .05) Parietal Cortex: 15 ± 5 (*P* < .05) Thalamus: 14 ± 5 (*P* < .05) Mesencephalon: 15 ± 5 (*P* < .05) Pons: 16 ± 5 (*P* < .05) **NE:** rCBF and MAP showed linear relationship at large infusions produced substantiation increase in CBF **Clorgyline:** No significant effect to CBF or blood‐brain barrier perfusion at any injection amount	None mentioned	Clorgyline administration alone did not significantly modify rCBF, but the subsequent infusion of NE induced an increase in rCBF in all structures investigated
Szabo et al, 1983^64^	NE: 10 µg/kg/min 2 hrs (n = 8) 20 µg/kg/min 1 hrs (n = 11) 20 µg/kg/min 2 hrs (n = 11) Phenoxybenzamine and NE: 5 mg/kg and 20 µg/kg/min for 2 HR (n = 10)	1‐2 hrs	CBF: Autoradiographic diffusible tracer technique with C‐14 labeled iodo‐antipyrine CVR = MAP/CBF	**PCO_2_ and PO_2_ were constant throughout all groups** **Control:** CBF: 0.86 ± 0.03 mL/min/g CVR:1.70 ± 0.06 mmHg*min*g/mL **NE 10 µg:** CBF: 1.18 ± 0.05 (*P* < .001) CVR: 1.49 ± 0.07 (*P* < .05) **NE 20 µg for 1 hrs:** CBF: 0.91 ± 0.04 CVR: 2.39 ± 0.12 (*P* < .001) **NE 20 µg for 2 hrs:** CBF: 0.66 ± 0.05 (*P* < .001) CVR: 1.8 ± 0.11 **Phenoxybenzamine and NE:** CBF: 1.48 ± 0.07 (*P* < .001) CVR: 0.94 ± 0.05 (*P* < .001)	Lethal outcome of shock with sustained NE blood concentrations and for infusions over 20 µg/kg/min longer than 2 hrs effectively prevent cerebral autoregulation	Supports the hypothesis that high concentrations of NE in cerebral blood vessels produced by activity might be an important factor in etiology of blood flow deficiencies
Tuor et al, 1986^65^	L‐NA: 1‐15 µg/kg, Dopamine: 75‐300 µg/kg/min	ABP maintained at 35 mmHg	CBF: Autoradiographic diffusible tracer technique with C14 iodo‐antipyrine	**PCO_2_ and PO_2_ were constant throughout all groups** **NE 5 µg:** CBF auditory cortex: Decreased by 18 ± 5% CBF cerebellar vermis: Increased by 66 ± 29% CBF pontine reticular: Increased by 38 ± 13% CBF median: 15% (*P* < .05) **Dopamine:** CBF in rostral cerebral cortex, posterior parietal cortex and white matter: Greater than 65% (*P* < .05) CBF Nuclei of lower brain stem: Less than 40% (*P* < .05) CBF median: 44%	None mentioned	The cerebrovascular response to hypertension appears to be dependent upon the catecholamine which is employed to elicit the elevation in arterial blood pressure. The present data provide clear evidence that hypertension induced by NE and that induced by dopamine have distinctly different influences on the cerebrovasculature
Nemoto et al, 1996^66^	NE: 0.269 µg/min and 0.195 µg/min (n = 9) Donor Blood Transfusion: 5‐10 mL (n = 10)	5 to 10 mL dose	CBF: Hydrogen clearance technique CMRO_2_: Divisible into that associated with electroencephalographic	**PCO_2_ and PO_2_ were kept constant throughout all groups** **NE 38°C 0.269 µg/min:** CBF: 132 ± 27 mL/100 g/min CMRO_2_: 7.48 ± 2.49 mL/100 g/min **NE 34°C 0.195 µ g/min:** CBF: 121 ± 24 mL/100 g/min CMRO_2_: 5.41 ± 2.02 mL/100 g/min (*P* < .001) **Donor Blood 38°C:** CBF: 98 ± 28 mL/100 g/min (*P* < .05) CMRO_2_: 7.41 ± 1.78 mL/100 g/min **Donor Blood 34°C:** CBF:101 ± 32 mL/100 g/min CMRO_2_:6.31 ± 1.41 mL/100 g/min (*P *< .001)	None Mentioned	NE infusion during hypothermia could nullify the beneficial effects of mild hypothermia in cerebral protection NE slightly decreases CBF in both situations
Sato et al, 1987^67^	L‐threo‐3,4‐Dihydroxyphenylserine (L‐DOPS): 3 mg/kg and 1 mg/kg L‐DOPS and benserazide: 3 mg/kg and 3 mg/kg/hr L‐DOPS and propranolol: 3 mg/kg and 3 mg/kg/hr NE: 100 µg/kg/hr	3 mins	CBF: Hydrogen clearance method	**L‐DOPS 3 m/kg CBF:** Increase in striatal blood flow(SBF) **L‐DOPS 1 mg/kg CBF:** NS effect **L‐DOPS and benserazide:** CBF increase was inhibited by benserazide **L‐DOPS and propranolol:** CBF increase was inhibited by propranolol **NE CBF:** Marked increase to 40% at 20 mins then remained constant	None mentioned	The effects of L‐DOPS may be attributed to the action of NE formed from L‐DOPS, and the action may be mediated by stimulation of beta‐adrenoceptor NE increase CBF maybe due to cardiac output increase
Mascia et al, 1999^68^	NE: 0.08 mg/kg/min	30 mins × 2	rCBF: Hydrogen clearance technique PO_2_: Blood samples	**PCO_2_ and PO_2_ were constant throughout all groups** **NE:** CPP: Increased by 21 ± 2 (23 ± 2%) mmHg (*P* < .001) CBF: 3.6 ± 3.1 (6 ± 8%) mL/100 g/min (*P* = .5) **NE + endothelin‐1:** CPP: Increased by 18 ± 1 (20 ± 2%) mmHg (*P* < .001) CBF: 15.8 ± 4.1 (46 ± 13%) mL/100 g/min (*P* = .004) PO_2_: no significant change in any group	None mentioned	Endothelin‐1 production is required in the CBF response to increased CPP, but is not required in the maintenance of resting CBF. NE increased CBF to a higher amount in the endothelin‐1 group, indication its effect on cerebral response
Stromberg et al, 1992^69^	PD123319: 1‐10 mg/kg NE: 0.1‐3.2 µg/min	To increase hypertension 5 mins before PD was injected	CBF: Laser‐Doppler flowmetry	**NE:** CBF increased from 110 to a max of 160% (*P* < .01) **PD 1 mg/kg + NE:** increased from 90% to 150% (*P* < .01) **PD 10 mg/kg + NE:** remain relatively stable from 120% to 110% (*P* < .001)	None mentioned	PD did not alter baseline CBF at normal pressures, but appears to interfere with autoregulatory mechanisms of CBF. The participations of alpha‐2 receptors in the regulation of CBF confirms a physiological role for this receptor subtype and may give clues for future treatment of various cerebrovascular disorders NE increase CBF but maybe due to cardiac output then local ICP change
Zhang et al, 1991^70^	NE Increasing doses: 0.01‐30 µg/kg Superoxide dismutase: 24 000 units/kg plus 1600 units/kg/min	300‐400 g	CBF: Laser‐Doppler flowmetry PO2: Blood samples	**PCO_2_ and PO_2_ were kept constant throughout all groups** **NE 3 µg kg:** CBF: Increased by 300% (*P* < .03) **NE 10 µkg:** CBF: Slightly more than 3 µg/kg but not significantly(*P* < .03) **NE and Superoxide Dismutase:** CBF: Similar to NE as injection (*P* < .03)	Whereas five (63%) of the eight control rats died after the 10 µg/kg norepinephrine dose, all eight rats treated with superoxide dismutase survived this dose	Blood pressure and CBF responses to submaximal pressor doses of NE and reduces mortality associated with acute hypertension in rats
Gozzi et al, 2007^71^	NE: 0.125 µg/kg (n = 5) 0.5 µg/kg (n = 5) 2 µg/kg (n = 5) 8 µg/kg (n = 5) NE doses refer to the salt form of the compound	Over 80 s	MAP: MRI acquisitioner CBV: Laser‐Doppler flowmetry, and MRI	**PCO_2_ and PO_2_ remained constant throughout all groups** **NE 0.125 and 0.5 µg/kg:** rCBV: No significant changes were observed **NE 2 µg/kg:** rCBV: Short‐lived microvascular rCBV increases started to appear in some of the VOIs, focal areas of significant activation were apparent in the cingulate and retrosplenial cortices alongside the sagittal sinus **NE 8 µg/kg:** rCBV: Raised up to 15% (*P* < .01)	None mentioned	CBF autoregulation was maintained over a BP range of 60‐120 mmHg. Under these conditions, no significant central rCBV responses were observed, suggesting that microvascular rCBV changes in response to abrupt changes in perfusion pressure are negligible within the autoregulatory range. Larger BP responses were accompanied by significant changes in both CBV and CBF that might confound the interpretation of pharmacological MRI results. As the dose of NE was increased and MABP exceeded 130 mmHg. For MABP greater than 130 mmHg both LDF and microvascular rCBV showed transient but significant increases
Kuschinsky et al, 1983^72^	L‐NE: 1 mg/100 mL saline containing 0.1% ascorbic acid at 10‐100 µL/min (n = 4) 2 Deoxyglucose: 50 µCi/kg(n = 4) Iodo Antipyrine: 50 µCi/kg (n = 6)	Adjust to maintain stable heart rate	rCBF: Diffusible tracer with 14C amino antipyrine Local rates of cerebral glucose utilization (LCGU):Calculated from the local tissue concentrations	**PCO_2_ and PO_2_ were constant throughout all groups** rCBF during NE increased in most of the structures LCGU: −10% and + 74% (*P* < .05) in only 6 of 39 structures Despite this large variability, there was still a tight correlation between the rCBF	None mentioned	When compared to the relationship between LCGU and rCBF in a control group, the slope of the regression line was increased significantly by NE, indicating a resetting of the coupling mechanism. At a given metabolic rate, a higher blood flow is needed to perfuse a brain structure during NE infusion than during control conditions
Kraut et al, 2004^73^	NE 5 µg/100 g	60 sec	CBF: Laser‐Doppler flowmetry	**NE cerebral tissue blood Flow:** Increased by 270 ± 47% (*P* < .05)	None mentioned	The significant correlation between the hemodynamic state of the organs and its mitochondrial redox state may serve as an indicator of tissue vitality under "brain sparing" conditions NE was seen to increase CBF in almost all regions
Healthy lightly anesthetized animal models						
Artru et al, 1981^33^	E: 0.1 and 0.25 µg/kg/min NE: 0.25 µg/kg/min	40 mins injection 3 times with 20 mins rest	CBF: Determined by weighing timed collections and assuming the specific gravity of blood to be 1.05 CMRO_2_: Derived from measurements of arterial‐cerebral venous (sagittal sinus) blood oxygen content differences	**PCO_2_ and PO_2_ remained constant throughout all groups** **Cyclopropane Control:** CBF: 67 ± 7 mL/min/100 g CMRO_2_: 4.33 ± 0.49 mL/min/100 g **30‐40 mins with E 0.1 µg/kg:** CBF: 113 ± 17 mL/min/100 g (*P* < .05) CMRO_2_: 5.07 ± 0.57 mL/min/100 g (*P* < .05) **90‐100 mins with E 0.25 µg/kg:** CBF: 62 ± 12 mL/min/100 g CMRO_2_: 4.80 ± 0.66 mL/min/100 g (*P* < .05) **150‐160 mins with NE 0.25 µg/kg:** CBF: 63.0 ± 15 mL/min/100 g CMRO_2_: 5.32 ± 0.93 mL/min/100 g (*P* < .05) Overall increased CMRO_2_ by 17%‐23% within 10‐30 mins **Nitrous oxide, Halothane, Pentobarbital, or Ketamine:** Regardless of anesthetic, each infusion of E or NE resulted in an immediate increase in CBF which, except with E 0.1 µg/kg/min which returned to control levels within 10 mins No change in CMRO_2_ regardless of dose or duration of infusion	None mentioned	Cyclopropane but not the other anesthetics tested increased the permeability of the BBB and presumably allowed the passage of E or NE into the brain to increase CMRO_2_, reversibly. Opening of the BBB may be a direct effect of cyclopropane on endothelial cells or may be mediated by central adrenergic systems. For their part, E or NE may increase CMRO_2_, by either direct action on neuronal receptors or metabolically coupled synaptic events NE increase CMRO_2_ and CBF in all anesthetic methods tested
Lluch et al, 1973^39^	E: 0.1 to 5 µg (n = 10) NE: 0.1 to 5 µg (n = 10) Isoproterenol: 0.01 to 1 µg (n = 9) phenoxybenzamine: 200 to 400 µg propranolol: 250 µg	Until all gone	CBF: Radioactive gas elimination method CMRO_2_: Polyethylene Catheter	**PCO_2_ and PO_2_ were constant throughout all groups** **E CBF:** Decrease of 55 ± 3% **E and phenoxybenzamine CBF:** Decrease of 15 ± 4% **NE CBF:** Decrease 55 ± 3% **NE and phenoxybenzamine CBF:** Decrease 15 ± 5% **Isoproterenol CBF:** Increases increment of 75 ± 6% **Isoproterenol and propranolol CBF:** Increases increment of 12 ± 1%	None Mentioned	E, NE, and isoproterenol exert powerful direct effects on the cerebral circulation of the unanesthetized goat, and these effects appear to be mediated by alpha and beta receptors.
Perales et al, 1997^40^	NE: 10 µg/min 30 µg/min Magnesium sulfate (MgSO_4)_: Infused intravenously at 0.3 g and 3 g	15 mins	CBF: Electromagnetic flow probe MAP: Catheter in femoral artery CVR: Calculated as the mean arterial blood pressure in mmHg divided by CBF	**PCO_2_ and PO_2_ was not monitored** **NE 10 µg:** CBF: 55% CVR: 190% **MgSO_4_ 0.3 g and NE 10 µg:** CBF: Increase to 61% at 5 mins then constant (*P*<.01) CVR: Reduced to 178% at 5 mins (*P* < .01) **MgSO_4_ 3 g and NE 10 µg:** CBF: Increase to 80% at 5 mins then constant (*P* < .01) CVR: Reduced to 120% at 5 mins (*P* < .01) **NE 30 µg:** CBF: 80% CVR: 160% **MgSO_4_ 0.3 g and NE 30 µg:** CBF: Increase to 90% at 5 mins then constant (*P* < .01) CVR: Reduced to 140% at 10 mins (*P* < .01) **MgSO_4_ 3 g and NE 30 µg:** CBF: Increase to 110% at 10 mins then constant (*P* < .01) CVR: Reduced to 90% at 10 mins (*P* < .01) Contraction was on average 10% less in MgSO_4_ and NE then NE alone	None Mentioned	Magnesium sulfate reverses the NE‐induced cerebral vasoconstrictor and pressor responses by a direct inhibitory action of Mg2 + on the actions of NE in the cerebral and peripheral vascular beds, which leads to a decrease in vascular resistance.
Von Essen et al, 1972^43^	NE: 0.03 to 7.5 µg/kg/min 5‐HT: 0.1 to 22.8 µg/kg/min Dopamine: 0.05 to 57.4 µg/kg	Not Mentioned	CBF: Radioactive gas elimination method	**PCO_2_ and PO_2_ were not monitored** **NE:** CBF: Max reduction −21% (*P*=.01) CMRO_2_: Constant **5‐HT:** CBF: +28% (*P* < .01) CMRO_2_: Constant **Dopamine low dose:** CBF: −20% CMRO_2_: Constant Blocked with pimozide or haloperidol **Dopamine high dose:** CBF: +30% (*P* < .01) CMRO_2_: Constant Blocked by pimozide or haloperidol but not by propranolol	None Mentioned	Importance for the understanding of some circulatory disturbances of the brain and also for a correct interpretation of altered concentration of different amines, and their metabolites, in brain tissue and cerebrospinal fluid after administration of certain biogenic amines or their precursors.
Edvinsson et al, 1972^55^	Tyramine: 0‐10 mg/kg NE: 5 µg/kg	2 mins	CBV: Radioisotope dilution technique	**PCO_2_ and PO_2_ were kept constant throughout all groups** **Tyramine:** CBV: Decreased as dose increases with 12% at 0.1 mg/kg (*P* < .05) **NE under 12 hrs:** CBV: No significant change **NE over 24 hrs:** CBV: Reduced up to 33% (*P* < .01)	None Mentioned	That a NE induced vasoconstriction in the circulation of the brain depends on the quantitative access of the amine to the adrenergic receptor area. The vasoconstrictor response may be influenced by such features as the amount of adrenergic innervation, the types of adrenergic receptors present, and the properties of the barrier.
Animal models with ganglionectomy						
Alborch et al, 1977^41^	Tyramine: 50‐500 µg Norepinephrine: 0.03‐3 µg Phentolamine: 1 mg Propranolol:1 mg	1 mg in 1 mL of saline for 10‐15 mins	CBF: Electromagnetic flow transducer	**PCO_2_ and PO_2_ were not monitored** **Tyramine CBF:** Decreased versed control 50 µg: 10 to 1%CBF (control vs tyramine) 100 µg: 20 to 5%CBF 250 µg: 25 to 10% CBF 500 µg: 30 to 10%CBF **NE CBF:** Increased verse control 0.01 µg: 10 to 15%CBF (control vs NE) 1 µg: 15 to 25%CBF 2 µg: 25 to 45%CBF 3 µg: 39 to 54%CBF %CBF is the reduction percent of the CBF **Phentolamine:** CBF Before Removal: Increased by 31% CBF After Removal: Increased by 2%, **Propranolol:** CBF Before Removal: Reduced by 14% CBF After Removal: Reduced by 4%	None Mentioned	There is an active participation of the perivascular sympathetic nerve endings in the overall regulation of cerebrovascular resistance. The effects of phentolamine and propranolol on cerebral blood flow before and after removal of the superior cervical sympathetic ganglion indicate that under normal conditions both alpha and beta receptors display a tonic adrenergic activity in the cerebral blood vessels. NE decrease CBF in all doses with increase dose causing increased response
Aubineau et al, 1985^60^	NE: 1.8 to 2.2 µg/kg/min Angiotensin II (AT): 1.0 to 1.8 ≥g/kg/min	30 s	CBF: Radioactive microsphere with helium and thermal clearance PO_2_: Measure with probes samples	**PCO_2_ was kept constant throughout all groups** **NE:** CBF: Not significantly changed PO_2_: Reduce by 9% (*P* < .05) **AT:** CBF: Reduced by 10% PO_2_: Reduced By 9% (*P* < .001) **Stim:** CBF: Decrease 23.6 in heterolateral hemisphere and 22.2 mL/100 g/min in homolateral PO_2_: Reduced by 18% (*P* < .01)	None Mentioned	As in the peripheral circulation, chronic sympathectomy affects the equilibrium of the vascular smooth muscle fibers but that circulating amines play no compensatory role in the cerebral circulation because of the blood‐brain barrier. NE did not significantly change CBF
Animal models with bile duct removed						
Bloom et al, 1975^19^	NE: 8, 16, and 32 µg/min	10 min	CBF: Xenon clearance method Cerebrovascular Resistance (CVR): Calculated with pressure/flow	**PCO_2_ and PO_2_ were constant throughout all groups** **NE 8 µg:** CBF: Reduction 8.4 ± 4.3 mL/100 g/min (*P* < .005) CVR: Decrease 0.21 ± 0.12 mmHg/mL/min **NE 8 µg and Jaundice:** CBF: Reduction 9.48 ± 2.63 mL/100 g/min (*P* < .005) CVR: Decrease 0.66±0.28 mmHg/mL/min **NE 16 µg:** CBF: Reduction 8.6 ± 6 mL/100 g/min (*P* < .02) CVR: Increase 0.001 ± 0.11 mmHg/mL/min **NE 16 µg and Jaundice:** CBF: Reduction 10.9 ± 4.4 mL/100 g/min (*P* < .02) CVR: Increase 0.9 ± 5.56 mmHg/mL/min **NE 32 µg:** CBF: Reduction 1.97 ± 4.6 mL/100 g/min CVR: Increase 0.425 ± 0.17 mmHg/mL/min **NE 32 µg and Jaundice:** CBF: Reduction 5.16 ± 3.6 mL/100 g/min CVR: Increase 0.71 ± 0.28 mmHg/mL/min	None Mentioned	Indicate that in baboons following ligation of the bile duct there is an altered cerebrovascular response to infused NE. Cerebral vasoconstriction was obtained with infusions of NE at 8 µg and 16 µg in the jaundiced animals, whereas dilatation was evident in the control animals. These findings suggest an increased cerebrovascular sensitivity to NE in the obstructive jaundice following bile duct ligation.
Healthy heavily anesthetized animal models with craniotomy						
Shalit et al, 1974^24^	ICP balloon increase (n = 18) Brain swelling (n = 8) NE drip was increased to make 40 to 80 mmHg blood pressure	10 to 15 min	rCBF: Krypton clearance method ICP: Epidural transducer PO_2_: Measured with electrode system	**PCO_2_ and PO_2_ were constant throughout all groups** **Balloon increase:** NE did not significantly affect ICP below if ICP was below 70 mmHg but does above NE results in a significant spike increases for rCBF (0.7 mL/gm/min) at each dose, with less effect result at ICP above 80 mmHg **Brain Swelling:** NE did not significantly affect ICP below 80 mmHg but does above NE did not significantly affect the CBF	None Mentioned	An increase in blood pressure in intracranial hypertension is not a favorable compensatory mechanism designed to maintain brain function. NE had no significant results of rCBF but in high ICP NE injection did increase CBF
Ulrich et al, 1985^25^	Phenylephrine: 10^−9^ to 10^−3^ mol/L (n = 19) Oxymetazoline: 10^−9^ to 10^−3^ mol/L (n = 21) Prazosin: 10^−8^ to 10^−4^ (n = 15) Yohimbine: 10^−8^ to 10^−4^ (n = 23) NE: 10^−7^ to 10^−4^ mol/L(n = 25)	Injection of full solution	Venous diameter (VD): Glass micropipette with sharpened tips were filled with the test solutions and mounted on a micromanipulator	**Phenylephrine VD:** 1 to −10% at 10^−3^ **Oxymetazoline VD:** 2 to −8% at 10^−5^ then slightly increased **Prazosin VD:** Venous diameter remains constant **Prazosin and NE VD:** −15% to 0 **Yohimbine VD:** −15% to 0 **Yohimbine + NE VD:** −23 to −1%	None Mentioned	Since both alpha and alpha‐2 adrenoceptor agonists are less potent constrictors of pial veins than NE in vivo, a preferential use of alpha, or alpha‐2 adrenoceptor agonists cannot be recommended, if a therapeutic reduction of ICP or blood volume is desired.
Wei et al, 1975^26^	NE: 0, 10, 20 and 100 µg/mL Concentration of CSF was calcium increase 10 mEq/l	Short and long periods of time	CBF: Free diffusible tracer technique Bulb placed for sampling and ABP	**NE 0 µg**/**mL Small vessel diameter(µm):** 44.4 ± 1.8 **NE 10 µg**/**mL:** 43.3 ± 1.9 **NE 100 µg**/**mL:** 44.3 ± 1.4 **Ca^2+^ and CSF:** NE 100 µg/mL cased the only change in diameter from 43.3 ± 2.1 to 42.9 ± 2.4 µm **Ca^2+^ and Mg^2+^ in CSF:** **NE 0 µg**/**mL Small vessel diameter(µm):** 49.8 ± 2.3 **NE 10 µg**/**mL:** 47.5 ± 3.1 **NE 100 µg**/**mL:** 47.0 ± 4.6 **Wahl solution:** **NE 0 µg**/**mL Small vessel diameter(µm):** 49.8 ± 2.3 **NE 10 µg**/**mL:** 48.2 ± 2.2 **NE 100 µg**/**mL:** 47.5 ± 2.0 For all Ca^2+^ levels and Mg^2+^ levels and Wahl solution all small pail arties changes similar with changes in NE	None Mentioned	The results imply a functional role for postganglionic autonomic fibers in CBF autoregulation. NE in high concentration is capable of producing substantially greater constriction of these vessels than by sympathetic nerve stimulation suggests that the potential exists for NE‐induced reductions in CBF of considerable magnitude under abnormal conditions, such as in response to brain injury.
Busija et al, 1987^44^	NE: 10^−6^ to 10^−4^ mol/L (n = 18) Isoproterenol: 10^−8^ to 10^−6^ mol/L (n = 7)	5 mins	Pial arteries were observed with a wild trinocular stereo microscope. Pial arterial diameter was measured with a television camera mounted on the microscope, a video monitor, and a video microscale	**PCO_2_ and PO_2_ remained constant throughout all groups** **NE:** Constricted pial arteries: 203 ± 27 µm to 164 ± 18 µm (20 ± 2%) (n = 21 vessels from 16 animals) at 10^−4^ mol/L . Concentration in CSF of 6‐keto‐prostaglandin B to increase from 768 ± 91 to 1544 ± 151 pg/mL, thromboxane B2 to increase from 188 ± 37 to 269 ± 38 pg/mL, and prostaglandin E2 to increase from 2067 ± 448 to 6575 ± 751 pg/mL **Isoproterenol:** Did not affect pial arterial diameter at 10^−5^ mol/L, but dilated pial arteries by 28 ± 3% at 10^−7^ mol/L and 32 ± 2% at 10^−6^ mol/L. At the same time, CSF levels of 6‐keto‐PGFaa, TXB2, and PGE2 did not change.	None Mentioned	NE elicits the release of prostanoids from the cortical surface, and that these substances limit cerebrovascular constriction to NE. That sympathetic nerve stimulation and exogenous NE are able to have substantial constrictor effects on the cerebral circulation of newborn pigs, and our findings are consistent with an important role of the sympathetic nervous system in regulation of CBF in the newborn animal.
Leffler et al, 1989^45^	NE: 10^−6^ to 10^−4^ mol/L In three groups Sham‐operated control(n = 7), 2‐3 hrs postischemia (n = 6) and 24 hrs postischemia(n = 6)	20 mins	Catheters placed in aortae for blood withdrawal and monitoring Prelims experiments showed that blood pressure was reduced such that radiolabeled microspheres did not work Observe pial arterioles with trinocular stereo microscope	**PCO_2_ and PO_2_ was kept constant throughout all groups** **NE 10^−4^ mol/L:** Decreased pial arteriolar diameters similarly in all three groups (27%, 28%, and 21%) **Sham‐operated group:** Hypotension increased cortical subarachnoid cerebrospinal fluid prostanoid concentrations Exhibited pial arteriolar dilation in response to hypotension (28% at 33 mmHg ) **2‐3 and 24‐hrs group:** Hypotension decreased pial arteriolar diameters (21% and 17%, respectively). No alteration to cerebral prostanoid	None Mentioned	After cerebral ischemia, autoregulatory pial arteriolar dilation in response to hypotension is absent, while vasoconstriction in response to NE is intact.
Myburgh et al, 1998^54^	Dopamine: 0‐60 µg/kg/min E: 10,20,40,60 µg/kg/min NE: 10,20,40,60 µg/kg/min	5 mins	CBF: Ultrasonic‐Doppler transducer ICP: Intraparenchymal strain gauge catheter Cerebral oxygen utilization COU: Signa CBF an auto‐venous oxygen content difference	**PCO_2_ and PO_2_ were assumed to be constant throughout all groups** **Dopamine:** ICP: Significant increase on does greater than 20 µg/kg/min (78.6 ± 13.1 to 97.2 ± 8.8% CBF: Statistically significant rise in CBF after 40 µg/kg/min (13.2 ± 3.2 to 52.6 ± 24.3%) COU: Initial decrease at 20 µg/kg/min followed by increase to base line at 60 µg/kg/min **E:** ICP: Dose‐dependent increase after 40 µg/min CBF: No significant change COU: No significant change **NE:** ICP: Did not increase CBF: No significant change COU: No significant change	None Mentioned	Intact cerebral autoregulation model‐induced hypertension by E and NE is not associated which changes in CBF, where dopamine causes cerebral hyperemia increased ICP and increased global cerebral oxygen utilization
Muir et al, 1993^74^	Ten mins after cocaine (1 mg/kg, iv) or saline: NE: increasing from 0.01‐10 µg/kg	The pressor effect of L‐NMMA was controlled for by comparison with NE titrated to effect an equivalent blood pressure elevation	Cortical blood flow (CoBF): Laser‐Doppler flowmetry	**PCO_2_ and PO_2_ were kept constant throughout all groups** Cocaine significantly potentiated the blood pressure and cerebral blood flow responses **NE:** CoBF: Increased at 10^−4^ µg/kg to 40% and 10^−1^ µg/kg to 150%	None Mentioned	Cocaine causes a rapid, transient increase in blood pressure and CBF and potentiates the magnitude and duration of the pressure and flow response to NE. Repetitive blood pressure elevations in cocaine abusers is one of the proposed mechanisms leading to damage of cerebral vessels
Healthy heavily anesthetized animal models with explanted brains						
Oberdorster et al, 1973^34^	E: 0.001‐10 µg (n = 5) NE: 0.001‐10 µg (n = 5) Isoprenaline: 0.001‐10 µg (n = 5)	30 sec	CBF: Photoelectric drop recorder CVR: Calculated with CBF and internal perfusion pressure ICP: Isolated with two pressure transducers	**PCO_2_ and PO_2_ remained constant throughout all groups** **E and NE:** Dose‐dependent increase of CVR ranging from 2% to 61% CVR: NE could be reversed by phentolamine, E were increased by propranolol CBF decreases linearly with inject from 0 to −5 mL/100 g/min **Isoprenaline:** Dose‐dependent decrease of CVR ranging from −5% to ‐ 51% CVR effect could be prevented by propranolol CBF Increase to 12 mL/100 g/min at 1 µg then remain relatively stable The dilator potency was as follows: Isoprenaline: Epinephrine: Norepinephrine = 1:0.5:0.3 The constrictor potency was as follows: Epinephrine: Norepinephrine: Isoprenaline = 1:0.5:0	None Mentioned	These sources of contamination cannot account for the vasomotor responses and that, consequently, both alpha and beta‐adrenergic activity of the cerebral vessels of the dog has been demonstrated. NE increase CVR and decrease CBF which can be mediated with phentolamine
Lowe et al, 1971^35^	Phenylephrine: 50‐200 µg Isoproterenol: 15‐40 µg NE: 15‐100 µg E: 15‐100 µg	Until dose gone	CBF: Maintained with pump Pulsatile perfusion pressure: Recorded with servo channel of a Gilson five‐channel polygraph CVR: Calculated by mean perfusion pressure/CBF	**PCO_2_ and PO_2_ remained constant throughout all groups** **Phenylephrine:** CVR: Increased over each dose increase **Phenylephrine and Phenoxybenzamine:** CVR: Less effective **Isoproterenol:** CVR: Decreased, no apparent correlation to dose **Isoproterenol and propranolol:** CVR: Reduced effectiveness **NE:** CVR: Increased with no apparent correlation to dose **NE and phenoxybenzamine:** CVR: Reduced response **E:** CVR: Increased with no apparent correlation to dose **E and phenoxybenzamine**: CVR: Decreased **E and propranolol:** CVR: Increased		As catecholamine blood levels in intact dogs are low in comparison to those achieved in these studies, it appears doubtful that circulating catecholamines play an important physiological role in the regulation of CVR. Possible explanations are considered for the lower response of the cerebral vasculature to catecholamines when this response is compared to that observed in other vascular beds
Zimmer et al, 1974^36^	NE: 2 µg/min E: 2 µg/min Isoprenaline: 0.2 µg/min	10 mins	CBF: Photoelectric drop recorder CVR: Calculated on pressure flow relationship CMRO_2_: Changes in oxygenation in blood samples	**PCO_2_ and PO_2_ remained constant throughout all groups** **In all groups the CVR and CBF effects are taken after indirect effects of drug are removed** **NE:** CBF: Decreased by 0.2 ± 6.0% (*P* > .05) CVR: Reduced by 50% CMRO_2_: Not changed **E:** CBF: Decreased 4.1 ± 3.3% CVR: Reduced by 50% CMRO_2_: Not changed **Isoprenaline:** CBF: Increased by 9.3 ± 3.6% CVR: Reduced by 50% CMRO_2_: Not changed Note max CBF change was found within 1.5‐2 mins and persisted to the end of infusion	None Mentioned	Based on these investigations it is assumed that no pronounced vascular adjustments occur in the cerebral circulation during catecholamine infusions; however, CBF is significantly affected by catecholamine.
Omar et al, 2010^75^	NE: 2.5 µg/kg Nitro‐L‐arginine methyl ester (L‐Name): 10 mg/kg	To maintain ABP to 180 mmHg in mature and middle‐aged 150 mmHg in juveniles rat	Carotid blood flow (CoBF) and MABP: Transonic flow probe	**PCO_2_ and PO_2_ were kept constant throughout all groups** For all groups CoBF decreased after the injection of NE with a decrease of 0.5 mL/min (*P* < .05) in mature and 0.5 mL/min (*P* < .01) in middle age the juvenile only has a minor drop and it was not significant Carotid vascular conductance (CVC) in all was significant at 0.005 mL/min (*P* < .01) juvenile and 0.08 mL/min (*P* < .001) for mature and middle age rats **L‐Name + NE:** CoBF for juvenile and mature there was a slight decrease; in middle age there was a small increase CVC for juvenile and mature there was a slight decrease; in middle age there was a small increase	None Mentioned	The results of these two studies indicate that by middle age, agiing itself has already altered several key mechanisms that regulate the carotid circulation that includes the brain
Takahashi et al, 2000^76^	NE:10^−7^ to 10^−5^ mol/L Yohinbin: 10^−6^ mol/L Prazosin:10^−8^ mol/L 5‐HT: 10^−10^, 10^−8^, 10^−6^ mol/L Ketanserin: 10^−6^ mol/L Methiothepin: 10^−6^ mol/L	5 mins	Contractile diameter: Glass pipettes on micromanipulators monitored with video camera	**NE:** As dose increases contractile diameter increases **Yohinbin + NE:** Significantly decrease control change(n = 5, *P* < .05) **Prazosin + NE:** Slight decrease in contractile change (n = 5) **5‐HT:** Increase in control response with dose increase **Ketanserin + 5‐HT:** Significantly dropped in contractile response (n = 5 *P* < .05) **Methiothepin + 5‐HT:** Slight decrease in contractile response	None Mentioned	That 5‐HT plays a significant role in arteriolar contractility only from the CSF side, while NE is an important regulator or regulator of arteriolar contractility from both the CSF and blood circulation sides. NE causes dose‐dependent contractions of arterioles
Various animal models						
Mori et al, 1999^27^	Group A Hypothermia: (n = 10) Group B Hypothermia with NE: 6‐30 µg/kg (n = 6) Group C Hypothermia with Barbiturate (thiopental): 5 mg/kg (n = 6)	Increase Blood Pressure to 25 mmHg	CBF: Hydrogen clearance method CMRO_2_: Calculated with arteriovenous oxygen difference and cerebral venous oxygen saturation taken from the superior sagittal CVR: Calculated from (MABP ‐ ICP)/CBF CBV: Technetium‐99 m‐labeled human serum albumin in 12 Ca	**PCO_2_ and PO_2_ were kept constant throughout all groups** **Group A:** CBF: 51.2 ± 8.3 mL/100 g/min at 37°C and decreased with lower brain temperature (6.1 ± 2.7 at 25°C) CMRO_2_: 2.24 ± 0.75 mL/100 g/min at 37°C was also decreased by 0.52 ± 0.20 at 25°C CBV: 5.3 ± 1.2% at 37 C decreased significantly at 29°C 3.7 ± 1.0% (*P* < .05) CVR: 3.2 ± 0.7 mmHg*mL/100 g/min at 37°C increased significantly at 29°C 13.8 ± 5.2 (*P* < .01) **Group B:** CBF: 24.2 ± 3.7 mL blood/mL O_2_ 24.6 ± 7.4 at 33°C 19.1 ± 4.3 at 25°C CMRO_2_: Proportional change associated with CBF **Group C:** CBF/CMRO_2_: Did not decrease	None Mentioned	These results suggest that hypothermia may cause vasoconstriction and misery perfusion in the brain. This potential risk of relative ischemia can be avoided by combination with vasopressor administration, that cerebral hypothermia may cause cerebral vasoconstriction and relative ischemia. To avoid this misery perfusion, patients should not be cooled below 31°C. Hypothermia combined with vasopressor administration may avoid this serious cerebral metabolic disturbance.
Panther et al, 1985^37^	Adenosine: 4.94 µmol/L per kg NE:0.7 µg/kg/min	Not mentioned	CBF: Radioactive microspheres PO_2_: Blood samples	**PCO_2_ and PO_2_ were constant throughout all groups** **Control:** Cerebrum CBF: 58 mL/min/100 g Tumor CBF: 1 mL/min/100 g PO_2_: 105 mmHg **Adenosine:** Cerebrum CBF: 10 mL/min/100 g Tumor CBF: 100 mL/min/100 g PO_2_: 121 mmHg **NE:** Cerebrum CBF: −1 mL/min/100 g Tumor CBF: −23 mL/min/100 g PO_2_: 93 mmHg	None Mentioned	Selective effects of adenosine and NE on blood flow to brain tumors may have important implications for chemotherapeutic treatment of brain tumors. Vasodilator drugs such as adenosine that selectively increase tumor blood flow, but not brain blood flow and may increase the therapeutic advantage of lipid soluble chemotherapeutic drugs.
Nakagawa et al, 1977^38^	NE: 5 µg/kg	1.5‐3 mins	ICP: Pressure transducers PO_2_: Blood samples taken	**PCO_2_ was kept constant throughout all groups** All values in mmHg **NE:** Control: 125.0 ± 6.4 After needle insertion: 139.2 ± 7.1 After first coagulation: 167.7 ± 12.7(*P* < .01) After second coagulation: 133.8 ± 9.8 **NE and Lesion:** Control: 396.0 ± 25.4 After needle insertion: 346.0 ± 9.2 NS After first coagulation: 362.0 ± 17.5 NS After second coagulation: 342.2 ± 20.8 NS PO_2_ remains steady throughout the experiments	None Mentioned	NE was not significant regardless of the level of the ICP, or of uni‐ or bilateral lesions of the hypothalamus. NE resulting no significant change to CBF found from the ICP/PO_2_ relationship
Miller et al, 1984^46^	NE: Not specified (n = 6) Dopamine: Not specified (n = 5) Phenylephrine: Not specified (n = 6)	Endotoxin induced by bacteria for 40 min in Dose to raise MABP to 70‐80 mmHg	CBF: Radiolabeled microsphere technique	**PCO_2_ and PO_2_ were kept constant throughout all groups** **NE, Dopamine, Phenylephrine:** Affected CBF similarly in all brain regions, with a decrease in brain total, cortex close to 27.1 ± 2.8 and 26.3 ± 28 mL/min/100 g where is the cerebellum slight decrease at 40.9 ± 4.9. The brain stem increased by 41.8 ± 4.7 mL/min/100 g (*P* < .05) for all but compared to shock for last two. **Control to Shock:** Brain: 37.8 ± 2.9 to 25.2 ± 3.1 Cortex: 36.1 ± 2.7 to 22.9 ± 2.8 Cerebellum: 47 ± 3.6 to 30 ± 8.4 Brainstem: 35.9 ± 3.1 to 24.3 ± 2.6	Cerebellum and brainstem did not restore to control values with dose which may indicate underlying structural heterogeneity	Decreases in regional CBF with shock are similar to those reported by other, unchanged cortical CBF after injection suggest either an inability to autoregulate or disruption of the brain‐blood barrier resulting in vasopressor induced vasoconstriction which limits flow.
Anesthetized animal models given CPR						
Prengel et al, 2005^47^	E: 200 µg/kg Vasopressin: 0.4 units/kg NE + E + Vasopressin: 45 µg/kg, 45 µg/kg and 0.4 units/kg	Up to 5 mins	Organ perfusion: Radiolabeled microspheres technique	**PCO_2_ and PO_2_ were kept constant throughout all groups** **CBF:(mL/min/100 g)** **Before, 90 sec and 5 min after drug administration** **E:** 8 ± 2, 23 ± 3, and 17 ± 3 **Vasopressin:** 11 ± 3, 55 ± 7, and 52 ± 7 **NE + E + Vasopressin:** 4, 67 ± 13, and 53 ± 12 (*P* < .05 at 90 sec and 5 mins vasopressin vs E and vasopressin/E/NE vs E). CPP: Increased significantly after 90 sec in all drug administrations, with a decrease in E and NE + E + Vasopressin group after 5 mins , vasopressin increased slightly after 5 mins Two of seven animals in the epinephrine group, four of seven animals in the vasopressin/epinephrine/ norepinephrine group, and seven of seven animals in the vasopressin group could be successfully resuscitated	None Mentioned	Vasopressin with or without E and NE resulted in higher myocardial and cerebral perfusion than E alone, but there was no benefit in adding NE to vasopressin and E with regard to cardiac and CBF during cardiopulmonary resuscitation.
Hoekstra et al 1990^48^	E: 0.2 mg/kg(n = 7) NE: 0.20 mg/kg(n = 7) 0.08 mg/kg 0.12 mg/kg 0.16 mg/kg 0.2 mg/kg	3.5 mins	CBF: Radiolabeled microsphere technique	**PCO_2_ and PO_2_ were kept constant throughout all groups** **During normal sinus rhythm:** CBF: No significant differences (*P* ≥ 0.13) **During CPR, LCBF, and CBF:** NS differences (*P* ≥ 0.3) **NE 0.2 mg and E 0.2 mg:** CBF: NE as higher by 0.2 mg/kg but NS (*P* ≥ 0.23) **NE 0.08 mg/kg CBF:** 3.7 ± 3 mL/min/100 g (P = NS) **NE 0.12 mg/kg CBF:**13.5 ± 1.4 mL/min/100 g (P = NS) **NE 0.16 mg/kg CBF:** 23.7 ± 24.5 mL/min/100 g (P = NS) **NE 0.2 mg/kg CBF:** 16.8 ± 14.6 mL/min/100 g (P = NS) **All drug administration:** CBF, MBF, MDo, and MVo, rose while ER fell in both E and NE with no significant differences between groups in CBF, ER, or intravascular pressures following drug administration (*P* < .07). **NE:** CBF: As dose increases there was an increase in CBF that stopped and went down after 0.16 mg/kg, found in all brain areas CPP: Significant increase at 0.12 mg/kg then an average decrease with increasing dose (*P* < .05)	None Mentioned	NE 0.20 mg/kg is as effective as E 0.20 mg/kg at improving myocardial and CBF during CPR. NE 0.20 mg/kg improves MBF and MDo, over E 0.20 mg/kg, but any theoretical benefits of higher MBF and MDo, are offset by a proportional increase in MVo, in the NE‐treated animals. Dose lower than 02.mh/kg are probably more effective in the treatment of prolonged cardiac arrest.
Brown et al, 1989^49^	E: 0.20 mg/kg (n = 5) NE: 0.08 mg/kg (n = 5) NE: 0.12 mg/kg (n = 5) NE: 0.16 mg/kg (n = 5)	30 sec	CBF: Radiolabeled microsphere technique	**PCO_2_ and PO_2_ were kept constant throughout all groups** **E:** rCBF: not statistically significant but superior then low doses of NE but medulla and cervical cord improved significantly **NE 0.08 mg/kg (N = 5);** Slightly increased vasoconstrictor effect **NE 0.12 mg/kg and 0.16 mg/kg:** Increased regional cortical CBF by 12 mL/min/100 g No significant increase to left cerebral cortex **NE 0.16 mg/kg:** Increased regional cortical CBF on the average above 23 mL/min/100 g	None Mentioned	No significant difference in rCBF between the two highest doses of NE and E, 0.20 mg/kg, but these doses were superior to NE, 0.06 mg/kg, for improving flew to lower brainstem structures. That following a prolonged cardiac arrest, large doses of NE significantly improve CBF above that measured during CPR. Adrenergic agonists that contains A and B1 agonists but lacks B2 agonist properties may prove beneficial in this setting.
Lindner et al, 1990^50^	NE: 45 µg/kg E: 45 µg/kg	90 sec and 5 mins	CBF: Radiolabeled microsphere technique Cerebral Venous Blood and measure sagittal pressure: Catheter	**PCO_2_ and PO_2_ were kept constant throughout all groups** **E (open chest CPR, 90 sec and 5 mins):** CBF: 30 ± 7 to 54 ± 14 to 37 ± 17 mL/min/100 g (*P* < .05) Cerebral oxygen delivery: 4.3 ± 1.2 to 7.4 ± 1.7 to 5.1 ± 2.4 mL/min/100 g (*P* < .05) Cerebral Perfusion Gradient: 2.7 ± 0.5 to 4.4 ± 1.5 (*P* < .05) to 3.3 ± 1.2 kPa **NE (open chest CPR, 90 sec and 5 mins):** CBF: 30 ± 11 to 58 ± 22 to 45 ± 21 mL/min/100 g (*P* < .05) Cerebral oxygen delivery: 3.7 ± 1.4 to 7.3 ± 2.7 to 5.8 ± 2.7* mL/min/100 g (*P* < .05) Cerebral Perfusion Gradient: 2.5 ± 0.8 to 4.3 ± 1.2 to 3.9 ± 0.5 kPa (*P* < .05)	None Mentioned	NE and E after a 5‐min cardiac arrest and 3 mins of open‐chest CPR led to the same increase in cerebral oxygen delivery more than cerebral oxygen consumption, and oxygen extraction decreased. Both are strong alpha and beta‐receptor stimulators, but in contrast to E, the beta effect of NE is weak. NE demonstrated an increase in CBF and CMRO_2_
TBI anesthetized animal models						
Armstead et al, 2016^51^	Fluid percussion injury (FPI) post‐treated with NE 0.7‐1.3 µg /kg/min FPI post‐treated with NE 0.7‐1.3 µg /kg/min + the ERK MAPK antagonist U 0126 1 mg/kg intravenously Papaverine: 10^−8^ and 10^−6^ mol/L	CPP was targeted 65‐70 mmHg	CBF: Radiolabeled microsphere technique CPP: MAP ‐ ICP ICP: Integra camino monitor and laser‐Doppler probe Transient hyperemic response ratio (THRR): Calculated by flow before compression/release of compression	**PCO_2_ and PO_2_ was kept constant throughout all groups** **Sham control:** CPP male: 70 ± 7 mmHg CPP female: 71 ± 7 mmHg CBF male: No change CBF female: No change THRR male unilateral and bilateral: 1.15 and 1.27 THRR female unilateral and bilateral: 1.15 and 1.25 **FPI untreated:** CPP male: 45 ± 4 mmHg CPP female: 45 ± 5 mmHg CBF male: Reduced by 20 mL/min/100 g (*P* < .05) CBF female: Reduced by 15 mL/min/100 g (*P* < .05) THRR male unilateral and bilateral: 1.04 and 1.10 THRR female unilateral and bilateral: 1.07 and 1.14 **FPI post‐treated with NE:** CPP males: 68 ± 5 mmHg CPP females: 66 ± 5 mmHg CBF male: Reduced by 10 mL/min/100 g (*P* < .05) CBF female: No change (*P* < .05) THRR male unilateral and bilateral: 1.14 and 1.21 THRR female unilateral and bilateral: 1.15 and 1.25 **FPI post‐treated with NE + the ERK MAPK:** CPP males: 67 ± 5 mmHg CBF male: No change (*P* < .05) THRR male unilateral and bilateral: 1.15 and 1.25 No female data Papaverine increases artery diameter in all groups	None Mentioned	NE protects cerebral autoregulation and limits hippocampal neuronal cell necrosis after FPI in both male and female juvenile pigs. In contrast, NE augmented ERK MAPK upregulation in newborn males but similarly blocked it in newborn females after TBI. NE reduced CBF in male pigs with an increase in CVR in both sexes
Friess et al, 2012^52^	NE and PE: 7.9 ± 5.2 and 0.9 ± 0.7 µg/kg/min titrated to CPP > 70 mmHg	For 5 hrs	CBF: Thermal diffusion probe ICP: Intraparenchymal monitors PO_2_: Microdialysis	**PCO_2_ and PO_2_ remained constant throughout all groups** **PE:** CBF: Improves over time with peaks and valleys ranging 20 mL/100 g/min CPP: No significant change Greater reduction in cell injury **NE:** CBF: Improves over time with peaks and valleys ranging 20 mL/100 g/min CPP: No significant change PO_2_: Higher than PE No ICP difference between groups at 70 mmHg	None Mentioned	NE resulted in greater increase in brain tissue oxygen tension than augmentation with PE, despite similar increases in CBF
Daley et al 2004^53^	NE: 1 µg/kg/min	5 mins	CBF: Laser‐Doppler flow meter velocity Pail arteriolar: VHS recordings ICP: Direct pressure monitor and femoral ABP recordings CPP: ABP‐ICP HMF: Calculated from transfer from ABP to ICP	**PCO_2_ and PO_2_ were kept constant throughout all groups** **Uninjured and NE:** An inverse relationship between HMF and CPP with a mean of 0.50 ± 0.14 and 0.6 ± 0.44 Hz/mmHg CBF velocity: Decrease that remained relatively constant **Injured and NE:** Direct relationship between HMF and CPP with a mean 0.48 ± 0.21 and 1.13 ± 2.08 Hz/mmHg CBF: Increased after injury **FPI:** CBF: −3.64 ± 12% ICP: 61 ± 32% ABP: 43 ± 24% **After FPI:** CBF: 8.47 ± 20% ICP: 44 ± 28% ABP: 58 ± 26%	None Mentioned	Relating changes in HMF to changes in CPP may be of even greater value for evaluating the state of cerebrovascular regulation than evaluating changes in mean ICP induced by pressor challenge alone. However, the conclusions of this is only known to be applicable to a hypertensive challenge with NE under conditions of FPI obtained from an animal model with characteristics of diffuse axonal injury, and it might not apply to other situations or pathologies. NE appeared to increase CBF after TBI but limited effect in healthy models
Ract et al, 2001^77^	Dopamine: 5 mg/mL (average: 274 ± 110 µg/kg/min) NE: 0.1‐0.2 mg/mL (average: 18 ± 4.5 µg/kg/min)	Started at 0.1 mL/h and increased 0.1 mL/h until CPP above 70 mmHg	CBF: Extradural laser‐Doppler fiber ICP: Intraparenchymal fiber‐optic device	**PCO_2_ and PO_2_ remained constant throughout all groups** **Head trauma:** ICP: Remained constant at 27 ± 18.5 mmHg CPP: Remained constant 28 ± 22 mmHg CBF Decreases significantly from time 60 to 180 mins **NE:** ICP: Increased to 40 mmHg at 30 mins then dropped slightly (*P* < .05) CPP: Decreased over time after 15 minss to 10 mmHg (*P* < .05) CBF: Decreased significantly similar to all other groups **Dopamine:** ICP: Increased to 50 mmHg at 45 mins then stayed constant (*P* < .05) CPP: No change CBF: Decreased significantly similar to all other groups	None Mentioned	NE and dopamine are not able to restore values of CPP above 70 mmHg in a model of severe brain trauma and their systemic vasopressor properties are altered. NE indicates no change to CBF
Review article						
Kovach et al, 1976^16^	Various studies		CBF: Measured with a variety of methods including autoradiograph 14C, radioactive microsphere with Xenon clearance	Microinjection of NE into the hypothalamus of the rabbit caused increased flow at low concentrations and decreased flow at higher concentrations. One study observed marked CBF reduction after NE injection in hypercapnia. Three studies resulted in no CBF increase in the baboon in hemorrhagic shock upon administration of 6% CO_2_. In cross‐circulation experiments in which the brain of the recipient dog was hemodynamically isolated from the trunk and perfused by a donor dog, intravenous E or NE injection into the recipient's trunk caused reflexly a significant increase in its total CBF. Intracarotid injection of both catecholamines produced a significant fall in CBF. Increased CBF could be measured during intravenous infusion of NE in hemorrhagic shock, while the cerebrovascular resistance showed no change. Increased CBF accompanied by increased cerebrovascular resistance followed NE administration during tourniquet shock	None mentioned	The reviewed results clearly suggest that vital functions of the brain in spite of the well‐developed autoregulatory mechanisms are impaired during long‐lasting hypovolemic and other shock conditions. The insufficiency of the cerebrocortical and hypothalamic regulatory mechanisms can contribute to the development of the irreversible shock. In other words, failure of the body suffering from shock to restore the homeostatic equilibrium can be attributed to the inadequacy of the central nervous servo control system

Abbreviations: ABP, arterial blood pressure; AT, Angiotensin II; CBF, cerebral blood flow; CBV, cerebral blood volume; ChBF, choroidal blood flow; CMOT, Catechol‐*O*‐methyltransferase; CMR_glc_, cerebral glucose uptake; CMRO_2_, cerebral oxygen consumption; CoBF, corticoid blood flow; COU, cerebral oxygen utilization; CO_2_, carbon dioxide; CP, cerebral profusion; CPR, cardiopulmonary resuscitation; CPP, cerebral perfusion pressure; CSF, cerebral spinal fluid; CVR, cerebrovascular resistance; E, epinephrine; ERK, extracellular signal‐regulated kinase; FPI, fluid percussion injury; HMF, highest modal frequency; hrs, hours; ICP, intracranial pressure; IL‐6, interleukin‐6; keto‐PGFaa, 6‐keto‐prostaglandin; L‐DOPS, l‐threo‐3,4‐dihydroxyphenylserine; L‐Name, Nitro‐L‐arginine methyl ester; L‐NMMA, methylarginine; MABP, mean atrial blood pressure;; MAC, minimum alveolar concentration; MAO, Monoamine oxidases; MAP, mean arterial pressure; MAPK, mitogen‐activated protein kinase; MBF, mean blood flow; MDo, myocardial oxygen delivery; min, minute; MRI, magnetic resonance imaging; MVo, myocardial oxygen consumption; NE, norepinephrine; PE, phenylephrine; PCO_2_, partial pressure of carbon dioxide; PGE2, Prostaglandin E2; PO_2_, partial pressure of oxygen; rCBF, regional cerebral blood flow; SAH, subarachnoid hemorrhage; sec, seconds; TBI, traumatic brain injury; THRR, transient hyperemic response ratio; TXB2, Thromboxane B2; x, multiplied by; 5‐HT, 5‐hydroxytryptamine.

### 3.3 NE impact on objectively measured CBF

The following subsections provide a narrative summary of the impact of NE administration on objectively measured cerebrovascular response/CBF, looking first at overall increase/decrease in CBF, followed by measured models pathology‐specific responses to NE. Table S2 of the Supplementary Materials provides a detailed tabulation of medication dosing, measurement technique, and results.

### 3.4 Increase in CBF

Twenty‐six studies demonstrated an increase in global or rCBF with the administration of NE.^17^, ^18^, ^23^, ^25^, ^36^, ^38^, ^40^, ^41^, ^45^, ^46^, ^47^, ^48^, ^49^, ^50^, ^62^, ^63^, ^64^, ^65^, ^67^, ^68^, ^69^, ^70^, ^71^, ^72^, ^75^, ^76^ The CBF increase ranged from not significant, to changes on the order of 500% of the initial CBF value.^45^ In studies which measured rCBF, all areas increased in blood flow except the auditory cortex^65^ and mesencephalon.^62^ Six studies had a dose‐dependent increase in CBF,^40^, ^45^, ^46^, ^70^, ^71^, ^72^ with one study showing a peak CBF at a NE dose of 0.16 mg/kg.^45^ The variation within the data between the individual animal models and pathologies limits the ability to draw any clear conclusions within species or technique.

### 3.5 Decrease in CBF

Twenty‐two studies demonstrated a decrease in CBF or rCBF by the administration of NE.^19^, ^21^, ^22^, ^24^, ^28^, ^29^, ^30^, ^32^, ^33^, ^34^, ^39^, ^42^, ^43^, ^44^, ^51^, ^55^, ^57^, ^58^, ^60^, ^61^, ^73^, ^74^ The CBF decrease had a wide range in variation from not significant, to a max reduction of 70%.^58^ In the single study that both monitored rCBF and CBF, an overall decrease in rCBF was seen in all areas except the brain stem.^43^

### 3.6 Direct vascular response

Of the seven studies that evaluated direct cerebrovascular response to NE,^20^, ^26^, ^27^, ^52^, ^53^, ^59^, ^77^ all had some form of constriction to the cerebral vessels. This cerebral vessel change ranged from nonsignificant up to 20% constriction as compared to baseline values.^52^, ^53^, ^59^ However, models that had a significant constrictive response to NE were either injected with another solution (a hypertonic saline solution or Wahl solution^20^) or had NE locally applied to cerebral vessels.^52^, ^53^, ^59^

### 3.7 Model‐specific responses

#### 3.7.1 Healthy models

There were 29 studies that used healthy fully anesthetized models, without a craniotomy, and assessed CBF.^17^, ^18^, ^22^, ^24^, ^25^, ^26^, ^29^, ^30^, ^31^, ^32^, ^33^, ^49^, ^57^, ^58^, ^59^, ^60^, ^61^, ^62^, ^63^, ^64^, ^65^, ^66^, ^67^, ^68^, ^69^, ^71^, ^72^, ^75^, ^76^ Five showed a nonsignificant response in CBF to NE administration.^25^, ^31^, ^57^, ^60^, ^66^ In the remaining studies there were conflicting responses seen, with NE leading to both increasing^17^, ^18^, ^22^, ^25^, ^33^, ^62^, ^64^, ^65^, ^66^, ^67^, ^68^, ^69^, ^71^, ^72^, ^75^, ^76^ and decreasing CBF.^22^, ^24^, ^29^, ^30^, ^31^, ^58^, ^60^, ^61^, ^65^ Despite these conflicting responses, there were some study design specifics to take note of.

First, the influence of coadministered substances on the effects of NE demonstrated some findings of interest. Such substances include: hypertonic saline,^18^, ^62^ phentolamine,^24^, ^33^, ^59^, ^61^ phenoxybenzamine,^32^, ^64^ and propranpol.^31^, ^32^, ^49^, ^62^ Hypertonic saline injected with NE significantly increased CBF and cerebral metabolic rate of oxygen consumption (CMRO_2_) as compared to NE alone.^18^, ^62^ Similarly, when NE was allowed to pass the blood‐brain barrier (BBB) after osmotic opening with urea, an increased regional flow was obtained.^62^ Phentolamine inhibited or completely mitigated the CBF effects of NE.^24^, ^33^, ^59^, ^61^ Likewise, phenoxybenzamine^32^, ^64^ injected with NE demonstrated that the CBF and CMRO_2_ effects of NE were decreased.^32^, ^64^ Propranolol demonstrated a decrease to CBF, when this was followed by an injection of NE CBF then increased.^31^, ^32^, ^49^, ^62^

One study testing endothelin‐1 compared hypertension with/without endothelin‐1. NE was used to induce this hypertension which caused a slight increase in CBF, with a significant CPP increase in controls. Where NE and endothelin‐1 caused the CBF and CPP to both increase significantly.^68^ NE with or without experimental renal hypertension had a similar drop in CBF from 100 to 38 mL/100 g/min. However during renal hypotension with blood loss, there was an increase in CBF followed by a return to low levels of CBF.^58^ Furthermore, a cerebral vascular resistance (CVR) increase was seen during induced hypotension (bleeding) models with NE administration (mean arterial blood pressure (MABP) of 151 mmHg in controls vs MABP of 113 mmHg in the hypotension group), which caused a slight decrease in CBF by 10%.^30^ An increase in CVR was a universal result in all the studies that evaluated CVR in healthy models.^22^, ^24^, ^30^, ^64^

#### 3.7.2 Models with craniotomy or explanted brains

In the seven studies^20^, ^23^, ^27^, ^52^, ^53^, ^54^, ^70^ that had an in vivo craniotomy, or five studies^34^, ^36^, ^37^, ^73^, ^77^ with explanted brains (to analyze cerebral vessel response), the majority of them measured cerebral vessel diameter or contraction response directly. All models demonstrated that NE either caused a constriction of cerebral vessels,^27^, ^52^, ^53^, ^70^, ^77^ or rarely they remained unaffected.^20^ In line with this, when CVR was measured there was an increase in CVR in response to NE,^34^, ^36^, ^37^ with varied response in CBF.

Using an extradural balloon to modulate ICP, one study indicated that NE had no CBF effect if the ICP was above 70 mmHg, otherwise there was a direct short‐term increase to CBF.^23^ Another study observed the pressure‐flow relationship (measured using a photoelectric drop recorder) in the brain for 30 minutes after the application of catecholamines. Based on the pressure‐flow relationship tested in each brain, the indirect effects of catecholamines on CVR caused by autoregulatory influences were calculated. This calculation was determined mathematically and then accounted for in subsequent physiological experiments, which enable the study to focus purely on the catecholamine effects in the absence of autoregulatory influences. After the autoregulatory influences were removed, NE was seen to decrease CVR by 50% and demonstrate a slight decrease in CBF.^34^

Finally, in one study, the constriction of large arterioles was induced through NE, with pial vessels remaining unchanged.^20^ While in rats, the carotid blood flow was decreased by 0.5 mL/min in all ages of animals with the injection of NE. This study also found the carotid vascular conductance was different with 0.005 mL/min in juveniles, and 0.08 mL/min in mature and middle‐aged rats, suggesting an age‐related disparity in CBF modulation.^73^

#### 3.7.3 Models given cardiopulmonary resuscitation

There were four studies in pigs that evaluated CBF while CPR was administered.^44^, ^45^, ^46^, ^47^ During CPR, NE was given in two studies at varying doses, resulting in a dose‐dependent increase to CBF.^45^, ^46^ Furthermore, increases in CMRO_2_ and CPP were also shown with the injection of NE.^47^ One of these studies had NE co‐injected with epinephrine and vasopressin, resulting in a more apparent increase in CBF, than compared to epinephrine or the vasopressin alone.^44^ In all of these studies, CBF increased with NE in comparison to control animals where NE was not given, with the NE effect on CBF observed to dissipate after 5 minutes.^44^, ^47^

#### 3.7.4 Models with traumatic brain injuries

In the four studies that had head trauma models, three of them used pigs^48^, ^50^, ^51^ and one used rats.^74^ In general, TBI caused a decrease in CBF, after the injury NE was given which caused an increase in CBF back to near baseline levels.^50^, ^51^, ^74^ The partial pressure of oxygen was also increased in the one study that monitored blood gases.^50^ One study compared the CBF effects of NE in brain‐injured piglets (fluid percussion injury) vs uninjured pigs. This study showed minor changes in CBF by NE in the uninjured pigs, but a significant increase in CBF by NE in the injuried.^51^ In the study with rat TBI models, NE administration led to an increase in ICP for 30 minutes, with a gradual decrease in CPP and slight decrease in CBF.^74^

#### 3.7.5 Other studied pathologies

There were some “other” pathologic states studied, including those with sympathectomy,^55^ induced intercranial hypertension, ^23^, ^25^, ^58^ induced hypothermia,^21^, ^35^, ^66^ brain tumors, stereotaxic induced lesions,^35^ and endotoxic shock.^43^ In the studies that had models with removed ganglion^41^, ^56^ or sympathetomy,^55^ there was a nonsignificant change tin CBF. However in models with ligated bile ducts NE both decreased CBF and increased CVR as compared to NE alone.^19^ Whereas, in dogs with a brain tumor (induced by avian sarcoma virus) NE decreased CBF in both hemispheres (one with tumor/one without) and a subsequent decrease in partial pressure of oxygen.^28^ In models with stereotaxic lesions (made in the posterior hypothalamus, unilaterally or bilateral)^35^ or endotoxishock^43^ there was limited change in CBF or practical pressure of oxygen.^35^ Finally, when NE was given in induced intracranial hypertensive states, there were massive increases in CBF with each dose of NE.^23^, ^25^, ^58^

#### 3.7.6 Anesthesia in models

In the identified literature there were six studies where the animal model was not fully anesthetized.^38^, ^39^, ^40^, ^41^, ^42^, ^55^, ^60^ Within these studies there was a dose‐dependent change CBF seen in these models^40^, ^41^ and a constrictive force seen by NE injection.^40^ However no uniform results based on anesthetic regimen were documented. In the healthy anesthesia group, pentobarbital was used in 17 studies,^17^, ^20^, ^22^, ^23^, ^24^, ^26^, ^28^, ^29^, ^31^, ^32^, ^43^, ^44^, ^56^, ^64^, ^70^, ^74^, ^77^ ketamine used in 10 studies,^17^, ^19^, ^21^, ^38^, ^40^, ^43^, ^51^, ^52^, ^53^, ^69^ as well as a variety of other substances. All displayed diverse effects of NE on CBF and CMRO_2_, with no clear trend toward a specific effect. Though for the 13 studies that used halothane,^21^, ^38^, ^45^, ^46^, ^49^, ^61^, ^62^, ^63^, ^65^, ^66^, ^68^, ^72^, ^75^ either a nonsignificant change or an increase in CBF was seen. To note, in the studies that had a CBF increase due to NE, NE was either given in large amounts (over 0.12 mg/kg),^45^, ^46^, ^63^, ^72^ with hypertonic urea^62^ or with endothelin‐1.^68^

### 3.8 Human patients

Of the remaining studies, CBF was measured with nitrous oxide,^95^ Kety‐Schmidt technique,^96^, ^97^, ^98^ gas inhaliation,^99^, ^100^ positron emission tomography,^101^ or the CMRO_2_/AVDO_2_ method (as previously stated).^102^ All failed to document a significant CBF response to NE administration. However, in those studies assessing CVR, as measure through the comparison of CBF to MAP/CPP, there was a universal increase in CVR seen.^80^, ^84^, ^87^, ^89^, ^93^, ^95^, ^97^, ^98^

Despite the multiple human studies with both healthy patients^78^, ^79^, ^80^, ^81^, ^82^, ^83^, ^98^ and patients with TBI,^86^, ^87^, ^88^, ^100^, ^101^, ^102^ CBF in most patients remained relativity unchanged. Thus, no pathology‐specific trends could be found in the human studies. There were three studies that had a nonsignificant decrease in CBF,^86^, ^98^, ^100^ and one with a nonsignificant increase in CBF,^102^ indicating a wide range of CBF response to NE. In the one study that evaluated CBF in patients with cardiac arrest through the MCAv, the flow velocity increased from 27 to 33 cm/s.^94^ Of the remaining studies no clear trends were demonstrated in the associate of NE to CBF.

### 3.9 Adverse events

No human studies document the adverse effect to NE but three animal studies included adverse events.^43^, ^64^, ^71^ Two studies reported lethal doses of NE administration.^64^, ^71^ In one study, the cause of death was determined to be the inhibition of autoregulation by NE.^64^ This study also reported that continuous moderate doses of NE for longer than 2 hours prevented autoregulation measured through autoradiography.^64^ In TBI models, there appeared to be a trend toward vasoconstriction and varying global and rCBF reductions with NE administration.

## 4 DISCUSSION

NE is commonly used to treat life‐threatening low blood pressure situations for its direct vascular effects.^2^ The scattered literature on the cerebrovascular effects of NE has produced studies displaying both a reduction and an increase in CBF, leaving a confusing picture on the exact cerebrovascular effects of the drug. The goal of this study was to provide a comprehensive systematically conducted scoping review of animal studies on NE’s effect on the cerebrovascular response/CBF. Through our review we identified 62 animal studies^16^, ^17^, ^18^, ^19^, ^20^, ^21^, ^22^, ^23^, ^24^, ^25^, ^26^, ^27^, ^28^, ^29^, ^30^, ^31^, ^32^, ^33^, ^34^, ^35^, ^36^, ^37^, ^38^, ^39^, ^40^, ^41^, ^42^, ^43^, ^44^, ^45^, ^46^, ^47^, ^48^, ^49^, ^50^, ^51^, ^52^, ^53^, ^54^, ^55^, ^56^, ^57^, ^58^, ^59^, ^60^, ^61^, ^62^, ^63^, ^64^, ^65^, ^66^, ^67^, ^68^, ^69^, ^70^, ^71^, ^72^, ^73^, ^74^, ^75^, ^76^, ^77^ and 26 human studies^78^, ^79^, ^80^, ^81^, ^82^, ^83^, ^84^, ^85^, ^86^, ^87^, ^88^, ^89^, ^90^, ^91^, ^92^, ^93^, ^94^, ^95^, ^96^, ^97^, ^98^, ^99^, ^100^, ^101^, ^102^, ^103^ pertaining to the cerebrovascular/CBF effects of NE. Within the 62 animal studies, a variety of different models were used, with the majority focusing on changes in global CBF or rCBF. A minority of studies focused on the direct effects of NE on the cerebral vasculature.^26^, ^27^, ^37^, ^52^, ^53^, ^59^, ^77^ Overall, regardless of the model or modality of measurement, NE led to a vasoconstrictive effect in medium cerebral vessels in a dose‐dependent manner, with no clear directional change to either global CBF or rCBF. Pial vessels seemed to remain unaffected. However, significant heterogeneity in study design, models, and outcome assessment limits the degree to which these results can be interpreted and translated to clinical practice. Some important points can be gleaned from this review.

First, NE administration in animals leads to a vasoconstriction of medium cerebral vessels.^26^, ^27^, ^37^, ^52^, ^53^, ^59^, ^77^ This is in the setting of constant pCO_2_ and pO_2_ during the experiments. The literature demonstrating an effect on pial vasculature was limited, with only one study which demonstrated no change to their diameter.^53^ Furthermore this constrictive effect was shown to be inhibited by alpha adrenergic blockers like phenoxybenzamine and phentolamine in animal models,^26^, ^32^, ^33^, ^37^, ^39^, ^41^, ^59^, ^61^, ^64^ and in one human study.^80^ Given the relative homogeneity of the studies on NE vasoconstrictive traits and the inhibition by alpha adrenergic drugs, it can be inferred that NE stimulates alpha receptors to contract vessel within the brain, similar to NE’s effect on other systemic vessels. This general feature, found across different species of animal models, different model types from healthy to injured, and different sedation regimens, carries important implications for the application of the agent in humans with critical neurological illness. Direct cerebral vasoconstriction from NE may expose the brain to wider derangements in cerebral autoregulation/cerebrovascular reactivity, and lead to episodes of hyperemia or ischemia. Further to this, if NE administration were to abolish or eliminate cerebral autoregulatory capacity altogether, as seen on some of the animal studies identified, this could lead to catastrophic consequences.^43^, ^64^, ^71^ These consequences are particularly important in TBI patients, where it is well known that impaired cerebrovascular reactivity is strongly associated with outcome,^6^, ^104^, ^105^, ^106^ and is present in many patients during their ICU stay and remains refractory to treatment effects.^105^, ^107^ It also carries implications for the use of vasopressor agents in the targeting of individualized physiologic targets in TBI based on continuous cerebrovascular reactivity monitoring.^8^, ^9^, ^10^, ^11^, ^12^, ^13^ Though, it must be acknowledged, these results from animal models and one human study may not translate directly to all humans and requires future investigation in both large animals and humans with TBI.

Second, the data are not clear regarding the change in global and rCBF with the injection of NE and why there appears to be such a discrepancy of response between studies and models. In healthy and CPR animal models, there was a trend toward a dose‐dependent increase in CBF. However, in TBI and other cerebral lesion models, the impact of NE on CBF was heterogeneous, in the setting of constant/controlled pCO_2_ and pO_2_. Sedation regimen did not seem to impact these findings based on the available data in the parent manuscripts. In such acquired brain injury models, it is possible that the CBF reductions seen can be more directly associated with the alterations in CBV, and thus ICP, occurring with NE‐based cerebral vasoconstriction, as opposed to any direct flow augmenting effect of NE. However, in some studies that measured CBV and CBF, the data demonstrated a positive linear connection between them during NE administration.^22^, ^25^, ^55^, ^72^ Furthermore, such acquired brain injury states may lead to regional disparities in blood‐brain barrier (BBB) functionality. Areas of impaired BBB integrity may lead to more extracellular deposition of NE, leading to direct action on both the vasculature and cellular support network, causing variability in CBF response seen. Such BBB impacts on NE effects may be important, as healthy data suggest that an intact healthy barrier prevents much of the systemic catecholamines from entering the extracellular space. Further investigation is required into the regional disparities of CBF secondary to NE in the context of acquired brain injury.

Furthermore, the injection of NE through systemic routes may have effects different than NE directly injected within the brain. NE injected with hypertonic urea or MgSO_4_ solution resulted in an increase in CBF with the same dose of NE. As such it is likely that the BBB mediates the perfusion of NE throughout the brain and its effects on CBF.^18^, ^40^, ^62^ This point may also by enforced by the fact that during studies where animals had lesions that opened the BBB, an increase in CBF after NE injection was seen.^40^ Also in studies with impaired autoregulation there was a consistent response to NE with an increase in ICP and CBF.^23^, ^24^ All these findings support a potential role for the BBB in the regulation on cerebrovascular response to NE. As mentioned above, in line with this, NE given systemically may not enter the brain parenchyma due to the BBB, though it is clear that the BBB limits the permeation of NE it may not prevent all of the NE from entering the BBB.^18^, ^108^ This particular area of BBB integrity, its impact on NE‐based cerebrovascular/CBF responses in acquired brain injury, is an area requiring much further investigation.

Third, six studies demonstrated that the exogenous administration of NE reaches a maximal effect on cerebrovascular response.^20^, ^29^, ^31^, ^32^, ^45^, ^71^ All of these studies compared various doses of NE which resulted in a maximum change in both CBF and CVR of the animal models. Thus, a dose‐dependent response to NE occurs, which again carries important implications for continuous cerebrovascular reactivity monitoring and derivation of individualized physiologic target in TBI. However, a universal max dose of NE, in terms of CVR effect, could not be demonstrated due to the heterogeneity within the studies, and is unlikely to exist in vivo in humans. NE‐dosing thresholds and their impact on continuously monitored cerebrovascular reactivity/CBF in vivo in critically ill neurological patients, such as the TBI population, have not been conducted, and require further investigation.

Finally, unwanted cerebral physiologic side effects of NE administration were seen. Demonstration of NE’s impact on ICP was shown by using an extradural balloon to increase ICP. NE had no effect on the overall ICP, unless ICP was at the extreme pressure of over 70 mmHg.^23^ Furthermore in studies that measure CBV and CBF, the data demonstrated a positive linear connection between them.^21^, ^25^, ^55^, ^72^ This linear connection encourages the idea that potentially the change in CBF has more to do with alterations in CBV than the CVR effect of NE. The inhibition of autoregulatory hemodynamics within the brain by NE injections was also described.^64^ Prolonged or long continuous injections have resulted in lethal inhibition to cerebral hemodynamics, as highlighted in two studies.^64^, ^71^ As mentioned above, regarding individualized physiologic targets in TBI care, this aspect of prolonged high‐dose NE administration needs to be considered and investigated further.

### 4.1 Limitations

In this review we have been able to systematically, and comprehensively, document the current literature on the cerebrovascular/CBF effects of NE. There is a trend in the animal literature of a vasoconstriction of cerebral vessels seen with NE administration, with conflicting results regarding global and rCBF responses, depending on the presence of acquired brain injury. However, caution must be taken as our review has several limitations. The studies are quite heterogenous in design and species, with mixed results. The animal studies, given heterogeneity and potential species‐specific responses, limits our ability to translate these results to the clinical application of NE in humans regardless of the underlying pathology. Furthermore most human studies measured CBF through an assumption of MCAv, this is not a true measure of global CBF or rCBF. Another limitation is the lack of blood gas control in some of the studies. Cerebrovascular/CBF physiologic response is intimately linked to pCO_2_ and pO_2_ status, therefore due to the large number of studies that did not fully account for fluctuations in the blood gas level, leaves any conclusion linked with NE deficient. Last, although there are trends in the animal models, there is still a significant limitation to apply them in clinical practices simply based on the limited number of effect human studies.

### 4.2 Future directions

Further prospective studies on the cerebrovascular/CBF effects of NE in the neurologically ill patient population need to be performed to determine the role of this medication within neuroanesthesia and the neuro‐ICU. The potential CBF trends seen with NE are interesting and carry important implications in the treatment of a variety of cerebral pathologies, with TBI mentioned as exemplar given that CBF and cerebral autoregulation are key factors to improve patient outcome. When it comes to TBI, literature in the field of moderate/severe TBI has demonstrated that impaired cerebral autoregulation/cerebrovascular reactivity is directly associated with poor 6‐month global outcome.^6^, ^104^, ^106^, ^109^, ^110^ This has been validated in prospective multicenter data,^106^ and recent retrospective data sets suggest that cerebrovascular reactivity remains unaffected by changes in guideline‐based management of TBI over the last 25 years, in concert with relatively stable mortality rates.^105^ Such findings suggest that despite improvement in ICP and CPP targeting, cerebrovascular reactivity remains resistant to current therapeutic measures in moderate/severe TBI care, and may be a main contributor to persistently high mortality rates despite advancements in therapeutic targeting. There currently exists limited literature on the impact of commonly administered therapies in TBI, such as NE, and their impact on cerebrovascular reactivity, with most suggesting an unclear association.^111^ Cerebrovascular reactivity monitoring is being adopted to direct personalized physiologic targets in TBI care, including optimal CPP targeting,^10^, ^13^, ^112^ with the expectation that such personalized approaches based on cerebrovascular monitoring will be extrapolated to other neuropathological states.^7^, ^113^, ^114^, ^115^, ^116^, ^117^ Such concepts are currently being explored in phase II clinical trials.^13^ Thus, knowledge of the impact of commonly administered vasoactive compounds, such as NE, on the cerebrovascular response is crucial if we are to truly move toward such personalized medicine approaches. Future studies require controlled evaluation of the NE effect on cerebrovascular reactivity/CBF, in both large animal and humans. Such work would benefit from the continuous evaluation of cerebrovascular reactivity, through such methods as the pressure reactivity index (PRx), with other concurrent multimodal cerebral physiologic monitoring, such as brain tissue oxygen, parenchymal CBF monitoring, and cerebral microdialysis. Such work would provide important insights into the true cerebrovascular and cerebral physiologic impacts of NE.

In addition to this evaluation of NE in TBI with advanced multimodal monitoring of cerebrovascular response, further animal models are required. As seen in the described literature body, the presence of TBI or other acquired brain injury may lead to different CBF responses compared to healthy animals/humans. This suggests a potential role for the regional disparities in BBB integrity mitigating the cerebrovascular/CBF response to NE. Future work into NE models with BBB disruption is required to provide insight into the impact of BBB integrity on NE effects. In parallel to this, the control mechanisms involved in cerebral autoregulation are multifaceted, and are likely the impact of individual genetic polymorphisms in humans.^118^, ^119^ Future work, both in humans and genetically controlled animal models may also shed insight into variances in cerebrovascular/CBF catecholamine responses. All such work mentioned requires substantial coordination between multiple centers of excellence/expertise, and requires multidisciplinary research teams. This is the focus of ongoing collaborative work in Europe^120^, ^121^ and Canada.^122^

## 5 CONCLUSIONS

The animal models indicate an increase in vasoconstriction with NE administration through the alpha receptor in vessels. There appeared to be a dose‐dependent increase in CBF with NE administration in healthy and CPR animal models, which was also seen in one human study. However, there was no clear trend to describe the global and rCBF changes seen during the injection of NE in models with TBI, acquired brain injury, or within any other group of human patients. Further investigation into the impact of NE on cerebrovasculature in large animal models and humans is required.

## DISCLOSURE

There is no conflict of interest by any of the authors in the work presented.

## ETHICAL STATEMENT

There were no trial or experiments preformed in this systematic review as such all ethics outlined by the WMA Declaration of Helsinki or the Ethics regarding animal testing are not applicable. Further all article references are fully published and have been vetted by their respective journals.

## Supporting information

Appendix A‐B

Appendix C

Table S1‐S2

## Data Availability

Data derived from public domain resources. The data that support the findings of this study are available in MEDLINE, BIOSIS, EMBASE, Global Health, SCOPUS, or Cochrane Library.
