# Supplementary material for: The cerebrovascular response to norepinephrine: A scoping systematic review of the animal and human literature
Source: Pharmacol Res Perspect. 2020 Sep 23;8(5):e00655. doi: 10.1002/prp2.655 (PMC7510331; doi:10.1002/prp2.655)
Supplement: Supplementary file 2 — Appendix C [file PRP2-8-e00655-s002.docx]

 Supplementary Appendix C

Table A: Included Studies – General Characteristics and Study Goals

| **References** | **No. Patients** | **Study Type** | **Mean Age** | **Patient Characteristics** | **Primary and Secondary Goal of Study** |
| --- | --- | --- | --- | --- | --- |
| **Healthy Patients** | | | | | |
| Brassard et al^78^ | 6 patients | Prospective cohort study | 26±6 years | Healthy male patients | Primary: Effect of NE on cerebral oxygenation |
| Greenfield et al^79^ | 22 patients | Prospective cohort study | Greater than 17 years | Healthy male patients | Primary: Effect of NE, E, and angiotensin on ICAv |
| Kimmerly et al^80^ | 7 patients | Prospective cohort study | 21-42 years | Healthy patients | Primary: NE influence on cerebrovascular control in conscious humans |
| Moppett et al^81^ | 7 patients | Prospective cross-sectional study | 18-40 years | Healthy patients | Primary: The effects of NE and glyceryl trinitrate on cerebral hemodynamics in healthy volunteers |
| Sensenbach et al^98^ | 40 patients NE=12  E =15 USP E =13 | Prospective cohort study | 24-40 years | Healthy patients | Primary: Compare the effects of NE, E and USP E on CBF and metabolism in man |
| Sorenson et al^82^ | 15 patients | Prospective cohort study | 25±5 years | Healthy male patients | Primate: NE influence on cutaneous vasoconstriction |
| Strebel et al^83^ | 40 patients | Prospective Random Cohort Study | 39±8 years | Patients anesthetized with isoflurane or propofol | Primary: Impact of system vasoconstrictors on cerebral circulation of anesthetized patients |
| **Patients with Hypotension** | | | | | |
| Fazekas et al^97^ | 10 patients | Prospective cohort study | 42-87 years | Patients with hypotension | Primary: Effect of NE on cerebral hemodynamics in severe hypotension |
| Gottstein et al^103^ | Not mentioned | Review Article | Not mentioned | Patients with hypotension | Primary: Pharmacological influence of drugs on CBF |
| **Patients with Stroke** | | | | | |
| Georgiadis et al^84^ | 14 patients | Prospective cohort study | 58±11 years | Patients with acute stroke (HSS>15) and some with hypothermia 33ºC | Primary: CA in moderate hypothermia in patients with acute stroke  Secondary: Influence of NE on MCAv |
| Schwarz et al ^85^ | 19 patients Female =8  Male =11 | Prospective cohort study | 39.1± 2.4 years | Patients with acute, complete, or subtotal MCA territory stroke | Primary: Effects of induced arterial hypertension in patients with large ischemic stroke  Secondary: Effects of induced hypertension on ICP and MCAv in patients with large hemispheric stroke |
| \| **Patients with TBI** \| \| --- \| | | | | | |
| Coles et al^101^ | 30 patients | Prospective cohort study | 16-68 years | 10 healthy patients and 20 with TBI, GCS 3-9 | Primary: Hypertension’s influence on cerebral ischemia within TBI |
| Chieregato et al^100^ | 16 patients Female =12 Male =4 | Prospective cohort study | 44.4±19.7 years | Head injured patients with contusions larger than 2 cm, GCS 3-8 | Primary: To evaluate the response to an acute elevation of CPP of the rCBF measured in the edematous area of traumatic contusions  Secondary: Compare healthy to low density brain tissue |
| Johnston et al^102^ | 11 patients Females =3  Males =8 | Prospective controlled trial | 28±16 years | Head injury requiring vasopressor to support CPP, GCS 3-9 | Primary: Effect of CPP augmentation with dopamine and NE on global and focal brain oxygenation after TBI |
| Mascia et al^86^ | 12 patients | Prospective interventional study | 15-63 years | Severely head injured patients, GCS 3-8 | Primary: NE management of MAP and cerebral hyperemia after severe head injury  Secondary: Difference between Xenon^133^ inhalation technique, jugular bulb oxygen saturation and transcranial Doppler |
| Steiner et al^87^ | 10 patients Females =3  Males =7 | Prospective randomized crossover trial | 37±16 years | Acutely head injured patients requiring vasoactive drugs, GCS 3-9 | Primary: Compare the cerebrovascular effects of NE and dopamine in patients with acute TBI |
| Ract et al^88^ | 19 patients | Prospective cohort study | 18-53 years | Patients with severe TBI, GCS<8 | Primary: Cerebral effects of dopamine and NE |
| **Miscellaneous** | | | | | |
| Larsen et al^89^ | 7 patients Female=6 Males =1 | Prospective cohort study | 18-55 years | Patients with fulminant hepatic failure | Primary: rCBF autoregulation in patients with fulminant hepatic failure |
| Toksvang et al^90^ | 8 patients Female =2 Male =6 | Prospective cohort study | 62±11 years | Severe sepsis or septic shock diagnosed within the past 72 hours | Primary: Compare transcranial Doppler and NIRS based estimates of CBF  Secondary: Change in CBF due to NE |
| Strauss et al^91^ | 7 patients | Prospective cohort study | 30-50 years | Patients with acute liver failure | Primary: Hyperventilation influence on autoregulation in patients with acute liver failure  Secondary: Hyperventilation vs NE on CBF |
| Van Den Brule et al^92^ | 50 patients | Prospective cohort study | 18-35 years | Healthy male subjects during experimental human endotoxemia and severe sepsis or septic shock | Primary: Influence of vasopressor on cerebral critical closing pressure during systemic inflammation |
| Caronna et al^99^ | 4 patients | Prospective cohort study | 41-69 years | Patients with chronic idiopathic autonomic insufficiency | Primary: Cerebrovascular regulation in preganglionic and postganglionic autonomic insufficiency |
| Moller et al^96^ | 7 patients | Prospective cohort study | 26-27 years | Patient with severe bacterial meningitis | Primary: CBF and metabolism during infusion of NE and propofol in patients with bacterial meningitis |
| Berg et al^93^ | 8 patients | Prospective cohort study | 23±2 years | Healthy patients with induced inflammation | Primary: NE influence on dynamic CA in systemic inflammation |
| King et al^95^ | 9 patients | Prospective cohort study | 19-50 years | Healthy patients with induced inflammation | Primary: Effects of E and NE upon cerebral circulation and metabolism |
| Sundgreen et al^94^ | 24 patients | Prospective cohort study | 21-81 years | 6 healthy patients an 18 with cardiac arrest | Primary: Autoregulation of CBF in resuscitation from cardiac arrest |
| \| CA, cerebral autoregulation; CBF, cerebral blood flow; CPP, cerebral perfusion pressure; E, epinephrine; GCS, Glasgow coma score; HSS, hemispheric stroke scale; ICAv, internal carotid artery velocity; ICP, intracranial pressure; MAP, mean arterial pressure; MCA, middle cerebral artery; MCAv, Middle cerebral artery velocity; NE, norepinephrine; NIRS, near infrared Spectroscopy; rCBF, regional cerebral blood flow; TBI, traumatic brain injury; USP, United states pharmacopeia; \| \| --- \| | | | | | |
|  |  |  |  |  |  |

Table B: Norepinephrine Treatment and Cerebrovascular Response – Study Details

| **References** | **Dose** | **Mean Duration of Dose Administration** | **Technique to Measure Cerebrovascular Response** | **Cerebrovascular Response** | **Other Outcome** | **Conclusions** |
| --- | --- | --- | --- | --- | --- | --- |
| **Healthy Patients** | | | | | | |
| Brassard et al^78^ | NE: 0.05, 0.1 and 0.15 ug/kg/min | 20 minutes | ScO_2_: NIRS  MCAv: Transcranial Doppler  PO_2_ and PCO_2_: Blood samples | ScO_2_ decreased from 78 to 69%  MCAv has no significant change  **PCO_2_ remained constant** |  | NE may negatively influence oxygenation with limited influence on CBF |
| Greenfield et al^79^ | NE: 0.25 to 8ug  E: 3ug/min  Angiotensin: 1 ug/min | 5 minutes  NE used to increase MAP | ICAv: Transcranial Doppler  MAP: Radial arterial pressure  PCO_2_: Blood samples | NE and E increased MAP but decreased ICAv both were not significant  Angiotensin increase CBF and MAP  **PCO_2_ was relatively constant** |  | NE and E decrease ICAv which indicates minor decrease in CBF though this may be due to the constrict that they cause |
| Kimmerly et al^80^ | NE: 50-100 ng/kg/min  Phentolamine: 200 to 100 ug | 15 minutes | MCAv: Transcranial Doppler  CVR: MAP/MCAv  MAP: Tonometry  ECO_2_: Gas measure  PCO_2_: Blood samples | NE and Phentolamine increased MCAv but only NE increased CVR at high doses by 0.3mmHg*s/cm  **PCO_2_ remained constant, PO_2_ was not measured** |  | NE had little influence on CBF and was seen to be inhibited by phentolamine |
| Moppett et al^81^ | NE: 0.02- 0.1ug/kg/min  Glyceryl trinitrate: 0.1-2.5ug/kg/min | Increase MAP by 25% for NE  Decrease MAP by 15% for Glyceryl trinitrate | MCAv: Transcranial Doppler  CPP: Transcranial and radial transducer  ECO_2_: Gas samples | **NE:** MCAv: No change CPP: No significant change   **Glyceryl trinitrate:** MCAv: Decreased slightly  CPP: No significant change  **ECO_2_ was constrained, O_2_ was not accounted for** |  | NE and Glyceryl trinitrate had little influence on cerebral hemodynamics |
| Sensenbach et al^98^ | NE, E and USP E: 600-1400 ug | 15-20 minutes | CBF: Kety-Schmidt technique   CMRO_2_: Calculated by CBF and AVDO_2_  AVDO_2_: Blood samples  CVR: Calculated from CBF and MAP  MAP: Catheter | **NE:** CBF decreased from 61 to 48 cc/min/100g CVR: Increased by 64% (p<0.01) AVDO_2_ increase from 6.59 to 7.96% CMRO_2_ was unaffected  **E** had no significant effect on CBF, CVR, CMRO_2_, arterial or venous oxygen content  **USP E** a slight but significant reduction in MAP without changes in CBF, CVR, CMRO_2_, arterial or venous oxygen content | NE results in powerful cerebral vasoconstriction in normal healthy males and increases in perfusion pressure consequently the blood flow to the brain is diminished. This does not affect cerebral oxygen consumption. | NE increases MAP and reduces CBF in normotensive males by virtue of its potent constricting effect upon cerebral blood vessels. Cerebral oxygen utilization is not altered.  E and USP E demonstrated little effect on cerebral hemodynamics |
| Sorenson et al^82^ | NE: 15ug/g/min | 15 minutes | ScO_2_: NIRS  MCAv: Transcranial Doppler  PO_2_ and PCO_2_ Blood samples | MCAv: Remains relativity constant through most groups except for hyperventilation which had a significant decrease  CBF: Remained constant ScO_2_: Remained constant   **PCO_2_ and PO_2_ maintained with ventilation** |  | NE had little influence on MCAv and CBF |
| Strebel et al^83^ | Propofol: 2 mg/kg  Isoflurane: 1 MAC  Phenylephrine and NE not specified | Not mentioned | CBF: Laser-Doppler flowmetry in MCA and carotid artery  PCO_2_ and PO_2_: Blood samples | Both increased MCAv by 5cm/s and ICAv by 4 cm/s in isoflurane but not in propofol  **PCO_2_ and PO_2_ remained constant** |  | NE and Phenylephrine do not directly affect intracranial hemodynamics in anesthetized patients |
| **Patients with Hypotension** | | | | | | |
| Fazekas et al^97^ | NE: 4 to 32 ug/cc | 30 minutes | CBF: Scheinberg-Stead's modification of Kety-Schmidt Technique  PO_2_: Van Slyke and  Neill Technique | CBF increased from 34.8 to 44.8 cc/100g/min with CVR increasing from 1.6 to 2.6  **PCO_2_ was not accounted for** |  | NE increased CBF and CVR but had little influence on CMRO_2_ |
| Gottstein et al^103^ | Not mentioned | Not mentioned | CBF: Not motioned | NE increase CBF in 4 studies  **PCO_2_ assumed to be constant** |  | NE may increase CBF though this conclusion is limited due to lack of evaluated factors like O_2_ |
| **Patients with Stroke** | | | | | | |
| Georgiadis et al^84^ | Not mentioned | NE increase MAP by 10% for 2 hours | MCAv: Transcranial doppler  MAP: Radial artery catheter  ICP: Parenchymal catheter  CVR: MAP/MCAv | MCAv in both groups ranged from -9 to 32%  CVR increase from 8 to 36% in both group  **PCO_2_ and PO_2_ assumed to be constrained through ventilation** |  | Hypothermia resulted in little influence of cerebral autoregulation  NE had various responses in MCAv therefore its effect on CBF is unknown |
| Schwarz et al ^85^ | NE: infusion rate of 2 mg/hour or baseline increased by 10% | Achieve a MAP increase of at least 10 mm Hg | ICP: Parenchymal catheter  MCAv: Transducer of a pulsed wave ultrasound machine  MAP: Radial transducer | ICP slightly increased from 11.6±0.9 mmHg to 11.8±0.9 mm Hg (p=0.05)   CPP rose from 72.2±2 mmHg to 97±1 mmHg (p=0.0001)  MCAv remained high 25.5±5.5 cm/s on the affected side and by 8.6±1.6cm/s on the contralateral side  **PCO_2_ and PO_2_ assumed to be constant through ventilation** | Safety and efficacy of prolonged phases of arterial hypertension are potential hazards if used in patients with markedly raised ICP | Indicates that NE-induced hypertension during a short period of time improves CPP, augments MCAv, and slightly raised ICP in patients with large MCA stroke in the absence of acute ICP crises |
| \| **Patients with TBI** \| \| --- \| | | | | | | |
| Coles et al^101^ | NE: 0-0.33ug/kg/min  to main CPP at 70 and 90 mmHg | 10 minutes | CBF, CBV, CMRO_2_ were calculated by inputting simultaneous PET and arterial tracer activity measurement  PO_2_ and PCO_2_ Blood samples | NE increase CPP by 12 mmHg and ICP by 2 mmHg  CBF and CBV slightly increased but was not significant   CMRO_2_ decrease slightly  **PCO_2_ remained constant after NE** |  | NE has little influence on CBF or Cerebral metabolism |
| Chieregato et al^100^ | NE induce 20 mmHg elevation of CPP levels | 20 mins | CBF: Picker 5000 CT scanner equipped for Xenon-CT CBF imaging  ICP: Intracranial transducer  PCO_2_ and PO_2_: Arterial blood samples | rCBF: Decreased from 34.9±13.6 to 27.9±13.5 ml/100g/min (p=0.0041)  CPP: 65.8± 8.6 to 88.7± 8.9 mmHg (p<0.0001)  ICP: 22.6±7.5 to 23.9± 8.8 mmHg (p=0.1126)  **PCO_2_ and PO_2_ were maintained through ventilation** | Change in rCBF was inversely associated to the baseline value. | CPP elevation induced by NE maybe effective in improving rCBF |
| Johnston et al^102^ | NE: 0.086±0.066 ug/kg/min 0.146±0.103ug/kg/min   Dopamine: 6.4± 5.5 and 13.1±9.2 ug/kg/min | Achieve and maintain a CPP of 60-70 mmHg | AVDO_2_: Blood samples  CBF: CMRO_2_/AVDO_2_ with CMRO_2_ assumed to be constant  CPP: Cerebral tissue gas with a multimodal sensor, and regional chemistry was assessed using micro dialysis catheter  ICP, PCO_2_ and PO_2_: Micro dialysis catheter | **NE overall:** AVDO_2_: reduced from 37±11 to 33±12 ml/l  Brain tissue oxygen: Significant increase 2.6±1.1 to 3.0±1.1kPa  ICP: No significant change  **Dopamine:** CPP slightly higher than NE but all other values remain constant  **PO_2_ used a oxygen reactivity test to account for changes**  **PCO_2_ remained constant** | There were large inter-individual differences in the doses of catecholamines required to achieve the target CPP levels. This variability suggests considerable pharmacodynamic variability and that dosing regimes based on weight are not useful in critically ill patients | There are no significant differences between NE and dopamine on cerebral oxygenation or metabolism   NE may increase CBF but this conclusion is limited by CMRO_2_ assumption |
| Mascia et al^86^ | NE: 0.006-0.1 ug/kg/min | Maintain CPP at 70 mmHg | CBF: Xenon^133^ inhalation technique  MCAv: Transcranial Doppler  CVR: CBF/CPP  PCO_2_ and PO_2_: Blood samples  Impaired autoregulation: CPP/CVR < 2 | **NE:**  CPP increased by 33%  CBF decreased from 31±3 to 28±3 ml/100 g/min ScO_2_increased from 67% to 73%  MCAv increased from 59 to 63 cm/s   Two patients with impaired autoregulation found CBF increased respectively from 16 to 35 and from 21 to 70 ml/100 g/min  **PCO_2_ and PO_2_ maintained through ventilation** | The assessment of pressure autoregulation should be considered as a guide for arterial pressure-oriented therapy after hypertension | During CPP management NE can be used to increase MAP without potentiating hyperemia if pressure autoregulation is preserved  In patients with intact autoregulation NE decrease CBF and increases MCAv |
| Steiner et al^87^ | NE and Dopamine: Increase CPP to 65, 75 and 85 mmHg | 20 minutes | MAP and ICP: Intraparenchymal probe  MCAv: Transcranial Doppler  CVR: CPP/CBF  PCO_2_: Marquette Solar 8000M | **NE:** CVR: Was significantly higher at each CPP increase (p<0.05) ICP: Remained relatively stable for all CPP levels  MCAv: Increased by 20% with CPP increase  **Dopamine:** CVR: Was significantly higher at each CPP increase (p<0.05) ICP: Remained relatively stable for all CPP levels  MCAv: Had various response  **PCO_2_ maintained with ventilators PO_2_ not accounted for** |  | NE may be more predictable and efficient to augment CPP in patients with TBI then dopamine, in this way it may be effective at increasing MCAv and CBF |
| Ract et al^88^ | NE and dopamine were used to maintain MAP | Not mentioned | MCAv: Transcranial Doppler  ICP: Intracranial transduced  PO_2_ and PCO_2_: Blood samples | Dopamine was seen to increase ICP and MCAv as compared to NE  **PCO_2_ maintained through ventilation** |  | Despite the great influence of dopamine in MCAv there is little influence on CBF and NE had no effect |
| **Miscellaneous** | | | | | | |
| Larsen et al^89^ | NE: 5-15 ug/min | MAP continuously increased by at least 30 mmHg | CPP: Calculated by MCAv - Resistance  MCAv and anterior cervical artery velocity: Transcranial Doppler  Resistance: Pulpability index   PCO_2_: Blood samples | MCAv: Increased in the by anterior cerebral artery 28% and MCA by 19% (p<0.05)  Resistance: Anterior 1.02 and MCA 0.87 (p <0.01)  PCO_2_ and PO_2_ maintained through mechanical ventilation |  | MCAv and anterior cerebral artery velocity increase with indicates an increase in CBF by NE through this is not confirmed |
| Toksvang et al^90^ | NE: 0.04–0.18 ug/kg/min. | MAP was increased in 5 mmHg increments over 3–7 steps for 4-5 min/step | CBF ScO_2_: NIRS  MCAv: transcranial Doppler ultrasound  PCO_2_ and PO_2_: Blood samples | CBF ScO_2_: No changes  MCAv: Increase of 14% (p<0.05)   **PCO_2_ and PO_2_ remained constant** |  | Transcranial doppler and NIRS cannot be used interchangeably for monitoring changes in cerebral hemodynamics in critically ill patients and furthermore NE had a varying response on CBF |
| Strauss et al^91^ | NE: 0.5-10 ug/h to increase MAP by 20-30mmHg | 10-20 minutes | MCAv Transcranial doppler  MAP: Arterial catheter  PCO_2_: Blood samples | **NE:** increase MCAv from 47 to 68 cm/s and MAP from 82 to 106 mmHg  Hyperventilation’s does not increase MCAv   **PCO_2_ remained relatively constant, PO_2_ not accounted for** |  | Net increase MCAv but this may not translate to CBF |
| Van Den Brule et al^92^ | NE: 0.05ug/kg  PE:0.5ug/kg/min  Vasopressin: 0.04IU/min  Lipopolysaccharide: 2ng/kg | 5 hours | MCAv: Laser-Doppler flowmetry | Lipopolysaccharide caused a slight decreased MCAv  All groups had no effect on MCAv  **PCO_2_ and PO_2_ assumed to be constant** |  | Vasopressors had little influence on CBF |
| Caronna et al^99^ | NE: 1 to 9 ug/min | 2 hours | CBF: Krypton^80^ inhalation  PCO_2_, PO_2_ and CMRO_2_: Blood samples | CBF or CMRO_2_ had little response to NE  **PCO_2_ and PO_2_ remained constant** |  | NE had little influence on CBF |
| Moller et al^96^ | NE: 0-0.5 ug/kg/min used to increase MPA by 20 mmHg  Propofol 0-600 mg/hr | 10 minutes | CBF was measured by the Kety–Schmidt technique  CMRO_2_: Calculated from CBF and AVDO_2_  AVDO_2:_ Blood samples | **NE:** CMRO_2_ decreased and increased CBF in patients  **Propofol:** CMRO_2_ and AVDO_2_ decreased whereas CBF was unchanged  **PCO_2_ assumed to be constant through ventilation** |  | NE and propofol demonstrated little effect on CBF or CMRO_2_ |
| Berg et al^93^ | NE: 0.13-0.27 ug/kg/min to increase MPA 25-30 mmHg | 20 minutes | MCAv: Transcranial Doppler  PCO_2_: Blood samples  CVR: MAP/MCAv | MCAv: decreased from 1.18 to 0.93 cm/mmHg/s  PCO_2_ slightly decrease due to NE injection  CVR increased by 0.3 |  | NE slightly decreased MCAv but little overall CBF change |
| King et al^95^ | NE: 200-770 ug  E: 480 - 1732 ug | Not mentioned | CBF: Nitrous oxide method  MAP: Manometric   CMRO_2_ and CVR: Calculated from MAP and CBF  PCO_2_ and PO_2_ blood samples | **NE** increase CVR from 1.56 to 2.23 mmHg/cc/100g/min  CBF: Non-significant decrease  AVDO_2_: Non-significant increase  **E** increased CBF by 10cc/100g/min with little influence on other measured cerebral responses  **PCO_2_ and PO_2_ found to be constant** |  | NE had an increase to CVR with no influence on CBF  E increased CBF |
| Sundgreen et al^94^ | NE used to increase a stepwise of MAP amounts not mentioned | Up to 2 hours | MCAv: Transcranial doppler  MAP: Radial artery catheter  ICP: Parenchymal catheter  CVR: MAP/MCAv | MCAv: increased in patients from 27 to 33 cm/s with a MAP from 78 to 106 mmHg  **PCO_2_ and PO_2_ were maintained through ventilation** |  | NE was seen to increase MCAv as a result form MAP increase |
| AVDO_2_, arterial venous oxygen difference; CA, cerebral autoregulation; CBF, cerebral blood flow; cc, cubic centimeter; CMRO_2_, cerebral oxygen consumption; CO_2_, carbon dioxide CPP, cerebral perfusion pressure; CT, computed tomography; CVR, cerebrovascular resistance; E, epinephrine; ECO_2_, end tidal CO_2_;GCS, Glasgow coma score; HSS, hemispheric stroke scale; ICAv, internal carotid artery velocity; ICP, intracranial pressure; MAP, mean arterial pressure; MCA, middle cerebral artery; MCAv, Middle cerebral artery velocity; mmHg, millimeters of mercury; NE, norepinephrine; NIRS, near infrared Spectroscopy; PCO_2_, partial pressure of carbon dioxide PO_2_, partial pressure of oxygen; rCBF, regional cerebral blood flow; ScO_2_, cerebral oxygen saturation; TBI, traumatic brain injury; USP, United states pharmacopeia; | | | | | | |
