# Supplementary material for: The cerebrovascular response to norepinephrine: A scoping systematic review of the animal and human literature
Source: Pharmacol Res Perspect. 2020 Sep 23;8(5):e00655. doi: 10.1002/prp2.655 (PMC7510331; doi:10.1002/prp2.655)
Supplement: Supplementary file 3 — Table S1‐S2 [file PRP2-8-e00655-s003.docx]

Table 1: Included Studies – General Characteristics and Study Goals

| **Reference** | **Number of Animals** | **Study Type** | **Model Characteristics** | **Primary and Secondary Goal of Study** |
| --- | --- | --- | --- | --- |
| **Healthy Heavily Anesthetized Animal Models** | | | | |
| McCalden et al, 1979^17^ | 15 baboons | Three arm study | Healthy baboons anesthetized with ketamine hydrochloride and sodium pentobarbital | Primary: Role of catecholamine degradative enzymes and the adrenergic innervation in determining the cerebrovascular response to infused NE |
| MacKenzie et al, 1976^18^ | 18 baboons | Two arm study | Healthy baboons anesthetized with thiopentone sodium, phencyclidine and suxamethonium | Primary: Test the effects of NE on cerebrovascular activity  Secondary: Effect of hypertonic urea |
| Chandra et al, 1972^20^ | Not specified | Four arm study | Healthy cats were anesthetized with pentobarbital sodium | Primary: Choroidal blood flow and the effects of autonomic agents |
| Muravchick et al, 1976^21^ | 26 cats | Eight arm study | Healthy mongrel cats anesthetized with pentobarbital | Primary: Adrenergic receptors and vascular resistance in cerebral circulation  Secondary: Effect of catecholamines on CBF and CVR |
| Lobato et al, 1980^22^ | Not specified | Non randomized control study | Healthy cats anesthetized with sodium pentobarbital and intraperitoneally with vessel change measured in removed brains | Primary: Cerebrovascular reactivity to NE and serotonin following experimental subarachnoid hemorrhage |
| Tomita et al,1979^23^ | 23 cats | Four arm study | Healthy and cranial hypertensive cats anesthetized with urethane and chloralose | Primary: Distensibility of cerebral vessels in response to acute hypertension  Secondary: Blood pressure response to NE and papaverine |
| Haggendal et al, 1966^28^ | 11 dogs | Three arm study | Healthy mongrel dogs anaesthetized with pentobarbital | Primary: Effects of some vasoactive drugs on the vessels of cerebral grey matter in the dog  Secondary: In a few dogs, similar procedures were performed under the influence of induced slight hypoxia and/or hypercapnia. |
| Gabrielyan et al, 1970^29^ | Not mentioned | Non randomized control trial | Healthy dogs that were bleed anesthetized with nitrous oxide and oxygen | Primary: Effect of NE on rCBF depending on initial MAP |
| MacDonnell et al, 1971^30^ | 4 dogs | Three arm study | Healthy mongrel dogs anesthetized with sodium pentobarbitonal | Primary: Factors affecting response of CBF and cerebral metabolism to NE infusion |
| James et al, 1975^31^ | 37 dogs | Seven arm study | Healthy mongrel dogs were anaesthetized with sodium pentobarbitonal | Primary: Evaluate factors affecting the cerebrovascular response to NE in the dog |
| Ekstrom-Jodal et al, 1974^32^ | 21 dogs | Two arm study | Healthy mongrel dogs anaesthetized with thiopental and nitrous oxide | Primary: Effects of NE on CBF in dogs  Secondary: Effect of alpha-adrenergic blockers on NE and CBF |
| Rogers et al, 1989^42^ | 21 pigs | Four arm study | Healthy piglets anesthetized with halothane and with right common carotid artery ligated | Primary: Influence of intraarterial NE on cerebral hemodynamics of newborn pigs |
| Reynier-Rebuffel et al, 1986^56^ | 29 rabbits | Non randomized control study | Healthy rabbits - some anesthetized | Primary: Possible mediation of CBF response to systemic NE |
| Patel et al, 1990^57^ | Not mentioned | Three arm study | Healthy rabbits anesthetized with 1.0 MAC isoflurane | Primary: CBF and cerebral blood pressure during 1.0 MAC isoflurane anesthesia |
| Gannushkina et al, 1974^58^ | 22 rabbits | Two arm study | Renal hypertension in healthy rabbits | Primary: Effect of high blood pressure on CBF in renal hypertension |
| Tomomatsu et al, 1981^59^ | 62 rabbits | Two arm study | Healthy rabbits of either sex anesthetized with urethane | Primary: Increased activity of carotid sinus baroreceptors by sympathetic stimulation and NE |
| Edvinsson et al, 1979^61^ | 49 rats | Six arm study | Healthy adult male Sprague-Dawley rats anesthetized with halothane | Primary: Quantitative changes in rCBF of rats induced by alpha and beta adrenergic stimulants |
| Edvinsson et al, 1978^62^ | 46 rats | Four arm study | Healthy Sprague-Dawley rats anesthetized with halothane | Primary: Effect of exogenous NE on local CBF after osmotic opening of the blood brain barrier in the rat |
| Lasbennes et al, 1988^63^ | 52 rats | Three arm study | Healthy male Wistar rats anesthetized with halothane | Primary: Effect of monoamine oxidase inhibition on rCBF  Secondary: Effect of clorgyline on cerebral hemodynamics |
| Szabo et al, 1983^64^ | 59 rats | Four arm study | Healthy male rats anesthetized with pentobarbital sodium and immobilized with gallamine triethiodide | Primary: Effect of sustained NE infusion on CBF  Secondary: Effect of NE after alpha-receptor blockade |
| Tuor et al, 1986^65^ | 16 rats | Two arm study | Healthy male rats anesthetized with halothane | Primary: Effect of hypertensive agent on regional cerebral perfusion |
| Nemoto et al, 1996^66^ | 13 rats | Two arm study | Healthy male Wistar rats anesthetized with halothane, some given donor blood and induced mild hypothermia | Primary: NE activation of basal cerebral metabolic rate for O_2_ during hypothermia |
| Sato et al, 1987^67^ | 4-6 rats per 4 studies | Four arm study | Healthy Sprague Dawley rats anesthetized with urethane | Primary: Effect of L-DOPS vs NE on CBF |
| Mascia et al, 1999^68^ | 10 rats | Non randomized control study | Healthy Sprague Dawley rats anesthetized with halothane | Primary: To investigate the role of the endothelin system in pressure autoregulation of CBF in rats |
| Stromberg et al, 1992^69^ | 24 rats | Non randomized control study | Healthy male Sprague Dawley rats anesthetized with ketamine and acepromazine | Primary: Angiotensin in receptors regulate CBF in rats |
| Zhang et al, 1991^70^ | 16 rats | Three arm study | Healthy male Sprague Dawley rats anesthetized with inactin | Primary: Superoxide dismutase decreases mortality, blood pressure, and CBF responses |
| Gozzi et al, 2007^71^ | 35 rats | Four arm study | Healthy male Sprague Dawley rats anesthetized with halothane and nitrous oxide | Primary: Cerebral hemodynamics and autoregulation in pharmacological MRI  Secondary: Effect of NE on rCBF and MABP |
| Kuschinsky et al, 1983^72^ | 17 rats | Three arm study | Healthy male Dawley rats anesthetized with halothane with final values attend from removed brain | Primary: The effects of intravenous NE on the local coupling between glucose utilization and blood flow in the rat brain |
| Kraut et al, 2004^73^ | 9 rats | Three arm study | Healthy male Wistar rats anesthetized with equithesin | Primary: The effect of NE on tissue areas |
| **Healthy Lightly Anesthetized Animal Models** | | | | |
| Artru et al, 1981^33^ | 18 dogs | Four arm study | Unmedicated fasting mongrel dogs with succinylcholine infusion followed by endotracheal intubation (anesthetized with Nitrous oxide, Halothane, Pentobarbital or Ketamine) | Primary: Anesthetics affect the cerebral metabolic response to circulatory catecholamines |
| Lluch et al, 1973^39^ | 15 goats | Five arm study | Unanesthetized healthy female goats with thrombosis | Primary: Evidence for effects of adrenergic drugs on CVR  Secondary: The effect of amines on CBF |
| Perales et al, 1997^40^ | 14 goats | Three arm study | Conscious female goats sedated with ketamine | Primary: Effects of magnesium sulphate on the NE-induced cerebral vasoconstrictor and pressor responses in the goat |
| Von Essen et al, 1972^43^ | No Specified | Three arm study | Healthy dogs lightly anesthetized | Primary: Effects of dopamine, NE and 5-hydroxytryptamine on the CBF in the dog  Secondary: The effect of dopamine in the presence of pimozide or haloperidol |
| Edvinsson et al, 1972^55^ | 124 mice | Two arm study | Unanesthetized sympathetomy male albino mice | Primary: Sympathetic neural influence on NE vasoconstriction in brain vessels |
| **Animal Models with Ganglionectomy** | | | | |
| Alborch et al, 1977^41^ | 11 goats | Two Arm Study | Unanesthetized female goats with removed cervical ganglion | Primary: Effect of blood flow after removal of cervical ganglion  Secondary: The effect of NE, tyramine, phentolamine and propranolol on CBF |
| Aubineau et al, 1985^60^ | 7 rabbits | Three arm study | Ganglionectomy on rabbit anesthetized by diazepam-pentobarbital | Primary: Long-term effects of superior cervical ganglionectomy on cortical blood flow of non-anesthetized rabbits in resting and hypertensive conditions  Secondary: Effect of NE and angertoion II on blood flow |
| **Animal Models with Bile Duct Removed** | | | | |
| Bloom et al, 1975^19^ | 16 baboons | Non randomized control study | Bile duct removed in baboon anesthetized with ketamine hydrochloride and portion of them had their bile duct removed | Primary: Modification of the cerebrovascular response to NE by bile duct ligation |
| **Healthy Heavily Anesthetized Animal Models with Craniotomy** | | | | |
| Shalit et al, 1974^24^ | 32 cats | Non randomized control study | Craniotomy on healthy adult cats anesthetized with pentobarbital with balloon induced hypertension | Primary: Interrelationship between blood pressure and rCBF in experimental intracranial hypertension |
| Ulrich et al, 1985^25^ | 21 cats | Four arm study | Craniotomy on adult cats immobilized with pancuronium bromide and anesthetized with gluochoralose | Primary: In vivo effects of alpha-adrenoceptor agonists and antagonists on pial veins of cats |
| Wei et al, 1975^26^ | 47 cats | Six arm study | Craniotomy on anesthetized cats with sodium pentobarbital or urethane | Primary: Determinants of response of pial arteries to NE and sympathetic nerve stimulation |
| Busija et al, 1987^44^ | 16 pigs | Prospective randomized animal study | Craniotomy on newborn pigs of either sex 1-5 days of age, were anesthetized with ketamine hydrochloride and acepromazine | Primary: Eicosanoid synthesis elicited by NE in piglet parietal cortex  Secondary: NE and Isoproterenol effect on cerebral vessels |
| Leffler et al, 1989^45^ | 19 piglets | Prospective randomized animal study | Craniotomy on piglets anesthetized with ketamine hydrochloride and acepromazine | Primary: Postischemic cerebral microvascular responses to NE and hypotension in newborn pigs |
| Myburgh et al, 1998^54^ | 5 sheep | Three arm study | Craniotomy on female sheep, anesthetized | Primary: Comparison of the effect of NE, E and Dopamine on CBF and COU |
| Muir et al, 1993^74^ | 17 rats | Non randomized control study | Craniotomy on male Sprague rats anesthetized with sodium pentobarbital | Primary: Cocaine effect on blood pressure and CoBF (cortical) response to NE in rats |
| **Healthy Heavily Anesthetized Animal Models with Explanted Brains** | | | | |
| Oberdorster et al, 1973^34^ | 14 dogs | Three arm study | Dissected canine brains anesthesia with a mixture of allobarbital, urethane and ethylene urea, coagulation prevented with vetren | Primary: Direct effects of alpha and beta sympathomimetic amines on the cerebral circulation of the dog |
| Lowe et al, 1971^35^ | 12 dogs | Four arm study | Brains from mongrel dogs premedicated with  morphine sulfate and anesthetic with methoxyflurane | Primary: Demonstration of alpha and beta adrenergic receptors in canine cerebral vasculature |
| Zimmer et al, 1974^36^ | 6 dogs | Three arm study | Isolated perfused dogs brains which were intravenously anesthetized with a mixture of amobarbital and urethane | Primary: The effect of catecholamine on CBF and oxygen consumption in isolated perfused dog's brain |
| Omar et al, 2010^75^ | About 23 rats  for each study | Pharmacological study | Brains of Wistar rats Juvenile, mature and old | Primary: Age-related changes in the sympathetic innervation of cerebral vessels and in carotid vascular responses to NE in the rat in vitro and in vivo studies |
| Takahashi et al, 2000^76^ | 7 rats | Two arm study | Brains from male Wistar rats anesthetized with pentobarbital sodium | Primary: The vasoconstrictive action of NE and serotonin in deep arterioles in rat cerebral gray matter |
|  |  |  |  |  |
| **Various Animal Models** | | | | |
| Mori et al, 1999^27^ | 34 cats | Three arm study | Hypothermia induced in adult cats of both sexes anesthetized with halothane and continuous infusion of ketamine and pancuronium bromide | Primary: Misery perfusion caused by cerebral hypothermia  Secondary: Effects of vasopressor administration on misery perfusion |
| Panther et al, 1985^37^ | 8 dogs | Three arm study | Brain cancer dogs anesthetized with sodium pentobarbital | Primary: Vasoactive drugs produce selective changes to blood flow |
| Nakagawa et al, 1977^38^ | 21 dogs | Non randomized control study | Stereotaxic lesions made on hypothermic dogs anesthetized with thiamylal sodium and lesion | Primary: Role of posterior hypothalamus in the development of acute brain swelling  Secondary: Lesion effect on ICP |
| Miller et al, 1984^46^ | 17 pigs | Three arm study | Endotoxin shock induced in healthy pigs anesthetized with ketamine and pentobarbital | Primary: Vasopressors do not increase cerebral cortical blood flow in endotoxin shock |
| **Anesthetized Animal Models given CPR** | | | | |
| Prengel et al, 2005^47^ | 21 pigs | Prospective randomized animal study | CPR in domestic pigs anesthetized with pentobarbital | Primary: Effects of combined administration of vasopressin, E and NE during cardiopulmonary resuscitation in pigs |
| Hoekstra et al 1990^48^ | 14 piglets | Two arm study | CPR on pigs anesthetized with halothane and alpha-chloralose | Primary: The effect of NE vs E on CBF and myocardial blood flow during CPR |
| Brown et al, 1989^49^ | 5 pigs | Three arm study | CPR on pigs anesthetized with halothane | Primary: The effect of NE vs E on rCBF during CPR  Secondary: CBF effect of NE and E in the presence of adrenergic antagonist |
| Lindner et al, 1990^50^ | 21 pigs | Three arm study | CPR on pigs anesthetized with metomidate and buprenorphine | Primary: The effects of E and NE on cerebral oxygen delivery and consumption during open-chest CPR  Secondary: The effects of E and NE on CBF during open-chest CPR |
| **TBI Anesthetized Animal Models** | | | | |
| Armstead et al, 2016^51^ | 40 pigs | Three arm study | TBI juvenile pigs anesthetized with fentanyl, midazolam, dexmedetomidine and propofol | Primary: NE’s cerebral autoregulation effects TBI in juvenile pigs  Secondary: How NE protects cerebral autoregulation |
| Friess et al, 2012^52^ | 16 piglets | Three arm studies | TBI 4 week old piglets anesthetized with fentanyl and isoflurane | Primary: PE vs NE after non invasive brain trauma  Secondary: The effects of PE and NE in the young |
| Daley et al 2004^53^ | 6 piglets | Prospective randomized animal study | TBI in healthy piglets anesthetized with ketamine and acepromazine | Primary: Assessment of cerebrovascular autoregulation in uninjured and brain injured pigs |
| Ract et al, 2001^77^ | 14 rats | Three arm study | TBI in Sprague Dawley rats anesthetized with pentobarbital | Primary: Comparison of dopamine and NE after TBI and hypoxic-hypotensive insult |
| **Review Article** | | | | |
| Kovach et al, 1976^16^ | Not applicable | Systematic literature review | Dogs, cats, rats and humans | Primary: CBF and brain function during hypotension and shock |
| AT, Angiotensin II; CBF, cerebral blood flow; CBV, cerebral blood volume; ChBF, choroidal blood flow; CMOT, Catechol-O-methyltransferase; CMR_Gluc_, cerebral glucose uptake; CMRO_2_, cerebral oxygen consumption; CoBF, corticoid blood flow; COU, cerebral oxygen utilization; CO_2_, carbon dioxide; CP, cerebral perfusion; CPR, cardiopulmonary resuscitation; CPP, cerebral perfusion pressure; CSF, cerebral spinal fluid; CVR, cerebrovascular resistance; E, epinephrine; ERK, extracellular signal-regulated kinase; FPI, fluid percussion injury; HMF, highest modal frequency; ICP, intracranial pressure; IL-6, interleukin-6; keto-PGFaa, 6-keto-prostaglandin; L-DOPS, l-threo-3,4-dihydroxyphenylserine; L-NMMA, methylarginine; MABP, mean arterial blood pressure; ; MAC, minimum alveolar concentration; MAO, Monoamine oxidases; MAP, mean arterial pressure; ; MAPK, mitogen-activated protein kinase; MBF, mean blood flow; MDo, myocardial oxygen delivery; MRI, magnetic resonance imaging; MVo, myocardial oxygen consumption; NE, norepinephrine; PE, phenylephrine; PGE2, Prostaglandin E2; PO_2_, partial pressure of oxygen; rCBF, regional cerebral blood flow; SAH, subarachnoid hemorrhage; TBI, traumatic brain injury; TXB2, Thromboxane B2; 5-HT, 5-hydroxytryptamine; | | | | |

Table 2: Norepinephrine Treatment and Cerebrovascular Response – Study Details

| **Reference** | **Dose of Vasopressor Administered** | **Mean Administration** | **Technique to Measure Cerebrovascular Response** | **Cerebrovascular Response** | **Adverse Effects to Norepinephrine** | **Conclusions** |
| --- | --- | --- | --- | --- | --- | --- |
| **Healthy Heavily Anesthetized Animal Models** | | | | | | |
| McCalden et al, 1979^17^ | NE: 0.55ug/kg/min  1.1 ug/kg/min   COMT blockade, MAO blockade, Denervation | 60 mins | CBF: Radioactive microspheres with injections of Xenon^133^  CMRO_2_: Calculated with CBF | **PCO_2_ and PO_2_ remained constant throughout all groups**  **All values in ml/min/100g and are the alteration from baseline value NE 0.55ug:**  **CBF during:**  Control: +9.7 ± 3.6  COMT: -3.3 ± 6.1  MAO: +1.8 ± 4.0 (p<0.05) Denver: -9.0 ± 7.2 (p<0.05)  **CBF after 10 mins:** Control: +11.5 ± 2.2  COMT: -4.6 ± 2.2 (p<0.05)  MAO: +2.1 ± 2.7 (p<0.05) Denerv: -0.6 ± 4.6 (p<0.05)  **CMRO_2_:** Control: +0.1 ± 0.1  COMT: -0.3 ± 0.6 MAO: -0.4 ± 0.2 Denerv: -0.1 ± 0.6   **NE 1.10ug:** **CBF during:** Control: +15.5 ± 4.8 COMT: -10.6 ± 0.9 (p<0.05) MAO: +0.1 ± 2.2 (p<0.05) Denerv: -6.8 ± 8.2 (p<0.05)  **CBF after 10 mins:** Control: +14.1 ± 3.2 COMT: -7.3 ± 1.6 (p<0.05) MAO: +0.6 ± 2.3 (p<0.05) Denerv: -0.5 ± 4.5 (p<0.05) **CMRO_2_:** Control: +0.2 ± 0.3 COMT: -0.7 ± 0.5 MAO: -0.7 ± 0.2 (p<0.05) Denerv: +0.1 ± 0.7 | None Mentioned | The cerebrovascular uptake and degradation mechanisms may be efficient, this remains to be demonstrated by established in vitro technique. The extraneuronal COMT enzyme is important in limiting the access of blood-born NE to cerebrovascular constrictor receptors. |
| MacKenzie et al, 1976^18^ | NE: 40 ug/kg dissolved in 0.1m CSF 50 ng/kg/min after hypertonic urea | 10x every 20 min or 15s | CBF: Freely diffusible method with Xenon^133^  CMRO_2_: Standard enzymatic assay  Cerebral glucose uptake (CMR_glc_): Calculated by CBF *arteriovenous blood glucose difference | **PCO_2_ and PO_2_ remained constant throughout all groups**  **NE 40 ug kg:**  CBF: Increased by 1±2 ml/100g/min (P, NS)  CMRO_2_: Increased from 2.78±0.10 to 3.44±0.42 ml/100g/min (p<0.05) CMR_glc_: Increased from 4.21±0.42 to 10.65± 2.96 mg/100g/min(P, NS) No change no significant changes in CMRO_2_, CMR_glc_, CBF, or MABP  **Hypertonic Urea:** CBF: Decreased by 3±3 ml/100g/min (P, NS) CMRO_2_: Decreased by 0.04±0.18 ml/100g/min (P, NS) CMR_glc_: Decreased by 0.33±0.4 mg/100g/min (P, NS)  **NE and Hypertonic Urea:** CBF: Increased by 26±7 ml/100g/min (p<0.02) CMRO_2_: Increased by 0.79±0.11ml/100g/min (p<0.001) CMR_glc_: Increased by 4.84±1.67 mg/100g/min (p < 0.05). | None Mentioned | In two studies there wasn't any decrease in cerebral blood flow associated with the administration of NE. Once NE gains access to the cerebral interstitial fluid it would appear that the dominant circulatory response is vasodilation, this being accompanied by increased oxygen and glucose utilization by the brain. |
| Chandra et al, 1972^20^ | Levarterenol: 0.1-10 ug  E: 0.5 -1ug  Acetylcholine: 1-10ug  Isoproterenol: 0.01-1ug | Not Specified | Choroidal blood flow (ChBF): Krypton^85^ Clearance | **PCO_2_ and PO_2_ assumed to be constant throughout all groups**  **Levarterenol:**  Lateral long posterior axillary artery injection (LLI) low dose: CVR: +32% ChBF: -36% higher dose: CVR: +155% ChBF: No significant changes Femoral artery injection: CVR:+7% ChBF: +119%  **E:**  CVR: +33% ChBF: -38%  Systemic injection of low doses: CVR: +15% ChBF: -16%  High doses: CVR: -11% ChBF: +43%  **Acetylcholine Low dose:**  CVR: -35% ChBF: +30% High dose: CVR: -40% ChBF: +27% Systemic injection  CVR: -38% ChBF: +18%  **Isoproterenol:**  Lateral long posterior axillary artery injection: CVR: +7% ChBF: -27% decrease Femoral artery injection: CVR: Variable effect ChBF: -30% | None Mentioned | Autonomic agents have significant effects on CVR and ChBF indicating the presence of alpha and gamma receptors. In this respect, the choroidal vascular bed resembles that of other tissues except for the brain and retina. On the other hand, isoproterenol does not seem to have an appreciable effect on CVR indicating the absence of beta receptors. |
| Muravchick et al, 1976^21^ | NE: 0.5ug/kg  E: 1.0ug/kg  Isoproterenol: 2.0ug/kg  Histamine: 3.0ug/kg | 10-15s | CBF: Electromagnetic flow transducer and flow meter   CVR: Calculated by Net driving perfusion pressure/ observed perfusate flow rate | **PCO_2_ and PO_2_ remained constant throughout all groups**  **NE no blockade:**  CBF:-21.2 ± 2.0 (-25%) ml/min/100g CVR: +1.4± 0.9 (+82%) mmHg/ml/min/100 g  **NE alpha blockade:** CBF: -8.8 ± 2.0 (-8%) ml/min/100g CVR: +0.1± 0.0(+10%) mmHg/ml/min/100 g  **Isoproterenol no blockade:** CBF: +16.0± 1.2(+21%) ml/min/100g CVR: -0.4 ± 0.1( - 22%) mmHg/ml/min/100 g  **Isoproterenol beta blockade:** CBF: 0.0± 3.6 (0%)ml/min/100g CVR: 0.0± 0.1 (0%) mmHg/ml/min/100 g  **E no blockade:** CBF: -24.8 ± 2.0”(-29%) ml/min/100g CVR: +1.0± 0.2(+62%) mmHg/ml/min/100 g  **E alpha blockade:** CBF: -6.8 ± 1.6”(-7%) ml/min/100g CVR: +0.2± 0.1(+14%) mmHg/ml/min/100 g  **Histamine no blockade:** CBF: 35.7± 10.6(49%) ml/min/100g CVR:-0.6± 0.2(-30%) mmHg/ml/min/100 g  **Histamine beta blockade:** CBF: 27.8 ± 2.9(36%) ml/min/100g CVR: -0.5± 0.0(-28%) mmHg/ml/min/100 g  **Histamine alpha block:** CBF: 10.0 ±2.4(13%) ml/min/100g CVR: -0.2± 0.1(-11%) mmHg/ml/min/100 g | None Mentioned | The wide variation in absolute values of initial CVR presented in the data obtained with this preparation reflects the great sensitivity of the cerebral vasculature to the quality of the immediate biochemical and physical environment. The vasoconstrictor or vasodilator substance is a function of the initial vascular resistance.  NE demonstrated a general increase in CVR with a subsequent decrease in CBF |
| Lobato et al, 1980^22^ | NE: 10-8 to 10-4 (M)  5-HT: 10-8 to 10-5(2.5M) | Readjusted every 15 minutes during an equilibration period of 90 to 120 minutes | Isometric vascular responses: Grass force-displacement transducer | NE induced a dose-dependent contractile response of the posterior communicating arties of normal cats. This response was significantly reduced (p<0.02) in a competitive manner by phentolamine (10-6M), an alpha-adrenergic blocker.   For both NE the increase in the developed tension increases on a 0-300 mg tension, for all except SAH 3 days and ganglionectomy which both increase at the same rate from 100mg to 500mg or 140 to 500mg  For both 5-HT the increase in the developed tension increases on a 0-200 mg to 300-700 mg tension, for all except SAH 3 days and ganglionectomy which both increase at the same rate from 300mg to 1400 mg or 200 to 500 mg. | None Mentioned | Super sensitivity to NE and serotonin induced by subarachnoid hemorrhage (SAH) may be involved in the production of chronic cerebral vasospasm. |
| Tomita et al,1979^23^ | Papaverine hydrochloride:  10 mg/kg (n=6)  NE:10 ug/kg (n=9)  NE and acute brain swelling: 10 ug/kg (n=8) | To raise MABP to 150 mmHg | CBF: Calculated from CBV*density of brain tissue  CBV: Photodiode and polygraph   ICP: Strain gauge transducer | **PCO_2_ and PO_2_ was kept constant throughout all groups**  **Papaverine hydrochloride:**  ICP: Slight increase CBV: Increased by 1.4%  **NE:** Decrease in CBV in a cat without any premeditation, indicating that NE constricted the "inexperienced" cerebral vessels (p<0)  **NE and acute brain swelling:** CBV: Increased by 0.8±0.3% ICP: Increased by 15.3±3.3mmHg CBF: 91 to 101 ml/100g.min | None Mentioned | Intravenous administration of NE to papaverine-pretreated cats produced almost maximal distension of the cerebral vessels, together with simultaneous vasoconstriction in the peripheral vessels, giving rise to an uneven redistribution of blood between the brain and other non-essential organs of the body.  NE has an indication to constrict the brain vessels though this does not translate to a direct increase in CBF or ICP |
| Haggendal et al, 1966^28^ | Papaverine: 20-80 mg (n=6) 1-10 mg/kg/body weight  Papaverine and Aramine: 2 mg/kg and 30 ug/kg/min (n=4)  Aramine:200-500 ug/ml (n=8) infused at 1.5-40 ug/kg/body weight/min  NE:10-50 ug/ml (n=3) infused at 0.2-3 ug/kg/bodyweight/min | MAP: kept at 200 mmHg | CBF: Krypton^85^ clearance method  CVR: MAP/CBF | **PCO_2_ and PO_2_ was kept constant throughout all groups**  **Aramine:**  CBF: Reduced to 11 ml/100g/min  CPP: Increased **Aramine flow doubled:**  CBF: Reduce by 70%  CVR: 160% of control MAP: Increased by 50% **Papaverine and Aramine:** CBF: Increased to 160 ml/100g/min CVR: Decreased to 1 mmHg*100g*min/ml MAP: Constant at 180 mmHg  **NE 4ug/kg:** CBF: Decrease by about 40% CVR: 3x increase **NE 1ug/kg:** CBF: Decrease by about 40% CVR: 3x increase  **Hypotensive state:**  CBF was unchanged compared with aramine and NE thus indicating dilatation of the cerebral vessels as response to the decreased perfusion pressure.  **Papaverine 2 mg/kg:** CBF: Decrease by 10% CVR: Decreased by 0.4x Aramine provoked increase of CVR also existed when papaverine was given, although to a reduced extent. | None Mentioned | Aramine and NE, given as intravenous infusions in previous doses, had qualitatively similar actions on the cerebral circulation in dogs although NE consistently seemed to have a more potent vasoconstrictor effect. The cerebral vasoconstrictor effect of the pressor drugs were observed during slight hypoxia and/or hypercapnia. Papaverine was found to cause a marked vasodilatation of the cerebral vessels which also was obvious although less pronounced with Aramine. |
| Gabrielyan et al, 1970^29^ | NE: 24 ug/min | Not Specified | rCBF: Freely diffusible tracer Krypton^85^ infusion and correlated with PCO_2_   Blood Flow: Micro-Astrup instrument | **PCO_2_ and PO_2_ was constant throughout all groups**  **Control:** CBF: 0.85±0.016 ml/g/min,  Cerebral Resistance: 1.7±0.09 mm Hg/ml/100 g/min.   **Low hypotension caused by bleeding:** rCBF: Remained unchanged Cerebral Resistance: Decreased 1.28±0.009 mm Hg/ml/100 g/min (P <0.001)  **NE low hypotension:** CBF: Reduced by 0.60±0.023 ml/g/min (P < 0.001) Cerebral Resistance: Increased by 2.4 mg/ml/100 g/min | None Mentioned | NE on the rCBF is largely dependent on the initial value of the mean arterial pressure. Whereas in normotension, in response to injection of NE the CBF remains almost unchanged, in moderate hypotension it is considerably reduced |
| MacDonnell et al, 1971^30^ | NE 0.4 and 1ug/kg/min   Propranolol: 5mg  NE 1ug and Propranolol 5 mg | Several hours | CBF: Freely diffusible tracer injection of Krypton^85^  CMRO_2_: Oxygen electrode | **PCO_2_ and PO_2_ was kept constant throughout all groups**  **NE 0.4ug:** CBF: Slight drop CMRO_2_: Slight drop  **NE 1.0ug:** CBF: Slight drop CMRO_2_: Slight drop  **Propranolol:** CBF: Decrease 20% CMRO_2_: Decrease 10%  **Propranolol and NE:** CBF: Decrease 40% CMRO_2_: Decrease 30% | None Mentioned | NE alone will slightly decreased CBF but with the introduction of propranolol a more prominent fall of CBF. |
| James et al, 1975^31^ | NE: 0.1-1ug/kg/min  Propranolol: 0.4ug/kg/min  Phenoxybenzamine: 1-10 mg/kg | 15 to 60 mins | Cortical blood flow (CoBF): Freely diffusible tracer injection of Krypton  CMRO_2_: Product flow and the arteriovenous difference | **PCO_2_ and PO_2_ was constant throughout all groups except CO_2_ modulated**  **Control:**  CoBF: 108.6±9.0 ml/100g/min CMRO_2_: 10.9±1.1 ml/100g/min  **NE control:** CoBF: Increase by in CO_2_ 130%  Decrease by in propranolol and phenoxybenzamine  CMRO_2_: 15.2±2.90 ml/100g/min Dose greater than 0.1 ug/kg/min had little further effect on CoBF  **NE and CO_2_:** CoBF: 93 ml/100g/min CMRO_2_: Fell compared to control  **NE and propranolol:** CoBF: -60%  **NE and phenoxybenzamine:** CoBF: -125% | None Mentioned | Cerebral vasodilatation observed following intravenous NE is relaxed and is triggered by chemoreceptors activity. Antagonism of the cortical dilatory effects if intravenous NE by raised PaCO_2_ is the intact animal must be at a site different from the peripheral chemoreceptors. |
| Ekstrom-Jodal et al, 1974^32^ | NE: 0.03 to 7.5 ug/kg/min   Phentolamine: 0.3 to 15 mg/kg/min | NE dissolved into 50 ug/ml  Dopamine dissolved in 10mg/ml | CBF: Radioactive gauss elimination method Krypton^85^  1.5hrs was waited till first measure was taken | **PCO_2_ was low in most models with some having a high value 80mmHg**  **NE:**  CBF: Max change any dose above 2ug/kg/min at 20% CMRO_2_: Reduced 40 to 70 %  **NE and Phentolamine:** CBF: Alpha-adrenergic receptors blocked so no flow reduction | None Mentioned | NE induced a flow reduction which seemed to be already maximal at a fairly low infusion rate of below 2 ug/kg/min. The blood flow reduction was practically the same in normo- and hypercapnia. |
| Rogers et al, 1989^42^ | NE: 100 ng/min (n=11)  Propanol: 1 mg/kg (n=5)  Prazosin: 1 mg/kg and Yohimbine: 1 mg/kg (n = 5) | Two 5 min infusions | CBF: Radiolabeled microsphere technique   CMRO_2_: Blood gas analyzer | **PCO_2_ and PO_2_ was kept constant throughout all groups**  **NE:** CBF: 72± 5 to 82± 8 ml/10 g/min  Cerebral Oxygen consumption: 2.75±0.17 to 3.11 ±0.29 ml *O_2_/100g/min  **NE and propranolol:**  No significant effect  **NE + prazosin + yohimbine:** Limits of CBF and O2 consumption | None Mentioned | Circulating NE may increase CBF via beta adrenergic-mediated stimulation of cerebral oxygen consumption during severe stress. |
| Reynier-Rebuffel et al, 1986^56^ | NE: 1ug/kg/min | 35 sec | CBF: Autoradiographic diffusible tracer technique with C-14 ethanol | **PCO_2_ and PO_2_ was kept constant throughout all groups**  **NE unanesthetized:** CBF: No significant change in cortical regions but the flow decrease 6 to 22% in other structures which were significant in nucleus, hypothalamus, colliculus and reticular  **NE anesthetized group 1:** Same as unanesthetized but in superior colliculus the response was inverted leading to significant increase in blood flow  **NE anesthetized group 2:**  General increase in CBF except caudate nucleus | None Mentioned | Showed that caudate nucleus but not thalamic or cortical regions reaction to circulating NE which can be specifically differentiated from the classical autoregulatory response to BP. Under anesthetized these changes in cerebrovascular reactivity appear to be linked to moderate change in systemic reactivity. |
| Patel et al, 1990^57^ | Angiotensin II (AT): 20 ug/ml  NE: IV 32ug/ml  PE:120 ug/ml | Used to increase MAP to 20%, 40%, 60% and 80% | CBF: Radiolabeled microsphere technique | **PCO_2_ and PO_2_ was kept constant throughout all groups**  All values in ml/g/min **AT:** CBF:0.78±0.07 Hemispherical CBF:0.75±0.07 Posterior Fossa CBF: 0.86±0.06  **NE:** CBF:0.67±0.04 Hemispherical CBF:0.65±0.04 Posterior Fossa CBF: 0.75±0.05  **PE:** CBF:0.73±0.06 Hemispherical CBF:0.70±0.05 Posterior Fossa CBF: 0.82±0.07 | None Mentioned | NE and PE may indirectly result in cerebrovascular vasodilation or AT has intrinsic cerebral vasoconstrictive effects during isoflurane anesthesia and therefore the cerebrovascular autoregulation should affect selected vasopressor. |
| Gannushkina et al, 1974^58^ | NE: 10ml of a 0.02% solution | 2-3mins | CBF: Hydrogen clearance method | **PCO_2_ and PO_2_ was assumed to be constant throughout all groups**  **NE:** CBF: Dropped from 108 to 32 ml/100g/min then remained stable  **NE and Renal Hypertension:** CBF had a slight increase at injection (182 ml/100g/min; P < 0.01), which then fell sharply to 40-50% of its initial value (32 ml/100g/min P < 0.01) In 2 animals there was the same rise as control | None Mentioned | Raising the pressure in control rabbits above 160-180 mmHg led to an increase in the CBF; in the rabbits with experimental renal hypertension this increase in blood flow began at higher levels of the arterial pressure and was quickly followed by a decrease to 40-50% of the initial blood flow. |
| Tomomatsu et al, 1981^59^ | NE: 10^-9^ to 10^-5^ g/ml  Phentolamine: 10^-6^g/ml | 10mins | Tension: Isometer transducer  Pressure: Electrode manometer | **PCO_2_ and PO_2_ remained constant throughout all groups**  **Phentolamine:** Concentration of 10^-6^ g/ml completely abolished the responses to NE (10^-9^ to 10^-7^ g/ml) how at higher volume NE tension increase maximum of 40%  **NE:** Linear increase in tension from 0 to 100% as dose increased | None Mentioned | In the presence of 10^-9^ g/ml NE, discharge frequency of all units significantly increased at a given pressure step when compared with the control, whereas NE at a high concentration (10^-6^ g/ml) did not produce significant changes in the discharge frequency. It is concluded that NE released by sympathetic nerve endings most likely acts directly on the baroreceptor nerve endings and sensitizes them. |
| Edvinsson et al, 1979^61^ | L-arterenol hydrochloride: 1ug/kg/min  L-epinephrine bitartrate: 1 ug/kg/min  L-isoproterenol hydrochloride: 0.5ug/kg/min   Phentolamine:1ug/kg/min | 01.ml/min at 10mins | rCBF: Autoradiographic diffusible tracer technique with C-14 | **PCO_2_ and PO_2_ was kept constant throughout all groups**  **NE:** No significant effect in thalamus mesencephalon and pons, all other region the CBF was reduced by 10 to 27% (p<0.05)  **E:** CBF changes similar to that of NE but not significant  **Phentolamine +NE:** Prevented any change to CBF  **Phentolamine+ E:** Vascular response markedly reversed, pons 92% and thalamus 74% and mesencephalon 45-46 % (P<0.001)  **Propranolol + isoprenaline:** Clearcut increases in regional blood flow was found in pons, mesencephalon, thalamus and caudate nucleus. The cortical regions and cerebellum only showed a tendency to flow increase, which was not statistically significant | None Mentioned | The presence and heterogenous distribution in the cerebrovascular bed of alpha- and beta-adrenoceptors that can be activated by sympathomimetics given systemically. If NE was allowed to pass the blood-brain barrier after osmotic opening with urea, an increased regional flow was obtained, probably due to a mechanism where the vasodilator effect secondary to activation of cerebral metabolism predominated over the direct vasoconstrictor effect of the amine. |
| Edvinsson et al, 1978^62^ | NE: 5 ug/kg/min  Propranolol: 25 ug/kg/min | 10mins | CBF: Autoradiographic diffusible tracer technique with C-14 ethanol | **PCO_2_ and PO_2_ was kept constant throughout all groups**  **Brain region: (Base line, After urea) ml/100g/min** Parietal cortex: 4.7±0.4, 16.6±3.0 (p< 0.01) Occipital cortex: 4.5±0.5, 17.5±3.6 (p< 0.01) Caudate nucleus: 2.8±0.4, 12.5±3.0 (p< 0.01) Thalamus: 2.7±0.4, 10.9±2.6 (p< 0.05) Mesencephalon: 39± 0.5, 3.9±0.5 (p> 0.05)   **NE and hypertonic urea:** CBF: Significant increase over 10 ml/100g/min in every area but mesencephalon on injection side as compared to non injection   **NE and hypertonic urea and propranolol:** CBF: Negated effects of NE | None Mentioned | The normally low penetration of NE into the brain was enhanced fourfold in those brain regions that showed Evans blue extravasation following the administration of hypertonic urea. In the same regions, the systemic administration of NE markedly increased local CBF, compared to the contralateral hemisphere that was unaffected by the injection of urea. This effect on rCBF was blocked by the beta receptor antagonist, propranolol. |
| Lasbennes et al, 1988^63^ | NE: 10 ug/ml (N=20)   Clorgyline: 1 mg/kg (n=9)  Clorgyline and NE: 1.9mg/kg and 1.5 ug/kg/min (n=8) | To achieve MAP of 121 and 171 mmHg | rCBF: Autoradiographic diffusible tracer technique with iodo-antipyrine | **PCO_2_ and PO_2_ was constant throughout all groups**  **Only Clorgyline with NE had statistically significant rCBF:** Frontal Cortex: 18±5 (p<0.05) Parietal Cortex: 15±5(p<0.05) Thalamus: 14±5 (p<0.05) Mesencephalon:15±5 (p<0.05) Pons: 16±5 (p<0.05)  **NE:** rCBF and MAP showed linear relationship at large infusions produced substantiation increase in CBF  **Clorgyline:** No significant effect to CBF or blood brain barrier perfusion at any injection amount | None Mentioned | Clorgyline administration alone did not significantly modify rCBF, but the subsequent infusion of NE induced an increase in rCBF in all structures investigated. |
| Szabo et al, 1983^64^ | NE: 10 ug/kg/min 2hr (n=8) 20ug/kg/min 1 hr (n=11) 20ug/kg/min 2 hr (n=11)  Phenoxybenzamine and NE: 5mg/kg and 20 ug/kg/min for 2 HR (n=10) | 1-2 hrs | CBF: Autoradiographic diffusible tracer technique with C-14 labeled iodo-antipyrine   CVR=MAP/CBF | **PCO_2_ and PO_2_ was constant throughout all groups**  **Control:** CBF: 0.86±0.03 ml/min/g CVR:1.70±0.06 mmHg*min*g/ml  **NE 10ug:** CBF: 1.18±0.05 (p<0.001) CVR: 1.49±0.07(p<0.05)  **NE 20ug for 1hr:** CBF: 0.91±0.04 CVR: 2.39±0.12(p<0.001)  **NE 20ug for 2hr:** CBF: 0.66±0.05 (p<0.001) CVR: 1.8±0.11  **Phenoxybenzamine and NE:** CBF: 1.48±0.07 (p<0.001) CVR: 0.94±0.05 (p<0,001) | Lethal outcome of shock with sustained NE blood concentrations and for infusions over 20 ug/kg/min longer than 2 hr effectively prevent cerebral autoregulation. | Supports the hypothesis that high concentrations of NE in cerebral blood vessels produced by activity might be an important factor in etiology of blood flow deficiencies. |
| Tuor et al, 1986^65^ | L-NA: 1-15 ug/kg,  Dopamine: 75-300 ug/kg/min | ABP maintained at 35 mmHg | CBF: Autoradiographic diffusible tracer technique with C14 iodo-antipyrine | **PCO_2_ and PO_2_ was constant throughout all groups**  **NE 5ug:** CBF auditory cortex : Decreased by 18±5% CBF cerebellar vermis: Increased by 66±29%  CBF pontine reticular: Increased by 38±13% CBF median: 15% (p<0.05)  **Dopamine:** CBF in rostral cerebral cortex, posterior parietal cortex and white matter: Greater than 65% (P<0.05) CBF Nuclei of lower brain stem: Less than 40% (p<0.05) CBF median: 44% | None Mentioned | The cerebrovascular response to hypertension appears to be dependent upon the catecholamine which is employed to elicit the elevation in arterial blood pressure. The present data provide clear evidence that hypertension induced by NE and that induced by dopamine have distinctly different influences on the cerebrovasculature. |
| Nemoto et al, 1996^66^ | NE: 0.269ug/min and 0.195ug/min (n=9)  Donor Blood Transfusion: 5-10ml (n=10) | 5 to 10 ml dose | CBF: Hydrogen clearance technique  CMRO_2_: Divisible into that associated with electroencephalographic | **PCO_2_ and PO_2_ was kept constant throughout all groups**  **NE 38°C 0.269ug/min:** CBF: 132±27 ml/100g/min CMRO_2_: 7.48±2.49 ml/100g/min  **NE 34°C 0.195ug/min:** CBF: 121±24 ml/100g/min CMRO_2_: 5.41±2.02 ml/100g/min (p<0.001)  **Donor Blood 38°C:** CBF: 98±28 ml/100g/min(p<0.05) CMRO_2_: 7.41±1.78 ml/100g/min  **Donor Blood 34°C:**  CBF:101±32 ml/100g/min CMRO_2_:6.31±1.41ml/100g/min(p<0.001) | None Mentioned | NE infusion during hypothermia could nullify the beneficial effects of mild hypothermia in cerebral protection.  NE slightly decrease CBF in both situations |
| Sato et al, 1987^67^ | L-threo-3,4-Dihydroxyphenylserine (L-DOPS):  3 mg/kg and 1 mg/kg  L-DOPS and benserazide: 3 mg/kg and 3 mg/kg/hr  L-DOPS and propranolol: 3 mg/kg and 3 mg/kg/hr  NE: 100ug/kg/hr | 3 mins | CBF: Hydrogen clearance method | **L-DOPS 3m/kg CBF:** Increased in striatal blood flow(SBF)  **L-DOPS 1 mg/kg CBF:** NS effect  **L-DOPS and benserazide:** CBF increase was inhibited by benserazide  **L-DOPS and propranolol:** CBF increase was inhibited by propranolol  **NE CBF:** Marked increase to 40% at 20 mins then remained constant | None Mentioned | The effects of L-DOPS may be attributed to the action of NE formed from L-DOPS, and the action may be mediated by stimulation of beta-adrenoceptor.  NE increase CBF maybe due to cardiac output increase |
| Mascia et al, 1999^68^ | NE: 0.08 mg /kg/min | 30 mins x 2 | rCBF: Hydrogen clearance technique  PO_2_: Blood samples | **PCO_2_ and PO_2_ was constant throughout all groups**  **NE:** CPP: Increased by 21± 2 (23 ±2%) mmHg (p < 0.001)  CBF: 3.6±3.1 (6±8%) ml/100 g/min (p = 0.5)   **NE + endothelin-1 :** CPP: Increased by 18±1 (20±2%) mmHg (p < 0.001)  CBF: 15.8±4.1 (46±13%) ml/100 g/min (p = 0.004)  PO_2_: no significant change in any group | None Mentioned | Endothelin-1 production is required in the CBF response to increased CPP, but is not required in the maintenance of resting CBF.  NE increased CBF to a higher amount in the endothelin-1 group, indication its effect on cerebral response |
| Stromberg et al, 1992^69^ | PD123319: 1-10 mg/kg  NE: 0.1-3.2ug/min | To increase hypertension 5 mins before PD was injected | CBF: Laser-doppler flowmetry | **NE:** CBF increased from 110 to a max of 160% (p<0.01)  **PD 1 mg/kg +NE:** increased from 90 to 150% (p<0.01)  **PD 10 mg/kg + NE:** remain relatively stable from 120 to 110% (p<0.001) | None Mentioned | PD did not alter baseline CBF at normal pressures, but appears to interfere with autoregulatory mechanisms of CBF. The participations of alpha 2 receptors in the regulation of CBF confirms a physiological role for this receptor subtype and may give clues for future treatment of various cerebrovascular disorders.  NE increase CBF but maybe due to cardiac output then local ICP change |
| Zhang et al, 1991^70^ | NE Increasing doses: 0.01-30 ug/kg   Superoxide dismutase: 24,000 units/kg plus 1,600 units/kg/min | 300-400 g | CBF: Laser-doppler flowmetry  PO2: Blood samples | **PCO_2_ and PO_2_ was kept constant throughout all groups**  **NE 3ugkg:** CBF: Increased by 300% (p<0.03)  **NE 10ukg:** CBF: Slightly more than 3ug/kg but not significantly(p<0.03)  **NE and Superoxide Dismutase:** CBF: Similar to NE as injection (p<0.03) | Whereas five (63%) of the eight control rats died after the 10 ug/kg norepinephrine dose, all eight rats treated with superoxide dismutase survived this dose. | Blood pressure and CBF responses to submaximal pressor doses of NE and reduces mortality associated with acute hypertension in rats |
| Gozzi et al, 2007^71^ | NE: 0.125 ug/kg (n = 5) 0.5ug/kg (n =5) 2 ug/kg (n =5) 8 ug/kg (n = 5)  NE doses refer to the salt form of the compound | Over 80s | MAP: MRI acquisitioner   CBV: Laser doppler flowmetry, and MRI | **PCO_2_ and PO_2_ remained constant throughout all groups**  **NE 0.125 and 0.5ug/kg:** rCBV: No significant changes were observed   **NE 2ug/kg:** rCBV: Short lived microvascular rCBV increases started to appear in some of the VOIs, focal areas of significant activation were apparent in the cingulate and retrosplenial cortices alongside the sagittal sinus  **NE 8 ug/kg:**  rCBV: Raised up to 15% (p<0.01) | None Mentioned | CBF autoregulation was maintained over a BP range of 60–120 mmHg. Under these conditions, no significant central rCBV responses were observed, suggesting that microvascular rCBV changes in response to abrupt changes in perfusion pressure are negligible within the autoregulatory range. Larger BP responses were accompanied by significant changes in both CBV and CBF that might confound the interpretation of pharmacological MRI results. As the dose of NE was increased and MABP exceeded 130 mmHg. For MABP greater than 130 mmHg both LDF and microvascular rCBV showed transient but significant increases. |
| Kuschinsky et al, 1983^72^ | L-NE: 1 mg/100ml saline containing 0.1% ascorbic acid at 10-100ul/min (n=4)  2 Deoxyglucose: 50 uCi/kg(n=4)  Iodo Antipyrine: 50 uCi/kg (n=6) | Adjust to maintain stable heart rate | rCBF: Diffusible tracer with 14C amino antipyrine  Local rates of cerebral glucose utilization (LCGU):Calculated from the local tissue concentrations | **PCO_2_ and PO_2_ was constant throughout all groups**  rCBF during NE increased in most of the structures  LCGU: - 10 % and + 74 % (P < 0.05) in only 6 of 39 structures  Despite this large variability, there was still a tight correlation between the rCBF | None Mentioned | When compared to the relationship between LCGU and rCBF in a control group the slope of the regression line was increased significantly by NE, indicating a resetting of the coupling mechanism. At a given metabolic rate, a higher blood flow is needed to perfuse a brain structure during NE infusion than during control conditions. |
| Kraut et al, 2004^73^ | NE 5 ug/100g | 60 sec | CBF: Laser Doppler flowmetery | **NE cerebral tissue blood Flow:** Increased by 270±47% (p<0.05) | None Mentioned | The significant correlation between the hemodynamic state of the organs and its mitochondrial redox state may serve as an indicator of tissue vitality under "brain sparing" conditions  NE was seen to increase CBF in almost all regions |
| **Healthy Lightly Anesthetized Animal Models** | | | | | | |
| Artru et al, 1981^33^ | E: 0.1 and 0.25 ug/kg/min   NE: 0.25ug/kg/min | 40 min injection 3 times with 20 min rest | CBF: Determined by weighing timed collections and assuming the specific gravity of blood to be 1.05  CMRO_2_: Derived from measurements of arterial-cerebral venous (sagittal sinus) blood oxygen content differences | **PCO_2_ and PO_2_ remained constant throughout all groups**  **Cyclopropane Control:**  CBF: 67±7 ml/min/100g CMRO_2_: 4.33±0.49 ml/min/100g  **30-40 min with E 0.1ug/kg:**  CBF: 113±17 ml/min/100g (p<0.05)  CMRO_2_: 5.07± 0.57ml/min/100g (p<0.05)  **90-100min with E 0.25ug/kg:**  CBF: 62±12ml/min/100g  CMRO_2_:4.80±0.66ml/min/100g (p<0.05)  **150-160 min with NE 0.25 ug/kg:**  CBF: 63.0±15 ml/min/100g  CMRO_2_: 5.32±0.93ml/min/100g (p<0.05) Overall increased CMRO_2_ by 17-23% within 10-30 min  **Nitrous oxide, Halothane, Pentobarbital or Ketamine:** Regardless of anesthetic, each infusion of E or NE resulted in an immediate increase in CBF which, except with E 0.1ug/kg/min which returned to control levels within 10 min No change in CMRO_2_ regardless of dose or duration of infusion | None Mentioned | Cyclopropane but not the other anesthetics tested increased the permeability of the BBB and presumably allowed passage of E or NE into the brain to increase CMRO_2_, reversibly. Opening of the BBB may be a direct effect of cyclopropane on endothelial cells or may be mediated by central adrenergic systems. For their part, E or NE may increase CMRO_2_, by either direct action on neuronal receptors or metabolically coupled synaptic events.  NE increase CMRO_2_ and CBF in all anesthetic methods tested |
| Lluch et al, 1973^39^ | E:0.1 to 5 ug (n=10)  NE: 0.1 to 5 ug (n=10)  Isoproterenol: 0.01 to 1 ug (n=9)  phenoxybenzamine: 200 to 400 ug   propranolol: 250 ug | Until all gone | CBF: Radioactive gas elimination method  CMRO_2_: Polyethylene Catheter | **PCO_2_ and PO_2_ was constant throughout all groups**  **E CBF:** Decrease of 55 ± 3% **E and phenoxybenzamine CBF:** Decrease of 15 ± 4%  **NE CBF:** Decrease 55 ± 3% **NE and phenoxybenzamine CBF:** Decrease 15 ± 5%  **Isoproterenol CBF:** Increases increment of 75 ± 6% **Isoproterenol and propranolol CBF:** Increases increment of 12 ± 1% | None Mentioned | E, NE, and isoproterenol exert powerful direct effects on the cerebral circulation of the unanesthetized goat, and these effects appear to be mediated by alpha and beta receptors. |
| Perales et al, 1997^40^ | NE: 10 ug/min 30 ug/min  Magnesium sulphate (MgSO_4)_: Infused intravenously at 0.3 g and 3 g | 15 mins | CBF: Electromagnetic flow probe  MAP: Catheter in femoral artery   CVR: Calculated as the mean arterial blood pressure in mmHg divided by CBF | **PCO_2_ and PO_2_ was not monitored**  **NE 10ug:** CBF: 55% CVR: 190%  **MgSO_4_ 0.3g and NE 10ug:** CBF: Increase to 61% at 5 mins then constant (p<0.01) CVR: Reduced to 178% at 5 mins (p<0.01)  **MgSO_4_ 3g and NE 10ug:** CBF: Increase to 80% at 5 mins then constant (p<0.01) CVR: Reduced to 120% at 5 mins (p<0.01)  **NE 30ug:** CBF: 80% CVR: 160%  **MgSO_4_ 0.3g and NE 30ug:** CBF: Increase to 90% at 5 mins then constant (p<0.01) CVR: Reduced to 140% at 10 mins (p<0.01)  **MgSO_4_ 3g and NE 30ug:** CBF: Increase to 110% at 10 mins then constant (p<0.01) CVR: Reduced to 90% at 10 mins (p<0.01)  Contraction was on average 10% less in MgSO_4_ and NE then NE alone | None Mentioned | Magnesium sulphate reverses the NE-induced cerebral vasoconstrictor and pressor responses by a direct inhibitory action of Mg2+ on the actions of NE in the cerebral and peripheral vascular beds, which leads to a decrease in vascular resistance. |
| Von Essen et al, 1972^43^ | NE: 0.03 to 7.5 ug/kg/min  5-HT: 0.1 to 22.8 ug/kg/min  Dopamine: 0.05 to 57.4ug/kg | Not Mentioned | CBF: Radioactive gas elimination method | **PCO_2_ and PO_2_ was not monitored**  **NE:** CBF: Max reduction -21% (P=0.01) CMRO_2_: Constant  **5-HT:** CBF: +28% (p<0.01) CMRO_2_: Constant  **Dopamine low dose:** CBF: -20%  CMRO_2_: Constant Blocked with pimozide or haloperidol  **Dopamine high dose:** CBF: +30% (p<0.01) CMRO_2_: Constant Blocked by pimozide or haloperidol but not by propranolol | None Mentioned | Importance for the understanding of some circulatory disturbances of the brain and also for a correct interpretation of altered concentration of different amines, and their metabolites, in brain tissue and cerebrospinal fluid after administration of certain biogenic amines or their precursors. |
| Edvinsson et al, 1972^55^ | Tyramine: 0-10 mg/kg  NE: 5ug/kg | 2mins | CBV: Radioisotope dilution technique | **PCO_2_ and PO_2_ was kept constant throughout all groups**  **Tyramine:** CBV: Decreased as dose increases with 12% at 0.1mg/kg (p<0.05)   **NE under 12 hrs:** CBV: No significant change  **NE over 24 hrs:** CBV: Reduced up to 33% (p<0.01) | None Mentioned | That a NE induced vasoconstriction in the circulation of the brain depends on the quantitative access of the amine to the adrenergic receptor area. The vasoconstrictor response may be influenced by such features as the amount of adrenergic innervation, the types of adrenergic receptors present, and the properties of the barrier. |
| **Animal Models with Ganglionectomy** | | | | | | |
| Alborch et al, 1977^41^ | Tyramine: 50-500ug   Norepinephrine: 0.03-3ug  Phentolamine: 1 mg   Propranolol:1 mg | 1mg in 1ml of saline for 10-15 mins | CBF: Electromagnetic flow transducer | **PCO_2_ and PO_2_ was not monitored**  **Tyramine CBF:** Decreased versed control 50ug: 10 to 1 %CBF (control vs tyramine) 100ug: 20 to 5 %CBF 250ug: 25 to 10 % CBF 500ug: 30 to 10%CBF  **NE CBF:** Increased verse control 0.01ug: 10 to 15%CBF (control vs NE) 1ug: 15 to 25%CBF  2ug: 25 to 45%CBF 3ug: 39 to 54%CBF  %CBF is the reduction percent of the CBF  **Phentolamine:**  CBF Before Removal: Increased by 31% CBF After Removal: Increased by 2%,   **Propranolol:** CBF Before Removal: Reduced by 14% CBF After Removal: Reduced by 4% | None Mentioned | There is an active participation of the perivascular sympathetic nerve endings in the overall regulation of cerebrovascular resistance. The effects of phentolamine and propranolol on cerebral blood flow before and after removal of the superior cervical sympathetic ganglion indicate that under normal conditions both alpha and beta receptors display a tonic adrenergic activity in the cerebral blood vessels.  NE decrease CBF in all doses with increase dose causing increased response |
| Aubineau et al, 1985^60^ | NE: 1.8 to 2.2 ug/kg/min  Angiotensin II (AT): 1.0 to 1.8ug/kg/min | 30s | CBF: Radioactive microsphere with helium and thermal clearance  PO_2_: Measure with probes samples | **PCO_2_ was kept constant throughout all groups**  **NE:**  CBF: Not significantly changed  PO_2_: Reduce by 9% (p<0.05)  **AT:** CBF: Reduced by 10%  PO_2_: Reduced By 9 % (p<0.001)  **Stim:**  CBF: Decrease 23.6 in heterolateral hemisphere and 22.2 ml/100g/min in homolateral  PO_2_: Reduced by 18 % (p<0.01) | None Mentioned | As in the peripheral circulation, chronic sympathectomy affects the equilibrium of the vascular smooth muscle fibers but that circulating amines play no compensatory role in the cerebral circulation because of the blood-brain barrier.  NE did not significantly change CBF |
| **Animal Models with Bile Duct Removed** | | | | | | |
| Bloom et al, 1975^19^ | NE: 8, 16 and 32 ug/min | 10 min | CBF: Xenon clearance method  Cerebrovascular Resistance (CVR): Calculated with pressure/flow | **PCO_2_ and PO_2_ was constant throughout all groups**  **NE 8ug:** CBF: Reduction 8.4±4.3 ml/100g/min (p<0.005) CVR: Decrease 0.21 ± 0.12 mmHg/ml/min **NE 8ug and Jaundice:** CBF: Reduction 9.48±2.63 ml/100g/min (p<0.005) CVR: Decrease 0.66±0.28 mmHg/ml/min  **NE 16ug:** CBF: Reduction 8.6±6 ml/100g/min (p<0.02) CVR: Increase 0.001±0.11 mmHg/ml/min **NE 16ug and Jaundice:** CBF: Reduction 10.9±4.4 ml/100g/min (p<0.02) CVR: Increase 0.9±5.56 mmHg/ml/min  **NE 32ug:** CBF: Reduction 1.97±4.6 ml/100g/min  CVR: Increase 0.425±0.17 mmHg/ml/min **NE 32ug and Jaundice:** CBF: Reduction 5.16±3.6 ml/100g/min  CVR: Increase 0.71±0.28 mmHg/ml/min | None Mentioned | Indicate that in baboons following ligation of the bile duct there is an altered cerebrovascular response to infused NE. Cerebral vasoconstriction was obtained with infusions of NE at 8 ug and 16 ug in the jaundiced animals, whereas dilatation was evident in the control animals. These findings suggest an increased cerebrovascular sensitivity to NE in the obstructive jaundice following bile duct ligation. |
| **Healthy Heavily Anesthetized Animal Models with Craniotomy** | | | | | | |
| Shalit et al, 1974^24^ | ICP balloon increase (n=18)  Brain swelling (n=8)  NE drip was increased to make 40 to 80 mmHg blood pressure | 10 to 15 min | rCBF: Krypton clearance method  ICP: Epidural transducer  PO_2_: Measured with electrode system | **PCO_2_ and PO_2_ was constant throughout all groups**  **Balloon increase:**  NE did not significantly affect ICP below if ICP was below 70 mmHg but does above  NE results in a significant spike increases for rCBF (0.7ml/gm/min) at each dose, with less effect result at ICP above 80 mmHg  **Brain Swelling:** NE did not significantly affect ICP below 80 mmHg but does above NE did not significantly affect the CBF | None Mentioned | An increase in blood pressure in intracranial hypertension is not a favorable compensatory mechanism designed to maintain brain function.  NE had no significant results of rCBF but in high ICP NE injection did increase CBF |
| Ulrich et al, 1985^25^ | Phenylephrine: 10-9 to 10-3 M(n=19)  Oxymetazoline: 10-9 to 10-3 M(n=21)  Prazosin: 10-8 to 10-4 (n=15)  Yohimbine: 10-8 to 10-4 (n=23)  NE: 10-7 to 10-4M(n=25) | Injection of full solution | Venous diameter (VD): Glass micropipette with sharpened tips were filled with the test solutions and mounted on a micromanipulator | **Phenylephrine VD:** 1 to -10 % at 10-3  **Oxymetazoline VD:** 2 to -8% at 10-5 then slightly increased  **Prazosin VD:** Venous diameter remains constant  **Prazosin and NE VD:** -15% to 0   **Yohimbine VD:** -15% to 0  **Yohimbine + NE VD:** -23 to -1% | None Mentioned | Since both alpha and alpha2 adrenoceptor agonists are less potent constrictors of pial veins than NE in vivo, a preferential use of alpha, or alpha2 adrenoceptor agonists cannot be recommended, if a therapeutic reduction of ICP or blood volume is desired. |
| Wei et al, 1975^26^ | NE: 0, 10, 20 and 100 ug/ml   Concentration of CSF was Calcium increase 10 mEq/l | Short and long periods of time | CBF: Free diffusible tracer technique   Bulb placed for sampling and ABP | **NE 0 ug/ml Small vessel diameter(um):** 44.4 ± 1.8  **NE 10 ug/ml:** 43.3 ± 1.9  **NE 100 ug/ml:** 44.3 ± 1.4  **Ca^2+^ and CSF:**  NE 100 ug/ml cased the only change in diameter from 43.3 ± 2.1 to 42.9 ± 2.4um  **Ca^2+^ and Mg^2+^ in CSF:**  **NE 0 ug/ml Small vessel diameter(um):** 49.8 ± 2.3  **NE 10 ug/ml:** 47.5 ± 3.1  **NE 100 ug/ml:** 47.0 ± 4.6  **Wahl solution:**  **NE 0 ug/ml Small vessel diameter(um):** 49.8 ± 2.3  **NE 10 ug/ml:** 48.2 ± 2.2  **NE 100 ug/ml:** 47.5 ± 2.0  For all Ca^2+^ levels and Mg^2+^ levels and Wahl solution all small pail arties changes similar with changes in NE | None Mentioned | The results imply a functional role for postganglionic autonomic fibers in CBF autoregulation. NE in high concentration is capable of producing substantially greater constriction of these vessels than by sympathetic nerve stimulation suggests that the potential exists for NE-induced reductions in CBF of considerable magnitude under abnormal conditions, such as in response to brain injury. |
| Busija et al, 1987^44^ | NE: 10^-6^ to10^-4^ M (n=18)  Isoproterenol: 10^-8^ to 10^-6^M (n=7) | 5 mins | Pial arteries were observed with a wild trinocular stereo microscope. Pial arterial diameter was measured with a television camera mounted on the microscope, a video monitor, and a video microscale | **PCO_2_ and PO_2_ remained constant throughout all groups**  **NE:**  Constricted pial arteries: 203±27um to 164±18 um (20±2%) (n = 21 vessels from 16 animals) at 10^-4^ M.  Concentration in CSF of 6-keto-prostaglandin B to increase from 768±91 to 1544±151 pg/ml, thromboxane B2 to increase from 188±37 to 269±38 pg/ml, and prostaglandin E2 to increase from 2067±448 to 6575±751 pg/ml  **Isoproterenol:**  Did not affect pial arterial diameter at 10^-5^ M, but dilated pial arteries by 28±3% at 10^-7^M and 32±2% at 10^-6^ M. At the same time, CSF levels of 6-keto-PGFaa, TXB2, and PGE2 did not change. | None Mentioned | NE elicits release of prostanoids from the cortical surface, and that these substances limit cerebrovascular constriction to NE. That sympathetic nerve stimulation and exogenous NE are able to have substantial constrictor effects on the cerebral circulation of newborn pigs, and our findings are consistent with an important role of the sympathetic nervous system in regulation of CBF in the newborn animal. |
| Leffler et al, 1989^45^ | NE: 10^-6^ to 10^-4^ M  In three groups sham-operated control(n=7), 2-3 hours post ischemia (n=6) and 24 hours post ischemia(n=6) | 20 mins | Catheters place in aortae for blood withdrawal and monitoring  Prelims experiments showed that blood pressure was reduce such that radiolabeled microspheres did not work  Observe pial arterioles with trinocular stereo microscope | **PCO_2_ and PO_2_ was kept constant throughout all groups**  **NE 10-4 M:** Decreased pial arteriolar diameters similarly in all three groups (27%, 28%, and 21%)  **Sham-operated group:** Hypotension increased cortical subarachnoid cerebrospinal fluid prostanoid concentrations Exhibited pial arteriolar dilation in response to hypotension (28% at 33 mm Hg)  **2-3 and 24 hours group:** Hypotension decreased pial arteriolar diameters (21% and 17%, respectively). No alteration to cerebral prostanoid | None Mentioned | After cerebral ischemia, autoregulatory pial arteriolar dilation in response to hypotension is absent, while vasoconstriction in response to NE is intact. |
| Myburgh et al, 1998^54^ | Dopamine: 0-60 ug/kg/min  E: 10,20,40,60ug/kg/min  NE: 10,20,40,60ug/kg/min | 5mins | CBF: Ultrasonic doppler transducer  ICP: Intraparenchymal strain gauge catheter  Cerebral oxygen utilization COU: Signa CBF an auto-venous oxygen content difference | **PCO_2_ and PO_2_ was assumed to be constant throughout all groups**  **Dopamine:** ICP: Significant increase on does greater than 20 ug/kg/min  (78.6±13.1 to 97.2±8.8% CBF: Statistically significant rise in CBF after 40 ug/kg/min (13.2±3.2 to 52.6±24.3%) COU: Initial decrease at 20 ug/kg/min followed by increase to base line at 60 ug/kg/min  **E:** ICP: Dose dependent increase after 40 ug/min CBF: No significant change COU: No significant change  **NE:** ICP: Did not increase CBF: No significant change COU: No significant change | None Mentioned | Intact cerebral autoregulation model induced hypertension by E and NE is not associated which changes in CBF, where dopamine causes cerebral hyperaemia increased ICP and increased global cerebral oxygen utilization. |
| Muir et al, 1993^74^ | Ten minutes after cocaine (1 mg/kg, i.v.) or saline:  NE: increasing from 0.01-10 ug/kg | The pressor effect of L-NMMA was controlled for by comparison with NE titrated to effect an equivalent blood pressure elevation | Cortical blood flow (CoBF): Laser-doppler flowmetry | **PCO_2_ and PO_2_ was kept constant throughout all groups**  Cocaine significantly potentiated the blood pressure and cerebral blood flow responses  **NE:**  CoBF: Increased at 10^-4^ug/kg to 40% and 10^-1^ ug/kg to 150% | None Mentioned | Cocaine causes a rapid, transient increase in blood pressure and CBF and potentiates the magnitude and duration of the pressure and flow response to NE. Repetitive blood pressure elevations in cocaine abusers is one of the proposed mechanisms leading to damage of cerebral vessels. |
|  |  |  |  |  |  |  |
|  |  |  |  |  |  |  |
|  |  |  |  |  |  |  |
| **Healthy Heavily Anesthetized Animal Models with Explanted Brains** | | | | | | |
| Oberdorster et al, 1973^34^ | E: 0.001-10 ug (n=5)  NE: 0.001-10 ug (n=5)  Isoprenaline: 0.001-10 ug (n=5) | 30s | CBF: Photoelectric drop recorder  CVR: Calculated with CBF and internal perfusion pressure   ICP: Isolated with two pressure transducers | **PCO_2_ and PO_2_ remained constant throughout all groups**  **E and NE:**  Dose dependent increase of CVR ranging from 2% to 61% CVR: NE could be reversed by phentolamine, E were increased by propranolol CBF decreases linearly with inject from 0 to -5 ml/100g/min  **Isoprenaline:** Dose dependent decrease of CVR ranging from -5%to - 51%  CVR effect could be prevented by propranolol CBF Increase to 12 ml/100g/min at 1ug then remain relatively stable  The dilator potency was as follows: Isoprenaline : Epinephrine : Norepinephrine = 1 : 0.5: 0.3  The constrictor potency was as follows: Epinephrine : Norepinephrine : Isoprenaline = 1 : 0.5: 0 | None Mentioned | These sources of contamination cannot account for the vasomotor responses and that, consequently, both alpha and beta adrenergic activity of the cerebral vessels of the dog has been demonstrated.  NE increase CVR and decrease CBF which can be mediated with phentolamine |
| Lowe et al, 1971^35^ | Phenylephrine: 50-200ug   Isoproterenol: 15-40 ug  NE: 15-100ug  E: 15-100ug | Until dose gone | CBF: Maintained with pump  Pulsatile perfusion pressure: Recorded with servo channel of a Gilson five-channel polygraph  CVR: Calculated by mean perfusion pressure/CBF | **PCO_2_ and PO_2_ remained constant throughout all groups**  **Phenylephrine:** CVR: Increased over each dose increase **Phenylephrine and Phenoxybenzamine:** CVR: Less effective   **Isoproterenol:** CVR: Decreased, no apparent correlation to dose **Isoproterenol and propranolol:** CVR: Reduced effectiveness  **NE:** CVR: Increased with no apparent correlation to dose **NE and phenoxybenzamine:** CVR: Reduced response  **E:** CVR: Increased with no apparent correlation to dose **E and phenoxybenzamine**: CVR: Decreased **E and propranolol:** CVR: Increased |  | As catecholamine blood levels in intact dogs are low in comparison to those achieved in these studies, it appears doubtful that circulating catecholamines play an important physiological role in the regulation of CVR. Possible explanations are considered for the lower response of the cerebral vasculature to catecholamines when this response is compared to that observed in other vascular beds. |
| Zimmer et al, 1974^36^ | NE: 2ug/min  E: 2ug/min  Isoprenaline: 0.2ug/min | 10 mins | CBF: Photoelectric drop recorder  CVR: Calculated on pressure flow relationship  CMRO_2_: Changes in oxygenation in blood samples | **PCO_2_ and PO_2_ remained constant throughout all groups**  **In all groups the CVR and CBF effects are taken after indirect effects of drug are removed**  **NE:** CBF: Decreased by 0.2±6.0% (p>0.05) CVR: Reduced by 50% CMRO_2_: Not changed  **E:** CBF: Decreased 4.1 ± 3.3% CVR: Reduced by 50% CMRO_2_: Not changed  **Isoprenaline:**  CBF: Increased by 9.3 ± 3.6% CVR: Reduced by 50% CMRO_2_: Not changed  Note max CBF change was found within 1.5-2 mins and persisted to end of infusion | None Mentioned | Based on these investigations it is assumed that no pronounced vascular adjustments occur in the cerebral circulation during catecholamine infusions, however CBF is significantly affected by catecholamine. |
| Omar et al, 2010^75^ | NE: 2.5 ug/kg  Nitro-L-arginine methyl ester (L-Name): 10 mg/kg | To maintain ABP to 180 mmHg in mature and middle-aged   150 mmHg in juveniles rat | Carotid blood ﬂow (CoBF) and MABP: Transonic ﬂow probe | **PCO_2_ and PO_2_ was kept constant throughout all groups**  For all groups CoBF decreased after the injection of NE with a decrease of 0.5 ml/min (p<0.05) in mature and 0.5 ml/min (p<0.01) in middle age the juvenile only has a minor drop and it was not significant  Carotid vascular conductance (CVC) in all was significant at 0.005 ml/min (p<0.01) juvenile and 0.08ml/min (p<0.001) for mature and middle age rats  **L-Name +NE:** CoBF for juvenile and mature there was a slight decrease, in middle age there was a small increase CVC for juvenile and mature there was a slight decrease, in middle age there was a small increase | None Mentioned | The results of these two studies indicate that by middle age, ageing itself has already altered several key mechanisms that regulate the carotid circulation that includes the brain. |
| Takahashi et al, 2000^76^ | NE:10-7 to 10-5 M Yohinbin: 10-6M Prazosin:10-8M  5-HT: 10-10, 10-8, 10-6M Ketanserin: 10-6M Methiothepin: 10-6M | 5 min | Contractile diameter: Glass pipettes on micromanipulators monitored with video camera | **NE:**  As dose increases contractile diameter increases **Yohinbin + NE:** Significantly decrease control change(n=5, p<0.05) **Prazosin + NE:** Slight decrease in contractile change (n=5)  **5-HT:** Increase in control response with dose increase **Ketanserin + 5-HT:** Significantly dropped in contractile response (n=5 p<0.05) **Methiothepin + 5-HT:** Slight decrease in contractile response | None Mentioned | That 5-HT plays a significant role in arteriolar contractility only from the CSF side, while NE is an important regulator or regulator of arteriolar contractility from both the CSF and blood circulation sides.  NE causes dose dependent contractions of arterioles |
| **Various Animal Models** | | | | | | |
| Mori et al, 1999^27^ | Group A Hypothermia: (n=10)  Group B Hypothermia with NE: 6-30 ug/kg (n=6)  Group C Hypothermia with Barbiturate (thiopental): 5mg/kg (n = 6) | Increase Blood Pressure to 25 mmHg | CBF: Hydrogen clearance method  CMRO_2_: Calculated with arteriovenous oxygen difference and cerebral venous oxygen saturation taken from the superior sagittal  CVR: Calculated from (MABP - ICP)/CBF  CBV: Technetium-99m-labeled human serum albumin in 12 Ca | **PCO_2_ and PO_2_ was kept constant throughout all groups**  **Group A:**  CBF: 51.2±8.3ml/100g/min at 37°C and decreased with lower brain temperature (6.1 ± 2.7 at 25°C) CMRO_2_: 2.24 ± 0.75 ml/100g/min at 37°C was also decreased by 0.52 ± 0.20 at 25°C CBV: 5.3 ± 1.2% at 37 C decreased significantly at 29°C 3.7 ± 1.0% (p< 0.05) CVR: 3.2 ± 0.7 mmHg*ml/100g/min at 37°C increased significantly at 29°C 13.8 ± 5.2 (p < 0.01)  **Group B:** CBF: 24.2 ± 3.7 ml blood/ml O_2_ 24.6 ± 7.4 at 33°C 19.1 ± 4.3 at 25°C CMRO_2_: Proportional change associated with CBF  **Group C:** CBF/ CMRO_2_: Did not decrease | None Mentioned | These results suggest that hypothermia may cause vasoconstriction and misery perfusion in the brain. This potential risk of relative ischemia can be avoided by combination with vasopressor administration, that cerebral hypothermia may cause cerebral vasoconstriction and relative ischemia. To avoid this misery perfusion, patients should not be cooled below 31°C. Hypothermia combined with vasopressor administration may avoid this serious cerebral metabolic disturbance. |
| Panther et al, 1985^37^ | Adenosine: 4.94uM/kg  NE:0.7 ug/kg/min | Not mentioned | CBF: Radioactive microspheres  PO_2_: Blood samples | **PCO_2_ and PO_2_ was constant throughout all groups**  **Control:**  Cerebrum CBF: 58 ml/min/100g Tumor CBF: 1 ml/min/100g  PO_2_: 105mmHg  **Adenosine:** Cerebrum CBF: 10 ml/min/100g Tumor CBF: 100 ml/min/100g  PO_2_: 121mmHg  **NE:**  Cerebrum CBF: -1 ml/min/100g Tumor CBF: -23 ml/min/100g  PO_2_: 93mmHg | None Mentioned | Selective effects of adenosine and NE on blood flow to brain tumors may have important implications for chemotherapeutic treatment of brain tumors. Vasodilator drugs such as adenosine that selectively increase tumor blood flow, but not brain blood flow and may increase the therapeutic advantage of lipid soluble chemotherapeutic drugs. |
| Nakagawa et al, 1977^38^ | NE: 5ug/kg | 1.5-3mins | ICP: Pressure transducers  PO_2_: Blood samples taken | **PCO_2_ was kept constant throughout all groups**  All values in mmHg **NE:** Control: 125.0 ± 6.4 After needle insertion: 139.2 ±7.1  After first coagulation:167.7 ±12.7(p< 0.01) After second coagulation:133.8 ±9.8   **NE and Lesion:** Control: 396.0 ±25.4 After needle insertion: 346.0±9.2 NS After first coagulation: 362.0±17.5 NS After second coagulation: 342.2±20.8 NS  PO_2_ remains steady through out the experiments | None Mentioned | NE was not significant regardless of the level of the ICP, or of uni- or bilateral lesions of the hypothalamus.  NE resulting no significant change to CBF found from the ICP/PO_2_ relationship |
| Miller et al, 1984^46^ | NE: Not specified (n=6)  Dopamine: Not specified (n=5)  Phenylephrine: Not specified (n=6) | Endotoxin induced by bacteria for 40 min in   Dose to raise MABP to 70-80 mmHg | CBF: Radiolabeled microsphere technique | **PCO_2_ and PO_2_ was kept constant throughout all groups**  **NE, Dopamine, Phenylephrine:** Affected CBF similarly in all brain regions, with a decrease in brain total, cortex close to 27.1± 2.8 and 26.3±28 ml/min/100g where is the cerebellum slight decrease at 40.9±4.9. The brain stem increased by 41.8±4.7ml/min/100g (p<0.05) for all but compared to shock for last two.  **Control to Shock:** Brain:37.8±2.9 to 25.2±3.1 Cortex:36.1±2.7 to 22.9±2.8 Cerebellum: 47±3.6 to 30±8.4 Brainstem: 35.9±3.1 to 24.3±2.6 | Cerebellum and brainstem did not restore to control values with dose which may indicate underlying structural heterogeneity | Decreases in regional CBF with shock are similar to those reported by other, unchanged cortical CBF after injection suggest either an inability to auto regulate or disruption of the brain blood barrier resulting in vasopressor induced vasoconstriction which limits flow. |
| **Anesthetized Animal Models given CPR** | | | | | | |
| Prengel et al, 2005^47^ | E: 200 ug/kg  Vasopressin: 0.4 units/kg  NE + E + Vasopressin: 45 ug/kg, 45 ug/kg and 0.4units/kg | Up to 5mins | Organ perfusion: Radiolabeled microspheres technique | **PCO_2_ and PO_2_ was kept constant throughout all groups**  **CBF:(mL/min/100 g) Before, 90sec and 5 min after drug administration** **E:** 8±2, 23±3 and 17±3  **Vasopressin:** 11±3, 55±7 and 52±7 **NE+E+ Vasopressin:** 4, 67±13 and 53±12  (p < .05 at 90 secs and 5 mins vasopressin vs. E and vasopressin/E/NE vs. E).   CPP: Increased significantly after 90 sec in all drug administrations, with a decrease in E and NE+E+ Vasopressin group after 5 mins, vasopressin increased slightly after 5 mins  Two of seven animals in the epinephrine group, four of seven animals in the vasopressin/epinephrine/ norepinephrine group, and seven of seven animals in the vasopressin group could be successfully resuscitated | None Mentioned | Vasopressin with or without E and NE resulted in higher myocardial and cerebral perfusion than E alone, but there was no benefit in adding NE to vasopressin and E with regard to cardiac and CBF during cardiopulmonary resuscitation. |
| Hoekstra et al 1990^48^ | E: 0.2mg/kg(n=7)   NE: 0.20mg/kg(n=7) 0.08mg/kg 0.12mg/kg 0.16mg/kg 0.2mg/kg | 3.5mins | CBF: Radiolabeled microsphere technique | **PCO_2_ and PO_2_ was kept constant throughout all groups**  **During normal sinus rhythm:** CBF: No significant differences (p>=0.13)   **During CPR, LCBF and CBF:** NS differences (P>=.3)  **NE 0.2mg and E 0.2mg:** CBF: NE as higher by 0.2mg/kg but NS (P>=0.23)  **NE 0.08mg/kg CBF:** 3.7±3 ml/min/100g (P=NS) **NE 0.12mg/kg CBF:**13.5±1.4ml/min/100g (P=NS) **NE 0.16mg/kg CBF:** 23.7±24.5ml/min/100g (P=NS) **NE 0.2mg/kg CBF:** 16.8±14.6 ml/min/100g (P=NS)  **All drug administration:** CBF, MBF, MDo, and MVo, rose while ER fell in both E and NE with no significant differences between groups in CBF, ER, or intravascular pressures following drug administration (p<0.07).   **NE:** CBF: As dose increases there was an increase in CBF that stopped and went down after 0.16mg/kg, found in all brain areas CPP: Significant increase at 0.12mg/kg then a average decrease with increasing dose (p<0.05) | None Mentioned | NE 0.20 mg/kg is as effective as E 0.20 mg/kg at improving myocardial and CBF during CPR. NE 0.20 mg/kg improves MBF and MDo, over E 0.20 mg/kg, but any theoretical benefits of higher MBF and MDo, are offset by a proportional increase in MVo, in the NE treated animals. Dose lower than 02.mh/kg are probably more effective in the treatment of prolonged cardiac arrest. |
| Brown et al, 1989^49^ | E: 0.20 mg/kg (n= 5)  NE: 0.08 mg/kg (n= 5)   NE: 0.12 mg/kg (n= 5)  NE: 0.16 mg/kg (n= 5) | 30s | CBF: Radiolabeled microsphere technique | **PCO_2_ and PO_2_ was kept constant throughout all groups**  **E:**  rCBF: not statistically significant but superior then low doses of NE but medulla and cervical cord improved significantly  **NE 0.08 mg/kg (N = 5);**  Slightly increased vasoconstrictor effect  **NE 0.12 mg/kg and 0.16mg/kg:** Increased regional cortical CBFs by 12 mL/min/100g No significant increase to left cerebral cortex  **NE 0.16mg/kg:** Increased regional cortical CBF on the average above 23 mL/min/100g | None Mentioned | No significant difference in rCBF between the two highest doses of NE and E, 0.20 mg/kg, but these doses were superior to NE, 0.06 mg/kg, for improving flew to lower brainstem structures. That following a prolonged cardiac arrest, large doses of NE significantly improve CBF above that measured during CPR. Adrenergic agonists that contains a and B1 agonists but lacks B2 agonist properties may prove beneficial in this setting. |
| Lindner et al, 1990^50^ | NE: 45 ug/kg  E: 45 ug/kg | 90 secs and 5 mins | CBF: Radiolabeled microsphere technique  Cerebral Venous Blood and measure sagittal pressure: Catheter | **PCO_2_ and PO_2_ was kept constant throughout all groups**  **E (open chest CPR, 90sec and 5mins):** CBF: 30 ± 7 to 54±14 to 37± 17 mL/min/100g (p<0.05) Cerebral oxygen delivery: 4.3 ±1.2 to 7.4±1.7 to 5.1 ± 2.4 mL/min/100g (p<0.05) Cerebral Perfusion Gradient: 2.7± 0.5 to 4.4 ± 1.5 (p<0.05) to 3.3±1.2 kPa  **NE (open chest CPR, 90sec and 5mins):**  CBF: 30 ± 11 to 58 ± 22 to 45±21 mL/min/100g (p<0.05) Cerebral oxygen delivery: 3.7±1.4 to 7.3 ± 2.7 to 5.8 ± 2.7* mL/min/100g (p<0.05) Cerebral Perfusion Gradient: 2.5 ± 0.8 to 4.3 ± 1.2 to 3.9±0.5 kPa (p<0.05) | None Mentioned | NE and E after a five-minute cardiac arrest and three minutes of open-chest CPR led to the same increase in cerebral oxygen delivery more than cerebral oxygen consumption, and oxygen extraction decreased. Both are strong alpha and beta receptor stimulators, but in contrast to E, the beta effect of NE is weak.  NE demonstrated an increase in CBF and CMRO_2_ |
| **TBI Anesthetized Animal Models** | | | | | | |
| Armstead et al, 2016^51^ | Fluid percussion injury (FPI) post-treated with NE 0.7–1.3ug/kg/min  FPI post-treated with NE 0.7–1.3ug/kg/min + the ERK MAPK antagonist U 0126 1 mg/kg intravenously  Papaverine: 10^-8^ and 10^-6^M | CPP was targeted 65–70 mm Hg | CBF: Radiolabeled microsphere technique  CPP: MAP - ICP  ICP: Integra camino monitor and laser doppler probe  Transient hyperemic response ratio (THRR): Calculated by flow before compression/ release of compression | **PCO_2_ and PO_2_ was kept constant throughout all groups**  **Sham control:** CPP male: 70 ± 7mmHg CPP female: 71 ± 7 mmHg CBF male: No change CBF female: No change THRR male unilateral and bilateral: 1.15 and 1.27 THRR female unilateral and bilateral: 1.15 and 1.25  **FPI untreated:** CPP male: 45±4mmHg CPP female: 45±5 mmHg CBF male: Reduced by 20 ml/min/100g (p<0.05) CBF female: Reduced by 15 ml/min/100g (p<0.05) THRR male unilateral and bilateral: 1.04 and 1.10 THRR female unilateral and bilateral: 1.07 and 1.14  **FPI post-treated with NE:** CPP males: 68±5 mmHg CPP females: 66±5 mmHg CBF male: Reduced by 10 ml/min/100g (p<0.05) CBF female: No change (p<0.05) THRR male unilateral and bilateral: 1.14 and 1.21 THRR female unilateral and bilateral: 1.15 and 1.25  **FPI post-treated with NE + the ERK MAPK:** CPP males: 67±5 mmHg CBF male: No change (p<0.05) THRR male unilateral and bilateral: 1.15 and 1.25 No female data  Papaverine increase artery diameter in all groups | None Mentioned | NE protects cerebral autoregulation and limits hippocampal neuronal cell necrosis after FPI in both male and female juvenile pigs. In contrast, NE augmented ERK MAPK upregulation in newborn males but similarly blocked it in newborn females after TBI.  NE reduced CBF in male pigs with an increase in CVR in both sexs |
| Friess et al, 2012^52^ | NE and PE: 7.9±5.2 and 0.9±0.7 ug/kg/min titrated to CPP>70 mmHg | For 5 hrs | CBF: Thermal diffusion probe  ICP: Intraparenchymal monitors  PO_2_: Microdaylysis | **PCO_2_ and PO_2_ remained constant throughout all groups**  **PE:** CBF: Improves over time with peaks and valleys ranging 20 ml/100g/min  CPP: No significant change Greater reduction in cell injury  **NE:** CBF: Improves over time with peaks and valleys ranging 20 ml/100g/min CPP: No significant change PO_2_:Higher then PE  No ICP difference between groups at 70 mmHg | None Mentioned | NE resulted in greater increase in brain tissue oxygen tension than augmentation with PE, despite similar increases in CBF. |
| Daley et al 2004^53^ | NE: 1ug/kg/min | 5mins | CBF: Laser doppler flow meter velocity  Pail arteriolar: VHS recordings  ICP: Direct pressure monitor and femoral ABP recordings  CPP: ABP-ICP  HMF: Calculated from transfer from ABP to ICP | **PCO_2_ and PO_2_ was kept constant throughout all groups**  **Uninjured and NE:** An inverse relationship between HMF and CPP with a mean of 0.50±0.14 and 0.6±0.44 Hz/mm Hg CBF velocity: Decrease that remained relatively constant  **Injured and NE:** Direct relationship between HMF and CPP with a mean 0.48 ±0.21 and 1.13±2.08 Hz/mm Hg CBF: Increased after injury  **FPI:**  CBF:-3.64±12%  ICP: 61±32%  ABP: 43±24%  **After FPI:**  CBF: 8.47±20%  ICP: 44±28%  ABP: 58±26% | None Mentioned | Relating changes of HMF to changes of CPP may be of even greater value for evaluating the state of cerebrovascular regulation than evaluating changes in mean ICP induced by pressor challenge alone. However, the conclusions of this is only known to be applicable to a hypertensive challenge with NE under conditions of FPI obtained from an animal model with characteristics of diffuse axonal injury, and it might not apply to other situations or pathologies.  NE appeared to increase CBF after TBI but limited effect in healthy models |
| Ract et al, 2001^77^ | Dopamine: 5mg/ml (average: 274±110 ug/kg/min)  NE: 0.1-0.2 mg/ml (average: 18±4.5 ug/kg/min) | Started at 0.1ml/h and increased 0.1ml/h until CPP above 70 mmHg | CBF: Extradural laser Doppler fiber  ICP: Intraparenchymal fiberoptic device | **PCO_2_ and PO_2_ remained constant throughout all groups**  **Head trauma:** ICP: Remained constant at 27±18.5 mmHg CPP: Remained constant 28±22 mmHg CBF Decreases significantly from time 60 to 180 mins  **NE:** ICP: Increased to 40 mmHg at 30 mins then dropped slightly (p<0.05) CPP: Decreased over time after 15 mins to 10 mmHg (p<0.05) CBF: Decreased significantly similar to all other groups  **Dopamine:** ICP: Increased to 50 mmHg at 45 mins then stayed constant (p<0.05) CPP: No change CBF: Decreased significantly similar to all other groups | None Mentioned | NE and dopamine are not able to restore values of CPP above 70 mm Hg in a model of severe brain trauma and their systemic vasopressor properties are altered.  NE indicates no change to CBF |
| **Review Article** | | | | | | |
| Kovach et al, 1976^16^ | Various studies |  | CBF: Measured with a variety of methods including autoradiograph 14C, radioactive microsphere with Xenon clearance | Microinjection of NE into the hypothalamus of the rabbit caused increased flow at low concentrations and decreased flow at higher concentrations. One study observed marked CBF reduction after NE injection in hypercapnia. Three studies resulted in no CBF increase in the baboon in hemorrhagic shock upon administration of 6% CO_2_. In cross-circulation experiments in which the brain of the recipient dog was hemodynamically isolated from the trunk and perfused by a donor dog, intravenous E or NE injection into the recipient's trunk caused reflexly a significant increase in its total CBF. Intracarotid injection of both catecholamines produced a significant fall in CBF. Increased CBF could be measured during intravenous infusion of NE in hemorrhagic shock, while the cerebrovascular resistance showed no change. Increased CBF accompanied by increased cerebrovascular resistance followed NE administration during tourniquet shock. | None Mentioned | The reviewed results clearly suggest that vital functions of the brain in spite of the well-developed autoregulatory mechanisms are impaired during long lasting hypovolemic and other shock conditions. The insufficiency of the cerebrocortical and hypothalamic regulatory mechanisms can contribute to the development of the irreversible shock. In other words, failure of the body suffering from shock to restore the homeostatic equilibrium can be attributed to the inadequacy of the central nervous servo control system. |
| ABP, arterial blood pressure; AT, Angiotensin II; CBF, cerebral blood flow; CBV, cerebral blood volume; ChBF, choroidal blood flow; CMOT, Catechol-O-methyltransferase; CMR_glc_, cerebral glucose uptake; CMRO_2_, cerebral oxygen consumption; CoBF, corticoid blood flow; COU, cerebral oxygen utilization; CO_2_, carbon dioxide; CP, cerebral profusion; CPR, cardiopulmonary resuscitation; CPP, cerebral perfusion pressure; CSF, cerebral spinal fluid; CVR, cerebrovascular resistance; E, epinephrine; ERK, extracellular signal-regulated kinase; FPI, fluid percussion injury; HMF, highest modal frequency; hrs, hours; ICP, intracranial pressure; IL-6, interleukin-6; keto-PGFaa, 6-keto-prostaglandin; L-DOPS, l-threo-3,4-dihydroxyphenylserine; L-Name, Nitro-L-arginine methyl ester; L-NMMA, methylarginine; MABP, mean atrial blood pressure; ; MAC, minimum alveolar concertation; MAO, Monoamine oxidases; MAP, mean arterial pressure; MAPK, mitogen-activated protein kinase; MBF, mean blood flow; MDo, myocardial oxygen delivery; min, minute; MRI, magnetic resonance imaging; MVo, myocardial oxygen consumption; NE, norepinephrine; PE, phenylephrine; PCO_2_, partial pressure of carbon dioxide;  PGE2, Prostaglandin E2; PO_2_, partial pressure of oxygen; rCBF, regional cerebral blood flow; SAH, subarachnoid hemorrhage; sec, seconds; TBI, traumatic brain injury; TXB2, Thromboxane B2; x, multiplied by; 5-HT, 5-hydroxytryptamine; | | | | | | |
